# Supplementary figures and images for: Physiological growth of ocular axial length among Chinese children and teenagers: A 6-year cohort study
Source: PLoS One. 2025 Jan 24;20(1):e0317756. doi: 10.1371/journal.pone.0317756 (PMC11760619; doi:10.1371/journal.pone.0317756)

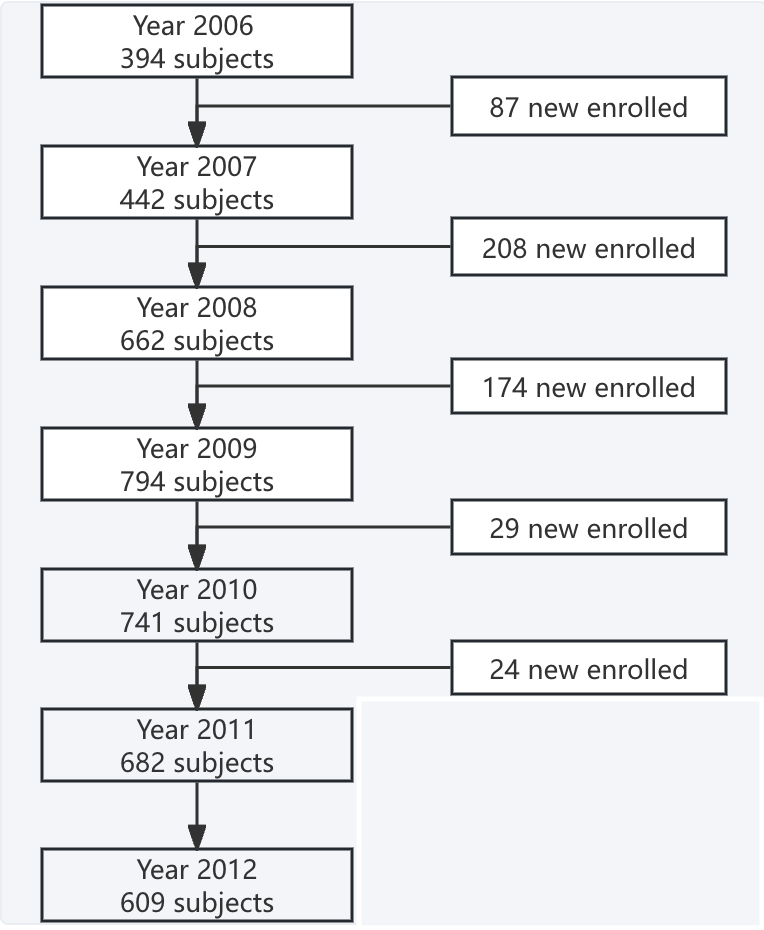

Supplement: S1 Fig — (TIF) [file pone.0317756.s001.tif]
