# Supplementary material for: Physiological growth of ocular axial length among Chinese children and teenagers: A 6-year cohort study
Source: PLoS One. 2025 Jan 24;20(1):e0317756. doi: 10.1371/journal.pone.0317756 (PMC11760619; doi:10.1371/journal.pone.0317756)
Supplement: S2 Data — (PDF) [file pone.0317756.s002.pdf]

gender	age_16	age_17	age_18	seod_6	age_6	seod_7
1				-1	14 17659	-1 125
1				0 75	12 19986	0 125
2				-1 18	10 88296	-1 875
2	20 86516	21 82615	22 89117	0 685	10 8063	0 25
2		23 66872		-1 62	12 66256	-2 125
1				-1 5	12 79671	-2
2						-1
2	24 85695	25 8371	26 84463	-1 055	14 78439	-1 75
1				1 37	11 59206	0 875
2				1 185	12 18617	1 25
2	20 89528			0 5	10 91307	-0 125
1				1 25	11 6961	1 375
1				2 065	12 03285	2
1				0 815	11 86037	1
2				0 625	10 3217	0 625
2	23 47433	24 56673	25 59343	-4 435	13 5332	-4 875
2				1	12 40246	1
2	19 40589	20 46817	21 50308	0 94	9 456536	0 75
2				0 1900001	10 58453	
2		22 62286		-0 625	11 60575	-0 875
1				0 5	11 72622	1 125
1				0 625	12 18344	0 375
2						-3 125
1				-3 125	14 99521	-3 875
2				-3 875	11 65229	
1		24 05202	25 05681	-3 25	13 0486	-3 25
2						
1			22 04244	0 12	9 976728	0
2	17 81246	18 80903	19 84668	0	7 882272	-0 875
1				0 375	10 75428	
1				1 25	11 17591	0 875
1				-0 25	14 86105	-0 75
1				1 5	9 798768	1 125
1			26 6037	-1 625	14 5243	-2 375
2				-0 375	11 32649	-1
1		21 71937	22 72964	0 75	10 67488	0 685
1				-0 75	16 07118	-1
2	22 74059	23 8193	24 87064	-2 56	12 79671	-3 875
1	20 38056			0 75	10 38467	0 625
2				-0 5	12 95003	-1
1	22 33265	23 32649	24 41068	-0 625	12 27378	-1 125
2	24 12594	25 12252		-4	14 07255	-5
2				0 375	12 10404	0 5
1		24 6653		0 815	13 6345	0 75
1						
1	18 70774	19 65503		1 25	8 706366	0 75
2						
2	22 03422	23 01985	24 03833	-2 875	11 96167	-3 125
1				-2 625	15 38672	-2 625
2	25 06776	26 10541	27 13758	-0 875	15 05544	-1 25
2	17 67283	18 69405	19 74812	0 625	7 753593	0 875
2						
2				1 875	11 80014	1 625

1				0 75	15 13758	0 875
2	18 51608	19 57016	20 59411	0 625	8 646132	0 125
2	18 66667	19 74538	20 76112	0 37	8 722793	0 25
1						
2	20 69268	21 70294	22 71869	0 25	10 6694	-0 875
1	17 72485	18 76249	19 7755	1 5	7 791924	1 5
2	20 45175	21 51951	22 58453	-3 75	10 49144	-5 25
2		21 18001		0 44	10 18754	-0 375
2	18 05886		20 13142	1 5	8 147844	0 875
2	22 02327	23 06366	24 07118	0 375	12 09856	-0 25
2	23 14305	24 23545	25 26489	0 0650001	13 22656	-1 375
2				-6 875	15 58932	-7 37
2				0 5	8 985626	-0 005
2				0	13 86721	-0 25
2				0 375	13 52772	0
2	18 32991	19 40041	20 45448			
2				0 75	9 136209	-0 5
2	22 20397		24 29569	0 625	12 2026	0 625
1	18 24504	19 26626	20 30938	-2 18	8 30664	-3 055
1				0 995	7 854894	0 995
1				1	11 72622	0 5
1						
1		25 86995	26 90212	-1 68	14 8282	-2 5
2	23 79466	24 82409	25 85353	-0 245	13 78782	-0 375
2				-0 13	14 31348	-0 75
2				-3 245	15 70431	-3 125
1		22 39562	23 45243	-1 305	11 38672	-2
1				1 25	10 7269	0 5
2	17 88638	18 93224		1 245	7 88501	1 125
1				#########	11 15127	-2 125
2	20 04928	21 14442	22 14647	-2 745	10 10814	-3 625
1				0 5	8 843258	
1		19 92608	20 95004	0 875	8 900753	0 5
1				-2 25	16 13142	-3 125
2		19 87953		0	8 928131	
2				1 125	14 09719	1 125
1		25 20739	26 2423	0 565	14 1848	0 375
1	24 87611	25 92471	26 9514	-1 5	14 95414	-2 125
1				-1	11 82204	-2
2				0 745	11 8193	0 625
2				-3	16 29295	-3 375
1				-4 435	15 96715	-4 75
1				-0 185	12 30116	-0 125
2						
1				-4 875	13 99042	
1	22 60096	23 58658	24 73101	-1 87	12 56947	-2 5
1				-0 375	12 51198	-0 625
2				-2 18	13 25941	-2 5
2	19 39494		21 47023	1 25	9 497604	1 125
1						
2	18 81177			-1 81	8 785763	-3 625
1	19 44422	20 47912	21 53046	-0 005	9 467488	-0 25
1						
2						

2				2	9 22382	1 625
1	18 5243	19 54278	20 60233	-3 12	8 5859	-4 125
1				-2 375	15 64956	-2
1	17 66461	18 68857		2 12	7 715263	2 125
1						
2	20 35044		22 34634	0 12	10 30801	-1
1						
2	23 97536	24 97741	26 02327	-2 435	13 97673	-2 875
2		25 74949		-3 5	14 78987	-3 875
1				-0 75	12 5065	-1 25
1				-0 245	15 38672	-0 75
1				1 815	10 23409	
2				0 625	13 577	0 75
1				0 5	13 15264	0 375
1				0 75	13 86995	
2		22 29706		-0 93	11 27995	-0 875
2	17 97399	18 9514	19 99179	1 56	7 983573	1
1				0 37	11 81109	0
1				0 5	9 798768	
1			23 21971	-1 25	11 20329	-2 25
2				-1 18	14 28337	-1 25
1			22 55168	0 5	10 51335	-0 125
2				-5 055	15 80287	-5 375
2				-0 555	12 30938	0
1				0 31	15 22793	0 5
2			26 90212	0 94	14 85284	1
2	21 04586		23 04723	5 81	11 01437	5 625
2	19 2334			1	9 275839	0 25
2	21 09514	22 12457				-3
1				-3 435	16 564	-3 75
1				0 87	7 646817	0 75
2						
2	17 88638			1 25	8 021903	0 75
1				0 25	12 78029	
1				1 435	7 693361	1 625
2				0 245	14 00684	0 375
2	24 70089	25 76591	26 78166	-5 555	14 75428	-6 25
1	21 295	22 30527	23 38398	-5 805	11 32375	-6 625
2						-0 5
2				0 81	8 870637	0
2	21 51951	22 55715		0 745	11 50719	0 875
1				0 25	15 43053	0 25
2	24 03012			1	14 02875	0 75
1						
2				1	12 68446	1
2	25 42916		27 55373	-5	15 54552	-5 625
1	19 34565	20 3614	21 39083	0 69	9 379877	0 75
1				1 81	8 963723	1
2		24 15879		0 69	13 17728	0 625
2	18 42026			0 125	8 451745	-0 5
2	18 54346			1 19	8 583162	1 5
2	19 85763	20 93634		1 125	9 941136	1
1	18 89391	19 89049	20 9473	1 375	8 936345	0 625
2	20 41068			0 06	10 49144	0

2	19 6742			1 995	9 73306	1 625
2				-0 25	11 40862	-0 375
1				-1 87	13 53867	-2 25
1	22 41205	23 44695	24 51472	-0 62	12 47365	-2 25
1			25 61259	-1 25	13 60712	
2				-3	13 36619	-4 25
1	18 38467	19 43327		0 935	8 449008	1
2				-0 81	12 59959	-1 625
1		26 80082	27 85489	-4 25	15 7974	
2		24 04107		1	13 05407	0 625
1		20 11499	21 12526	0 125	9 108829	-1 5
1				1 44	10 94319	1 125
1				1 065	8 451745	0 625
2				0 125	12 00548	0 125
1		20 95004	21 99042	-0 125	9 954825	-0 5
2				-3 81	14 94045	-4 375
2				-1 625	15 90144	-1 5
2		22 54073		1	11 58932	0 75
1	22 12183		24 18891	0	12 17248	-0 25
1				-1 375	9 667351	-2 875
2				1	9 22382	
1		21 31143		-2 125	10 34086	-3 5
1				-5 375	14 74606	
2	22 0178	23 01437	24 04654	-4 62	11 9781	-4 875
2				1 5	8 725531	1 25
2	20 04381	21 0705	22 0835	0 5	10 11088	-0 625
2		20 40246		0 25	9 453798	
1				-1 625	13 91376	
2		24 91171		0 875	13 92197	1
2						
1				1	9 711157	0 625
2				1 435	10 03696	1 375
1				-0 005	11 1102	-1 5
1	23 15948	24 16427	25 16906	-0 5	13 18275	-0 75
1				1 245	7 761807	0 75
1				0 625	10 65571	0 625
2	19 00068	19 92334	21 00753	0 5	8 99384	-1 125
1		26 09719	27 12936	-2 625	15 13484	-3
1				-1 18	14 94045	-2
2				0 435	11 06913	
2				-0 875	14 10267	-1 25
2	21 58795	22 61191	23 64956	-1 75	11 6742	-2 5
1				2 44	10 80904	1 625
1	18 25051	19 29911	20 33949	-0 44	8 399726	-1 25
2	21 67009		23 72074	-2 625	11 70705	-3 125
1				0 75	8 273785	0 875
1				0 75	9 538672	0 375
2				-7 055	14 04791	-7 75
1	18 10541	19 11567	20 18617	1 245	8 13963	1 125
1	18 2423	19 28816	20 33402	0 875	8 290213	1
1				1 065	16 44627	0 625
2			27 81656	-0 5	15 86311	-1
1		20 15606		-1	9 166325	-1 5
2				-2 125	13 95209	-2 5

2	17 79329	18 81451		1	7 84668	0 625
2				-1 25	13 81246	-1 625
1				-0 75	14 94045	-1 25
1	18 46407	19 52635	20 564	0 5	8 561259	0 25
1	18 92402	19 93155		0 375	8 941821	
1			21 40726	0	9 415469	0 25
1				-4 875	12 67077	-5 875
2	20 83504	21 87543	22 89117	-0 62	10 88296	-1
1				1 125	9 32512	
2		22 94319	23 96441	0 4400001	11 96167	0 625
2	19 98631	20 98289		-0 25	10 00137	-1
2		20 99384	22 02601	-1 625	10 00137	-2 125
2				-2	11 55921	-2 25
1				0 5	8 150581	-0 125
2						
1	23 92882	24 96372	25 98494	-5 12	13 96578	-5 5
2	19 30459	20 28474	21 36619	1 25	9 347023	1
1	20 07118	21 05955		-2 5	10 14921	-2 875
2						
2	25 06776	26 09993	27 15401	-2 25	15 07461	-2 375
2						
2				0 12	9 670089	-1 125
2	17 7002	18 705		0 875	7 693361	0 875
1						
2				-2 375	16 02464	
1				0 5	13 45927	0 75
2		22 73511	23 76728	0 125	11 69884	-0 5
2				1 625	10 66667	
1	18 70226	19 80561		2	8 755647	1 875
2				0 375	13 59069	0 375
2	20 564	21 577	22 59548	-0 935	10 55989	-2 125
1				-0 065	12 1013	
2				-1 125	15 4935	-1
1						
1		20 58043	21 6345	0 19	9 560575	0
2				-4 5	13 72758	-4 5
1				-0 25	10 2642	
1				0 625	13 54141	0 75
2				0 995	10 32717	0 875
2		20 13963		-2 375	9 106091	-2 875
2	17 69199	18 73785	19 72348	-0 875	7 679671	-1 875
1		20 45175	21 44832	-0 375	9 404517	-1
2	18 46407	19 49897		0 625	8 572211	-0 375
1				-1 75	14 1848	-2
1	19 06366	20 04928	21 08419	1 5	9 007529	1 25
2	18 97604	19 98083		-0 125	8 922656	-1
1	18 0178	19 08829	20 10951	0 56	8 043806	
2				1 5	7 890486	1 5
1		24 09583		0 5	13 08145	0 25
1				-1 125	13 87269	
2		24 8679	25 9165	3 81	13 82615	3 875
2	18 66393	19 61944	20 69268	1 12	8 643394	0 875
1				1	12 47365	1 25
1				0 31	12 75565	1 125

1				-0 18	15 64408	-0 25
1				-2 185	9 22382	-3
1		22 45038	23 48528	0 435	11 42779	0 125
1				0 875	16 11499	1
2	18 79261	19 84121	20 88159	1	8 84052	0 875
1				-2 31	14 91307	-3
2	20 20534	21 21561	22 29432	-0 125	10 23135	-1 25
1				1	10 37919	0 875
2				1 875	8 793977	1 375
2	23 58385	24 61875	25 65366	-1 25	13 57426	-1 5
1		21 36345		-1 87	10 35455	-2 375
2	17 73032	18 74333		0 495	7 715263	0 5
1				-0 125	15 97536	0 125
2	25 56605	26 62286	27 65229	-4 995	15 61396	-4 375
2				0 87	10 69131	1 5
1	22 03422	22 97604	24 03559	0 19	11 96988	0 5
1				3 12	9 97399	2 25
1	18 16564	19 21971	20 23545	0 815	8 186173	0
1				0 5	14 76249	0
2				-1 245	9 511293	
1				0 56	10 2642	0 125
1						
2				0 56	13 1718	-0 125
2						
2			21 2731	-1 245	9 190965	-2 25
1				0 12	11 01164	0 125
2				0 94	9 401779	0 75
2						
2				-2 75	15 65229	-2 75
2				-8 5	13 5551	-8 75
1				-3 5	13 89186	-3 875
1		20 94182		-0 305	9 93566	-0 625
2				0 62	10 42847	-0 25
2		24 84873	25 87817	-2 055	13 83436	-2 5
1				1 685	11 13484	1 375
1				-3 995	12 6872	
1				0 75	14 6256	0 75
1		18 80082		4 19	7 756331	4 375
1				-0 125	10 66393	-0 25
1				-0 555	13 20739	-1 375
1				0 875	7 824778	0 625
2				0 5	9 826146	0 25
1				0 56	16 67351	0 5
2				0 81	10 17933	0 75
1		20 02738	21 06229	1 125	9 002053	1
2				-1 68	16 11773	
2			21 0705	1 125	9 048596	1
1	21 60438	22 68309	23 69336	-1 055	11 6742	-1 875
2				0 435	11 64682	0 125
2				-3 37	12 27378	-3 875
2				-1 375	13 65366	-2
2		25 68925	26 71321	-4 305	14 65845	-5
2	18 38193	19 436	20 47639	-0 125	8 473648	-1
1	23 7399		25 85353	-1 935	13 79055	-2

1				-6 25	14 83094	-6 625
1				0	12 67625	0
1	19 57016	20 56126	21 58795	-0 805	9 563313	-1 5
2				-0 125	16 32307	-0 125
2				-1 745	13 08419	-2 25
2				-9 93	15 44969	-10 375
2	20 05202	21 14442	22 17933	1 5	10 17933	1 25
1	20 48186	21 49487		-0 81	10 48323	-1 25
2	17 92197	18 95414	20 00274	-0 62	7 975359	-2 625
1						
2				0 25	9 960301	-1
2				-0 19	14 0835	-0 25
1				1 12	9 032169	1
2	18 71869			1 185	8 668036	0 75
2				-1 625	16 47091	-2 625
2				1	12 75291	1 25
1						
1						
2				0 94	11 56194	0 75
1	18 05065			0 245	8 041068	-1
2				1 375	9 100616	1 25
1		23 66872	24 68446	0 25	12 70637	-0 5
1	17 86721	18 94319	19 9781	0 56	7 942505	0 25
2			26 48049	-1 87	14 43395	-2 375
2	20 35044	21 38261	22 41752	0 245	10 36003	0 125
1				1 125	15 718	1 125
2			27 86584	-4 375	15 75633	
2						
1				1 375	8 80219	
2				-0 5	13 03491	-1 375
1	21 71663	22 71869		-0 685	11 74264	-1 75
2				1 375	9 79603	1
1	18 56537		20 57769	-0 75	8 550308	-1 875
1		21 99863		1 19	11 01711	0 75
2	18 91307			2 875	8 903491	2 5
2				-2 055	14 47228	-2 125
1				-0 19	13 79329	-0 25
2	23 63039		25 73306	-2 625	13 76044	-2 5
2		22 69678	23 69336	-0 375	11 73169	-1 625
1		23 62218	24 65435	-2 43	12 6078	-3 125
2		26 77344	27 80835	-4 625	15 80287	-4 875
2	25 86721	26 90212		-0 5	15 92882	-0 75
1				0 685	15 8768	0 75
2			21 00205	-0 25	8 991102	-1 375
2	18 60096	19 67693	20 69268	1	8 714579	1
1				1 065	10 10814	0 625
1	21 89459	22 9295	23 96441	0 75	11 94798	0 875
2	22 78439	23 78371	24 83778	0 25	12 81862	-0 125
2	22 41752			0 44	12 47365	
1				1 19	10 40657	1 25
2	19 7755			-3	9 812457	-4
2		21 62354		-0 625	10 65845	-1 5
2				-1 875	10 3217	-3
2				0 185	9 226557	-0 375

2						0 875
2	17 79603	18 84463	19 83573	1 56	7 819302	1 75
1	20 22724	21 16632	22 23409	-0 125	10 32991	-0 5
1				-2 375	15 77823	-2 5
2	21 65366	22 65024	23 6961	-0 625	11 63313	-1 125
1	20 30664			0 75	10 29432	0 5
1				-7 555	14 66119	-7 75
2	26 0616	27 08008		-3 31	16 0575	-3 875
2	25 16632	26 19302	27 22519	-0 875	15 24162	-1 125
2		23 7974		-1 13	12 79945	-2
1				0 935	10 81999	1
2			27 90965	0 5	15 86585	0 25
1	19 48528		21 60164	0 185	9 555099	0 375
1				0 625	9 010267	0 25
2	22 33539		24 42437	-3 125	12 42163	-3 375
2				0 815	8 334018	
1				0 94	8 572211	0 75
1				0 315	9 259412	0 125
2				-0 75	15 87953	
2				-3 625	15 436	-3 625
1						-1 625
1	18 50513	19 54004	20 61875	-1	8 670774	-2
1				-1 055	16 04107	-0 75
2		23 7755	24 82683	-2 75	12 82135	-2 875
1			28 282	-2 995	16 21903	-3 125
1	19 82478		21 83162	0 44	9 776865	0 375
1						-2 875
2				-0 25	14 07255	-0 5
1	18 59001	19 58658		1 69	8 588638	1 125
2						
2				-0 305	10 61191	-2 375
2	18 97331			-0 81	8 974675	-2 375
2				-2 06	9 93566	-2 75
1	17 11978			2 125	7 112936	1 75
2				0	8 750171	-0 5
2	19 99726	21 0486	22 07255	0 185	10 04791	-0 5
1	18 28063	19 33744	20 35866	1 185	8 342232	0 875
1	18 49966			0 37	8 596851	-0 125
1				3 19	9 141684	2 75
1	17 82341	18 85832	19 87132	1 12	7 874059	1
2		20 63244	21 6564	0 565	9 623546	0 75
2	22 25325	23 27995		-0 935	12 25462	-1 875
1	16 73922	17 78782	18 81725	0 94	6 800821	0 625
1				0 995	10 8063	1
2	18 07803			0 995	8 175222	0 375
1	21 74127			1 06	11 81383	1
2	17 65092	18 6475	19 69062	1 315	7 704312	0 875
1		19 84942		-1 995	8 881588	-2 375
1				-0 875	9 399042	-1 75
1				0 3100001	12 17796	0 25
1	19 89049		21 92197	-0 745	9 919233	-1 25
1				1 685	7 225188	1 5
1	19 27721	20 27105	21 32512	0 5600001	9 32512	-0 25
2		22 24504		0 75	11 2553	0 5

2	17 44011	18 53525	19 52909	2 745	7 523614	3
2				-2 5	14 12183	-2 875
1				1 19	7 200548	0 75
2	21 87817	22 96509	23 97262	-4 43	11 97262	-5
1				-0 93	10 80904	-1 125
2			24 12047	-0 38	12 08761	-1
2			20 39425	2 685	8 238193	2 25
1			19 29911	1 06	7 293634	1
1				1 12	10 23682	0 875
2				-5 125	12 7666	-6 25
1				1	8 659822	0 75
2				0 125	11 12389	-0 25
2	17 80424	18 79535	19 85216	1 25	7 857632	0 875
1	20 85695	21 82341		-1 75	10 92676	-2 5
2				1 375	7 646817	1 5
2				-0 5	13 92745	-0 25
2	22 31622		24 42163	-3	12 41889	
2				1 75	6 614647	1 375
1						
1	18 76523	19 80835	20 85969	1 25	8 84052	1 125
1				-3 5	11 77276	-4 375
1						
2	19 07734	20 13689	21 17728	0 75	9 155373	0 75
1		20 93087		0 75	9 949349	-0 5
2				-4 125	13 94114	-4 875
2	25 20739			-4 375	15 3128	-5
1				1 5	8 681725	1 25
2				-0 75	13 40452	-1 25
1	22 42026	23 43327	24 47091	-1 25	12 45448	-1 875
2				0 75	8 257358	0 5
1				-1 5	11 88775	-1 75
1	16 71458	17 75222	18 76523	0 375	6 795346	-1 25
1				-0 75	14 59274	-0 375
1				-2	10 55168	-2 5
1				0 5	10 68857	0 25
1				-0 125	10 58453	-0 625
2	18 54894			0 75	8 558521	0 125
1	19 32375	20 39425		0 5	9 440109	0 375
2	23 34292			0 375	13 40452	0 25
2	22 78713	23 82478	24 8679	1 125	12 87337	0 25
2	22 11636	23 14305	24 14511	-2 75	12 2026	-3 875
1				0 875	7 991786	0 5
2				-0 5	14 34634	-1
1				0 875	8 265572	-0 5
1				2	15 03901	2 25
1	17 85353	18 83368	19 91239	1	7 934291	0 875
2			20 45175	-2 625	8 427105	-4 25
2		27 01437	28 03012	-4 875	16 06571	-5
2	18 14374	19 2334	20 27378	1	8 290213	1 5
1			26 1191	0	14 14374	-0 625
1				-3 055	16 52293	-3 875
2				1 625	12 60507	
1		23 92334	24 95825	0	12 97194	0
2	21 27036	22 26694	23 31554	0	11 33196	-0 875

2	18 25325	19 25257	20 26831	1 81	8 309377	
1				1 19	16 08487	1 5
2	23 88501	24 91718	25 95756	-5 495	13 97947	-5 5
2				-1 805	8 804928	-2 5
1				-1 055	11 81383	-2 25
2	17 85353	18 86379	19 98357	-9 375	7 96167	-9 625
1						
2	16 92266	18 0397	19 03354	-0 375	7 077344	0 125
1	21 82615	22 81999	23 90144	-1 5	11 9206	
1	18 47228	19 52088	20 56947	-0 125	8 626967	-1 375
2		20 17522	21 22108	-0 25	9 240247	-0 75
1		18 68036	19 6961	0 875	7 753593	0 125
1	18 53525	19 54004	20 6078	-0 75	8 632443	-1 75
2				-3 875	13 87269	-4 75
1	17 56605	18 60096		1	7 685147	-0 25
2				0 5	9 705681	0 25
1				1 25	8 191649	1 25
2				0 75	11 03901	
2				0 75	11 78645	1 125
2	20 36687			0 75	10 45311	0
1						
1				2 125	7 895962	1 875
2				-2 25	13 66461	-3 125
1				3 25	15 52088	3
1				0 625	12 2245	0 5
2	21 99863	23 08829	24 08487	2 625	12 15332	2 25
2	21 44011			1	11 54825	0 75
2	19 86037	20 89528	21 96578	-2 125	9 987679	-3
1				0 75	14 76523	1 125
2	19 6167	20 68994		0 75	9 776865	1
1		18 57632	19 63587	1 75	7 665982	1 75
2				1 25	7 312799	0 5
1	17 83162	18 86927	19 92608	0 75	7 950719	0 125
1	17 10609		19 20876	-0 375	7 225188	-0 875
2				-3 5	11 79192	-4 375
1	17 34428			0 875	7 438741	0 5
1				0 125	11 38946	-0 25
1				1 25	9 577003	0 75
2	18 54073			0 75	8 583162	0 625
2	18 98426	20 02464	21 05681	3 375	9 117043	2 25
2	16 84052	17 859	18 9076	-2 375	6 954141	-3 25
1				1 25	14 71047	1
2	16 90075	17 90828	18 95688	0 625	7 022587	0 625
2	22 24504		24 31211	-2	12 37782	-2 75
1				1 125	9 831622	0 875
1				-2 25	14 41478	-2 5
1	17 7796	18 78439	19 82478	1 125	7 863108	
2	22 46133	23 50719	24 53114	-3 25	12 60507	-3 625
1				1 375	9 69473	1 25
2	19 5729			-2	9 713895	-2 75
1						
2				1 25	8 711842	0 875
2				-1 125	14 31348	-1 625
2				0 25	14 97604	

2						
2	22 35455	23 37577		-2 25	12 44901	
1	24 7885	25 78782	26 83915	-3 25	14 88022	-3 625
1		23 64682	24 69541	-3 375	12 77755	-4
2				-3 5	12 61328	-4 75
1						
1	18 0835	19 09103	20 14237	1 125	8 197125	0 875
1				0 5	9 136209	0 375
1		20 64339		1 25	9 752225	1 5
1				0 625	9 284052	
2	24 5859	25 63724	26 67214	-2 5	14 71595	-2 75
1				0 75	10 68036	1
1				-1 5	14 40931	-2 625
1				-3 375	15 58658	-3
1				-4	10 61191	-4 75
1				0 25	14 45859	0
2				1 125	11 60027	0 875
2				1 25	12 30938	0 625
2	24 14511		26 19849	-1 5	14 27515	
1				-0 625	7 832991	-0 875
1	18 69952		20 82136	0 625	8 873374	-0 125
1						
2				-0 125	12 63792	
1				1 125	11 82478	0 875
1	17 9822	18 97331	20 03012	0 5	8 104038	0
2				0 25	11 68515	-0 5
1	17 59617	18 59274	19 64408	0 75	7 723477	0 75
1						
2				0 25	15 08008	
2	16 99384					-1 625
2	17 30595	18 33813	19 38672			1 75
1						
1		21 65914				1 125
1						1
2	19 64134	20 64613	21 69747			-0 375
1	16 30116	17 29774	18 3436			-0 5
2	15 52635	16 55578	17 58795			0 875
1						0 125
2	18 64476	19 72895	20 76386			0 25
2			24 0794			
2	15 74538	16 82683				1 25
2						0 875
2						-0 25
1	15 86037	16 87885	17 87817			0 25
1		23 81656				-5 125
2		23 10472				0 375
1	16 74196	17 74949	18 80082			0 75
1						1
2						0 375
1	16 03285					1
2			19 58111			-0 25
1						0 75
2						-3
2						-1

1	20 53936	21 59069	22 60917	-1
1				-3 25
1	20 16975	21 24572		-0 5
1			23 90691	
1		22 37646		-1 125
1		23 63587		0
1				-4 25
1				0 25
2		23 66598	24 68994	0 125
1	17 08145	18 12183	19 19781	0 75
1	17 58248	18 58453	19 67146	2 5
1		23 5373		-0 375
2	23 01437			-2 25
2				1 5
1	15 90417	16 95825	17 97673	0 375
1	15 64134	16 65708		0 125
1	16 2601	17 26763	18 30253	0 375
2				-3 125
2	16 65161	17 74127	18 77892	0 625
2				-3 75
1	15 83573	16 86516	17 89186	5 75
1				-2 25
1				0 75
1				-3 25
1				-0 75
2				0 875
2				2 5
1				-12 375
1	15 8987	16 91444	17 94114	0 875
1	17 98768	19 06913		0 25
2	16 04381	17 01848	18 03422	0 875
2	18 05339	19 04996	20 05202	0 25
1				1 5
2	15 78919	16 84599	17 88364	1 375
2				1 25
1				0 125
1		21 11157		-1 75
1				0 25
2	16 74743	17 79329	18 81999	0 875
1				3 875
2	18 76523	19 83299	20 82957	0 625
1	16 63244	17 62081	18 69405	0 75
2	16 282	17 30322		3 875
2	17 16359	18 1191	19 15948	
1				-2
1		24 48186	25 51677	-1 375
1		18 71869		-1 875
2				1 5
2	16 25462		18 36824	1
1				-2
2	22 95688	23 96167	24 99384	
1	16 28747	17 295	18 2998	0 375
1				1
2	17 39357	18 40383	19 45517	0 625

2				-3
2	18 11636	19 17591	20 22998	2 125
2				1 75
2	16 98015	18 05339	19 07734	0 75
1	18 93498	19 90691	20 95825	0 625
1				-3 625
2	17 45927	18 54346	19 53183	-0 75
2	16 43258	17 48392	18 51608	0 875
1	18 81725	19 85216	20 90349	0 75
1	16 33402	17 31143		-1
1				
2	19 11567			-1 125
2	18 12457			1 25
2				-3 125
2	17 99042	19 0089		-3 75
1	16 45996	17 40726	18 47228	1 125
1				-3 875
1	15 28816	16 33402	17 37714	1 25
2	15 18412	16 19986	17 24025	1 25
2	23 07187	24 1232		-5
2	15 5154	16 55031	17 57426	2
2	18 2423	19 33196	20 35044	0 25
1				-0 75
1				0 375
2				-0 375
2				-3 875
1	17 76318	18 80356	19 8193	0 375
2	20 29569	21 32238	22 38741	-4 25
1				1 375
1				-4 625
1	18 01506	19 1102	20 10951	0 125
2	17 90281	18 95962	19 99179	-0 75
2	15 83847	16 82409	17 88364	1
1				1 125
2	22 06708			-1 75
1	16 00548	17 01027	18 03696	0 75
1				0 625
2				-2 25
1				0 75
1	25 25941	26 21218	27 26352	
1	20 61054	21 59343	22 69952	
1		18 72142		
1				
1	17 00753	18 04791		
1				
1				
2				
1				
1				
1	18 94593			
2				
1				
2				
2		22 90486	23 93155	

2			
1	16 40246	17 44285	18 48871
2			
1	20 68994	21 74675	22 81451
1		20 23819	21 31964
1			
1			
1	17 19644		19 26352
2		23 93703	
1			
1	16 29843	17 30322	18 32991
1			
2			
2	15 57563	16 60233	
1			21 21013
2			
2	16 97194	18 02053	19 04175
2	17 51403	18 50787	
2			
1		21 12252	22 16838
1			
1		21 15264	
2			
1			
1	14 12183	15 16769	16 21629
1			
1			
2	23 22793	24 282	25 27858
2	16 64613	17 61259	18 65845
1	16 17249	17 20192	18 2204
2		25 30322	
1		21 11704	22 15195
1			
2			
1			
2			
2			
2	19 54552	20 5859	21 65366
2	16 29843	17 295	18 34908
2			
1			23 28816
2			
2			
1	17 2512	18 28063	19 31828
1			
1		21 5332	
1			22 04517
1			
1	17 13895	18 15743	19 23066
2			
2		18 46133	19 43053
1	18 81999	19 8768	
1	17 38535		19 47433
2			

2			21 78234
2	16 83778		
1	20 17522	21 17454	22 20945
2			
1			
1			
1			
1			
2			
1	17 18001	18 18207	19 24983
2	15 85763	16 90349	17 98768
2	19 75359		21 83162
2	17 41273	18 42847	19 47981
1	15 04997	16 12594	17 12526
1	17 23477		
1	16 62423		
1			
2	15 07461	16 14511	17 23477
2			
2			
2	17 92197		19 9781
2			
1			
1	16 03012		
1		22 34908	23 38946
2	22 07255		
1	20 46817	21 45654	22 46133
2	15 98905		18 0397
2	14 6037	15 68789	16 71732
1	21 13347	22 10267	23 1102
2	16 55578	17 59069	18 59274
1			
2	22 79535	23 89049	
1			
2		18 04244	
1	18 96783	19 96715	
1		22 7269	23 80835
2	14 89117	15 95072	16 96372
1			
2			19 34018
1			18 17659
2	17 78782		19 89049
2	23 17317	24 18891	25 21834
1			
1	19 04996	20 00548	
1			
1	16 36961	17 45927	18 55715
2		16 86516	17 93566
1			18 00685
2		17 12526	18 15195
2	19 96988		
2			
1			22 39288
2	16 54757	17 52772	18 57906

1	17 62354	18 67762	
2			
1			
2			
2			
2			
1			
1	15 24162	16 29843	
2		20 19439	21 26215
1			
2			
1	16 31211	17 32786	18 36003
2	15 76454	16 82957	17 87817
2		20 73922	21 80698
1			
2			
2			
1			
2	18 10541		
2			
1	21 62628	22 58727	
2			
2	21 48392		23 57837
2	23 0308	24 10951	25 15811
1			
1	24 94456	26 03696	27 04449
2	18 4668	19 44695	20 49829
2		20 97194	22 0397
2			
2	17 8809		
2			
2	19 02806	20 02464	21 06502
1			
1			
2			
1			
1			
1			
2			24 14784
2			
1			
2	18 23409		
2	19 78371		
2	19 64956	20 62697	21 69473
2			
1			
2	23 68241		
2	20 66256	21 61807	22 64476
2			
1			
1			
2			
1			
2			

2	17 8371	18 81725	19 86858
2			
2			
2			
1			
2		23 09925	24 13142
2			
2			
2		26 37098	
1	16 28474		
1		23 99726	
1		21 21561	22 25051
1			
2			
1	16 80493	17 80151	18 89665
2			
2			
2			
2	18 86927		
2			
2	17 65914	18 65298	19 70979
2			21 69473
1	17 81246	18 86653	19 88501
2			
2	21 16906	22 21766	23 25804
2			
2	17 50582	18 53799	
2			
2	14 423	15 45791	16 50377
2		21 44011	
2	15 16769	16 18617	17 23477
1			
2	15 4935	16 41615	17 55784
1			
2			
2	17 82888	18 86927	
2	22 36824		
1		20 33676	21 33881
2	23 4579		
2			
2	22 96509	23 98083	25 01027
2	16 35044	17 32786	18 3655
2			
2	14 89391	15 95893	16 99384
1			
2			
1			
1	22 87201	23 84942	24 88433
1			
1			
1			
1			
1			
1			

1			
2			
2			
1			
2			
1			
1			
1			
2			
2			24 8679
2			
2			
2			
2		22 63929	
2			
2			
2			
2			
2			
1			
1			
1		22 97057	
2	22 05065	23 07734	
2	21 9165	22 95962	23 97536
2			
1		22 93224	23 95346
2			
2			
1			
2			
2	21 48392		
2	21 51951		23 60575
2			
2			23 48528
2			23 29911
1			
1	20 9911	22 04791	23 08008
2	21 03217	22 01506	23 04449
2		21 92197	
1	19 76181		
1			
1			
1			
2			
2			
1	19 72348	20 69815	
2			
2			
2			
2			
1			
1			
2	20 15058		
2	19 91786	20 87611	

2	16 19986	17 19644	18 26147
2	18 73511	19 81383	20 81588
1			
2	18 53525		
2	18 51608	19 57016	20 62423
2		20 5065	
1		20 59959	
2			21 54141
1			
2			
2			
1	19 36482		
2			
2	19 2334	20 25462	21 31417
1			
2		20 24367	21 26489
1	19 1321	20 07392	21 15537
1			
2			
1			21 02396
2	18 94867	19 97536	20 99384
2	18 94867	19 96988	20 98563
1	18 89391	19 93155	
1			
2	16 8679	17 859	18 93498
1	18 85284	19 8987	
2		19 87953	20 91171
1			
2	17 76591		
2			
1			
1	17 72485	18 75975	
1			19 66872
1	18 57084	19 57016	
1	18 32444	19 38946	20 41068
1	18 3436	19 37851	
1	18 26147	19 31006	
2	18 20397		
1			
1			
2			
1			
1			
1	18 06708	19 10746	20 14237
1	18 07529		
2		19 08829	20 14511
1			
1			
2			
2	16 91171		18 93224
1	17 9165		19 97262
2			
2			
1	17 57153		

1	17 80424	18 84736	
2	17 81793	18 78713	19 85489
2	16 80219		18 79535
1		17 76318	
1	16 70089		18 75428
1	16 74196	17 76591	
1			
2	16 64613		18 65298
1			
1	16 58864	17 55236	18 60643
2			18 60917
2	16 52841	17 53867	18 58727
1	16 60506		
1			
1			
1			
1			
2			
1			
2			
2	17 43463		
1	17 21287	18 3217	19 31828
1		18 36824	
1			
2			
2			
2			
1	17 12526		19 19233
2			
2	17 18549	18 24504	19 25257
2			
1	17 20465	18 1848	
2	17 12252		
1	17 12252		19 1321
2	17 04586	18 0835	
1			
1	16 9473	17 97399	19 01437
2	17 01848		19 0308
2	17 02122		19 08556
1			
2			
2			
2	16 94182	17 94387	19 00068
1			
2			
1			
2			
2	16 82957	17 83162	18 84189
2	16 83504	17 86721	18 89117
2	15 70157	16 73374	17 87543
2	15 75086		
1	15 80287		17 84805
1	15 6961	16 73374	17 83984
1			

1	16 11773		
2	15 72074	16 77207	17 80698
1	15 63587		
2	15 58932	16 65435	17 6783
2			
2		16 6078	17 69473
2			
2	16 48734		
1	16 45996	17 4538	
1	16 45722		18 52977
2	16 42437	17 44011	18 49144
1	16 37782	17 40726	18 42574
1	16 33402	17 3306	18 45585
1	16 30938	17 29774	18 34086
1	16 36961	17 33881	18 42574
2	16 3039	17 32512	18 37646
2	16 20808		18 29432
2	16 16975		18 17385
1			
1			
1	16 11225	17 13895	18 21218
1		17 0924	18 14374
2			
1	15 97262		
1			
2	15 96167	17 00753	18 0178
2			
2	15 96441	16 96646	18 0397
1			
2	15 93155	16 98289	18 04517
2		16 92539	
2	15 6386	16 69815	17 74127
2		16 93908	18 02601
1			
2			
1	15 83573		
1	15 86037	16 89254	17 89459
1	15 83847	16 84326	17 8371
2			
1			
1	14 78987		16 81314
2	14 69405	15 72622	16 7666
2	14 705		16 7447
1		15 77276	
1			
2			
2	14 61191	15 66598	16 6872
1	14 67762		
2			
1			
1	14 5462	15 62218	16 58864
2	14 51061	15 5729	16 58316
2	14 59001	15 60575	16 66256
2	15 63587	16 62423	17 63176

1			
2			
1	16 5421		
2	15 42505	16 46544	17 52225
1			
1	15 47707	16 55305	17 60438
2	15 5373	16 55031	17 56879
1	15 45517	16 53662	
1	15 35113		17 46475
1			
1			
2	15 30459	16 40794	
2	15 30185	16 37509	17 39357
2	15 2553	16 3039	17 30048
2			17 31143
1	15 21424	16 25736	17 25941
1		16 25736	17 29774
2		16 23272	
1			
1			
1	15 17043	16 23272	17 22656
1			
2	15 12936	16 1807	17 22108
2			
2			
1	15 09103		17 12526
1			
2	15 01437	16 07392	17 09788
2	14 98699		17 13347
2	15 0308	16 08761	
2	15 15948	16 15058	17 23203
1			
2	14 97878	16 03285	
2			
1			
2			
2			
1	14 86653	15 90417	16 96646
2	14 78987	15 85216	16 89254
2	14 79261		16 90897
1			
1	14 75154	15 82204	16 84599
1			
2			
1	14 51335	15 48528	16 53936
2	14 51335	15 54278	16 58316
2	14 57358		
1	14 50513	15 55099	16 59685
1	14 39562	15 41958	16 44901
1			
2	14 35729	15 40041	16 45996
1	14 36003	15 35661	16 45722
2		15 38398	
2	14 28337	15 32375	16 34497

2	14 28884	15 3128	16 35044
2	14 19849	15 24435	
1	14 1848	15 20602	16 26283
2			
1			
1	14 12183	15 20055	16 19439
2	14 18754	15 16769	16 24093
2	14 12183	15 18138	16 21355
1	14 13005	15 07461	
2	13 9822	14 98973	16 03012
2	14 06982	15 04723	16 08487
1	13 96304	15 00342	16 02738
1	13 93566	14 97331	16 00821
1	13 95209	14 98152	
1	13 94387	14 96509	15 98905
1	13 94387	14 96235	15 95619
1	13 93292		15 98357
2	13 90554		15 9425
2			
1			
1	18 29706	19 37303	20 40794
2			
2			
1			
2			
1			
1			
1			
1	14 68583		16 73374
2			
1			
2	15 97262	16 95551	17 97125
2	15 85489	16 88433	17 90007
2			
2			
1	18 07529	19 09651	20 13416
2			
1			16 73922
2			
2			
1			
2			
2	16 25736		18 31075
1	15 80561	16 8898	17 90281
2			
1	14 36277	15 38398	16 40246
2	18 58453	19 65229	20 71184
2	15 58932	16 58316	17 58248
2	14 05886	15 06365	16 1013
2	14 78987		16 76386
2	14 52156	15 57837	16 6078
1			
2		23 53183	24 55578
1	18 6037		

1	13 68378	14 72964	15 75086
1	15 47159	16 44901	17 47296
2			
1	18 16564	19 24709	
2	17 21013	18 22861	19 27447
2			
2			
1			
2			
1	17 67556	18 69131	19 72895
2	13 64819	14 69131	15 73169
1	13 28131	14 34634	15 33744
2	13 48118	14 48871	15 56194
2	13 94935	14 96509	15 98905
1	14 76523		
1	17 76865	18 84736	19 88501
2	14 93224	16	17 02122
1	13 8152	14 8501	15 85489
2			
2	18 0616	19 12663	20 12594
1			
1	15 14305	16 18617	17 1937
1			
2	17 14716	18 18207	19 21697
2	14 3655		16 43806
1			
1	17 81246	18 85284	19 89322
1		21 85079	22 90212
2		14 13826	
2	18 99247	19 98631	21 04312
2	13 22108		
2	14 48049	15 47433	16 50103
2			
2			19 99726
2	18 90486	19 88501	20 92266
2	13 44011		15 46612
1			
2	18 43942		
2	13 13895	14 1629	15 18686
1	16 14784	17 1499	18 16564
1	17 1937	18 27242	19 26899
2	20 05476	21 12252	22 14647
2			
2	12 21629		14 23135
2	15 61944	16 65161	17 67283
2	13 86995	14 87474	15 91239
1	11 52361	12 52567	13 55236
2	17 11431	18 19576	19 17043
1	11 32649	12 34497	13 36345
1			
1	11 36482	12 44353	13 4757
1	12 0575	13 0705	14 11088
1	11 57563	12 57495	13 59617
2	19 85489	20 86242	21 94661

1	15 62765	16 65435	17 60986
1			15 5729
2	13 97673	14 99247	16 00548
2	15 30185	16 34771	17 38535
2	10 87474	11 90417	12 93908
2	12 17522	13 15811	14 22861
2	12 55031	13 54962	14 6037
2	10 9076	11 91786	12 91992
2	18 5024		

age_7	seod_8	age_8	seod_9	age_9	seod_10	age_10
15 22519	-1 125	16 20534	-1 125	17 16085	-1 25	18 19028
13 18275			-0 75	15 17317		
11 88227	-2 5	12 8679	-3 125	13 87269	-2 875	14 96509
11 84668	-0 375	12 79124	-1	13 77687	-1 25	14 84736
13 71116	-3	14 60643	-3 25	15 60301	-3 75	16 65708
13 77687	-3 75	14 77344	-3 5	15 71253	-3 75	16 72827
9 251198	-2 125	10 20945	-3	11 17865	-4 125	12 18344
15 78371	-1 75	16 76934	-1 5	17 76865	-1 25	18 78987
12 63244						
13 16906	1	14 15743				
11 94798	-0 75	12 89254	-1	13 89186	-1 5	14 98152
12 69268	1 375	13 68104				
13 06502	2 125	14 02053	2 25	15 00068	2 125	16 0794
12 85968	1 125	13 84531	0 75	14 83368	1	15 87132
11 3347	0	12 30938	0 5	13 31417	-0 125	14 35729
14 56263	-4 5	15 50171	-4 75	16 49829	-5	17 58248
13 40726						
10 45311	0 875	11 43326	0 375	12 44079	0 25	13 51951
	-1 25	12 67625	-2 5	13 59617	-3	14 60643
12 57769	-1 25	13 59617	-2	14 56537	-3	15 56194
12 72553	0 5	13 72211	0 75	14 74333	0 625	15 75633
13 19918	0 75	14 23409	0 25	15 16222	0 5	16 21903
14 93498	-3 25	15 41958	-3	16 3833	-2 75	17 37714
15 9781	-4 5	16 99658	-4 875	17 97673	-5 125	18 96509
	-3 875	13 68652			-3 75	15 61396
13 99042	-3 75	14 97057	-4	15 95346	-4 75	17 04586
10 97604	-0 25	11 95072	-0 5	12 95277	-1	13 94935
8 829569	-1 375	9 817933	-2 125	10 86105	-2 5	11 85489
	-0 75	12 79398	-1 5	13 72211	-2	14 75428
12 1013	0 25	13 0705	0 875	14 07529	0 75	15 11567
15 86311	-1 25	16 88433	-0 75	17 85353	-1	18 8501
10 77344	0 625	11 73443	0 625	12 69268	1	13 74675
15 52361	-2 5	16 53114	-2 75	17 49487	-3	18 55441
12 32854			-3	14 23682	-3 5	15 25804
11 68241	0 25	12 65708	-0 25	13 69199	-1 125	14 71047
17 07324	-1	18 09719	-0 875	19 0527	-0 75	20 07118
13 78508	-5	14 76523	-5 5	15 75633	-5 5	16 76386
11 29637	0 375	12 27926	0 75	13 27584	0 25	14 40383
14 01506	-1 5	14 93498	-1 75	15 93977	-2 25	16 95277
13 27858	-1 375	14 29979	-1 375	15 27721	-1 125	16 3039
15 07187	-5 625	16 09583	-6	17 0705	-6 5	18 06981
13 13895						
14 62012	0 375	15 67967	-0 25	16 61875	-0 125	17 65366
9 741273	0 25	10 62286	-0 25	11 65503	-0 875	12 76934
12 96372	-3 5	13 97947	-4 25	14 94319	-4 25	15 96715
16 36413	-2 75	17 34155	-2 875	18 3655	-3 5	19 35661
16 0575	-1 375	17 06502	-1 625	18 07529	-1 25	19 03901
8 741958	0	9 691992	-1 75	10 70773	-3	11 74812
12 80219	1 5	13 80972	1	14 78439	0 75	15 79192

16 09035	0 75	17 11978	0 5	18 09719	0 625	19 08282
9 582478	-0 75	10 5462	-1 75	11 64134	-2 75	12 56126
9 782341	-0 75	10 74059	-1 375	11 72074	-1 875	12 77755
11 73443	-1	12 67899	-1	13 65914	-0 625	14 68036
8 769336	1	9 735797	0 75	10 73238	0 75	11 75359
11 51814	-6 125	12 50924	-7	13 50582	-6 875	14 55989
11 14305	-0 375	12 25188	-0 375	13 13621	-0 375	14 14647
9 075975	0	10 09993	-0 875	11 04723	-1 5	12 07118
13 01574	-1	14 00684	-1 375	14 99521	-1 375	16 0219
14 25599	-3	15 21697	-4 125	16 17249	-3 875	17 26489
16 58316	-8	17 58248	-8 75	18 56263	-9 5	19 57563
9 921971	-1 125	10 94593			-2 25	12 95551
14 87474	-1	15 84394	-1 5	16 83778		
14 46954	-0 25	15 46612	-0 75	16 45448		
10 09719	-1 25	11 07187	-1 5	12 04928	-2	13 18275
13 19644			0	15 25257	0 25	16 26831
9 275839	-3 75	10 27515	-4 25	11 26899	-5 125	12 27105
8 859685	1 25	9 859001	1	10 85558	0 75	11 93155
12 75291			-1 75	14 75975	-2 375	15 718
15 82204	-3 25	16 84873	-3	17 91923	-3 125	18 86653
14 80082	-0 5	15 79466	-0 875	16 78576	-1	17 79329
15 29911	-1 25	16 31211	-1 125	17 28405		
16 70637	-3 125	17 74401	-3	18 65572	-3 375	19 72074
12 41615	-3 5	13 40178	-3 5	14 35181	-3 5	15 44695
11 67146	0	12 67077	-0 5	13 64271	-0 25	14 68036
8 974675	0 875	9 913757	0 375	10 87201	-0 125	11 90691
12 15058	-1 625	13 1499	-1 75	14 11636	-2 25	15 14305
11 09651	-4 25	12 10678	-4 625	13 19918	-5 125	14 09993
					-2	12 87337
9 938398	0 125	10 86653	-0 25	11 8768	-0 5	12 9117
17 12526			-3 875	19 05544	-3 75	20 09309
	-1 75	10 87201	-2 5	11 8412	-3 25	12 87337
15 11294	1 25	16 10678	1 25	17 11157		
15 16222	0 5	16 19165	0 375	17 14168	0 375	18 14647
15 89596	-2 5	16 90349	-3 375	17 85353	-3 25	18 89665
12 75565	-2 625	13 79055	-2 75	14 73511	-3 125	15 81656
12 86242						
17 2512	-3 25	18 24504	-4 125	19 1896		
16 97467	-5 125	17 93566	-5 375	18 93224	-5 375	20
13 34702	-0 5	14 3217	-0 75	15 27447		
	-6 25	15 96988	-6	16 87337	-6 25	17 89186
13 55784	-2 25	14 55168	-2 375	15 51266	-2 625	16 61054
13 53593	-1	14 53525	-1 5	15 45791	-1 25	16 50924
14 23682	-2 25	15 24162				
10 51335	1	11 47707			0 75	13 51129
9 809719	-5 5	10 75428	-7 125	11 76181	-8 125	12 75291
10 4449	-0 5	11 44969	-0 625	12 42984	-2 5	13 46201

10 17659	1 25	11 17591	1	12 14784	1	13 18275
9 555099	-4 625	10 58453	-5 25	11 55921	-6 125	12 53114
16 5859						
8 709104	1 75	9 724846	1 75	10 69952	1 5	11 6742
11 2909	-1 25	12 29295	-1 5	13 25941	-1 625	14 3436
14 95962	-3 375	15 96167	-3 25	16 92266	-3 25	17 97673
15 718	-4 5	16 7447	-5 25	17 70294	-5 375	18 7269
13 50856	-2	14 45585	-2 625	15 41684	-3	16 42437
16 36961	-0 5	17 37166	-0 375	18 3217		
	1 5	12 21903	1 25	13 20192	1	14 32444
14 51882					0 75	17 56058
14 14374	0 125	15 1321	-0 25	16 07118	-0 25	17 10335
	0 5	15 8193	0 5	16 81862	0 5	17 82341
12 26283	-0 75	13 27036	-0 75	14 22861	-0 75	15 24435
8 966461	0 875	9 954825	0 875	10 95414	0 75	11 95893
12 87885	-0 875	13 81793	-1 875	14 75154	-1 875	15 80835
			-0 625	12 71458	-0 75	13 81246
12 15606	-3 5	13 14442	-4	14 11636	-5	15 13758
15 26899	-1 625	16 30938	-2 125	17 23203	-1 75	18 24778
11 43874	-0 5	12 44901	-1 125	13 50034	-1 375	14 46407
16 79945	-5 625	17 79877	-6	18 77892	-5 875	19 80835
13 31417	-0 5	14 26694	-0 5	15 24162	-0 25	16 28747
16 21082						
15 83847	0 5	16 84052	0 25	17 83162	0 25	18 85284
11 98083	5 125	12 98289	5 25	13 94935	5 625	14 96235
10 2642	0	11 27173	-0 75	12 26831	-1 25	13 29774
12 62423	-3 125	13 10335	-4	14 17933	-4 125	15 10472
17 54689						
8 632443	1 125	9 645449	0 375	10 62286	0 75	11 59754
8 91718	-0 5	9 908282			-1 75	11 92334
	0 25	14 73511	0 375	15 71253		
8 687201	1	9 672827	0 875	10 63929	1	11 68515
15 03354	-0 25	15 98357	-0 25	16 9692	0	18 04244
15 73443	-6 5	16 73922	-6 5	17 6783	-6 625	18 78713
12 3258	-7	13 30322	-7	14 27242	-7 875	15 27995
9 963039	-1 25	10 47228	-2	11 42505	-2 625	12 45175
9 89733	-1	10 85558	-2 375	11 80561	-3	12 82957
12 54757	0 5	13 52498	0 375	14 48597	0 375	15 54004
16 38604						
14 98973	0 75	15 99452	0 625	17 01574	0 75	17 99315
13 6564	0 75	14 63655	0 5	15 57563	0 25	16 62697
16 49281	-5 625	17 49213	-6 25	18 47775	-6 5	19 54004
10 36277	0	11 35661	-0 5	12 3258	-1 25	13 32238
9 954825	0 625	10 93498	0 375	11 90965	0 75	12 94456
14 20123	0 625	15 1321	0 625	16 11499	0 875	17 25941
9 412731	-1 25	10 41478	-2	11 36208	-2 625	12 41068
9 574265	0 5	10 55989	0 125	11 52361	-0 375	12 53114
10 94593	0 5	11 84668	0 375	12 86516	0 5	13 87543
9 886379	0 125	10 8501	-0 375	11 86037	-0 375	12 85695
11 48255	-0 125	12 46817	0	13 44285	-0 125	14 45311

10 71321	1 75	11 70705	1 75	12 75017	1 25	13 68925
12 35592	-1	13 34155	-1 875	14 30801	-1 875	15 32923
14 52704	-2 5	15 51266	-3 25	16 5065	-3 75	17 50034
13 51129	-2 75	14 44764	-3 5	15 41136	-3 625	16 41615
	-1 5	15 55373			-2	17 51129
14 34086	-3 875	15 3128	-4 5	16 2601	-4 75	17 32786
9 440109	0 875	10 4011	0 875	11 38125	0 875	12 41342
13 61533	-2 125	14 60643	-2 875	15 56742	-3 75	16 61875
	-5	17 7796	-5 375	18 72142	-5 75	19 83025
14 01232	0 5	14 99795	0	15 94524	-0 75	17 00205
10 13005	-2 375	11 08282	-3 25	12 0575	-4	13 06776
11 93429						
9 442847	-0 125	10 48049				
12 97741	-0 5	13 97125				
10 94593	-1 125	11 9206	-1 5	12 90897	-1 875	13 91376
15 93703						
16 89254	-1 25	17 89733			-1 25	19 87953
12 61054	0 375	13 53867	0 25	14 48871	0 25	15 62491
13 16906	-0 875	14 12731	-1 5	15 16222	-1	16 1013
10 6475	-3 5	11 62218	-4 25	12 65982	-4 75	13 63723
					-1 25	13 19918
11 30459	-3 75	12 282	-5 5	13 2731	-5 625	14 34634
	-6 25	16 72279				
12 99384	-5 25	13 97125	-6	14 94593	-5 875	16 00274
9 667351	1 25	10 65024	0 5	11 58932	-0 125	12 72279
11 04997	-1	12 04107	-1 5	13 01027	-1 25	14 04791
	-0 5	11 3922	-0 75	12 38877	-0 625	13 44285
	-2 375	15 88227	-2 875	16 81862	-3 625	17 94935
14 90486	0 25	15 88227	-0 25	16 96646	0	17 99315
10 78166	0 375	11 70705				
11 0308	1 25	11 98905	1	12 97468	1 25	14 0835
12 1013	-2 5	13 06776	-2 75	14 02875	-2 625	15 09377
14 15195	-1 25	15 13484	-2	16 0794	-2 5	17 11978
8 741958	0 375	9 71937	-0 25	10 705	-0 625	11 71526
11 63587	-0 375	12 61328	0	13 59343	-0 125	14 64203
9 957563	-1 25	10 93498	-2 625	11 89596	-3 75	12 97468
16 0794	-3 625	17 0705	-3 75	18 05613	-4	19 03354
15 90965	-2 5	16 91444				
	-0 25	13 02396			-0 125	15 08556
15 10472	-2	16 07118	-2 25	17 00479	-2 625	18 03422
12 62971	-3 25	13 60986	-3 75	14 58179	-4 25	15 58111
11 7974	0 875	12 77481	-0 375	13 74401	-0 875	14 84189
9 336071	-1 75	10 32991	-2 75	11 27447	-3	12 40794
12 68172	-3 5	13 66188	-4 25	14 60643	-4	15 718
9 28679	0 375	10 20671				
10 48049	-1	11 49076	-1 25	12 44901	-2	13 50034
15 03901	-7 875	16 05476	-9	17 00753	-8 75	18 10267
9 144422	0 875	10 10541	0 375	11 09925	0	12 09856
9 281314	1	10 25599	0 75	11 23888	-0 25	12 23546
17 46749			1	19 3922	0 5	20 42984
16 88433	-1 375	17 82341				
10 1848	-2 375	11 16222	-3	12 09309	-3	13 14442
14 91855	-2 5	15 87406			-3	18 01232

8 829569	0 75	9 856263	0 25	10 80082	0	11 79466
14 84736	-2 25	15 75086	-2 75	16 72553	-2 625	17 77139
15 90965	-1 75	16 89802	-2	17 9165		
9 544147	-0 75	10 5024	-1 375	11 46064	-2 25	12 51745
	-0 875	10 92676	-1 25	11 88227	-1 5	12 92266
10 39288	-1 375	11 37029	-3 25	12 35318	-4 25	13 35797
13 64819	-6 75	14 6256	-7	15 58111	-6 75	16 61875
11 8412	-1 625	12 84326	-1 75	13 82341	-2 25	14 83368
12 94456	-2 5	13 92197	-0 75	14 87474	-0 75	15 90144
10 98152	-1 875	11 95893	-2 5	12 93087	-3	13 97125
10 97331	-2 375	11 96441	-2 75	12 93908	-2 875	13 9822
12 53662	-3	13 50308	-3 75	14 47502	-4	15 50171
9 127995	-0 125	10 20397	-1	11 06913	-1 375	12 09583
	-3 75	11 59206	-4 125	12 57495	-4 25	13 5989
14 94867	-6 875	15 93703	-6 875	16 92266	-7 125	17 91102
10 3217	1	11 27447	0 5	12 2601	0 5	13 2731
11 15674	-3	12 10678	-4 25	13 02669	-4 25	14 0616
16 10951	-3	17 09514	-3 625	18 05065	-3 25	19 06366
10 69952	-2 25	11 69884	-3	12 64887	-2 75	13 79329
8 725531	0 375	9 672827	0 25	10 69952	0	11 68241
					-2 125	15 6961
	-3	18 09719	-2 75	19 09103	-2 75	20 09583
14 5024	0 875	15 40862	0 625	16 38877	0 625	17 38535
12 72005	-0 625	13 74127	-1 625	14 72964	-1 875	15 78097
	1 125	12 67899				
9 79603	1 5	10 76797	1 375	11 72622	0 75	12 75017
14 62834						
11 58932	-3	12 56674	-3 5	13 53593	-3 875	14 58453
	0 375	14 10541				
16 52567	-1 5	17 50034	-1 75	18 46133	-2 25	19 60575
10 65845	-0 5	11 55099	-1 25	12 55305	-1 75	13 55784
14 76249	-5	15 74264	-5 125	16 73648	-5	17 75222
	-1 875	12 29843	-2 5	13 23203		
14 52977	0 25	15 56468			0 5	17 60712
11 36208						
10 14647	-3 625	11 0883	-3 75	12 10404	-4	13 10062
8 717317	-2 875	9 661876	-3 25	10 65298	-4	11 70979
10 44764	-2 125	11 42779	-3	12 40246	-3 5	13 39904
9 475701	-0 75	10 45859	-1 25	11 43053	-1 5	12 51472
15 22245	-2 5	16 19986	-3 25	17 21287	-3 875	18 1848
10 0835	1 25	11 07187	1 125	12 00821	1 5	13 08966
9 938398	-1 25	10 94045	-1 375	11 88775	-1 375	12 94456
			-0 75	11 01437		
8 928131	1 375	9 883641	1 5	10 87474		
14 12183	0	15 10198	0 25	16 0575	0 25	17 08693
	-1 375	15 88775	-1 625	16 90075	-1 5	17 93566
14 83368	3 875	15 84942	4	16 79671	4	17 84257
9 661876	1 25	10 61465	0 375	11 62491	0 75	12 6078
13 44285	0 75	14 41752	0 375	15 50992		
13 80972						

16 69541	-0 375	17 65914	-0 625	18 61191	-0 625	19 6386
10 25873	-3 375	11 2115	-4 125	12 21629	-4 875	13 21287
12 45722	-0 375	13 43463	-1 25	14 4011	-2 5	15 41136
17 08966	0 5	18 05065	0 75	19 00342	1	20 06297
9 894592	0 25	10 83094	0 125	11 83847	0	12 82135
15 93703	-3 25	16 91718				
11 25804	-2 5	12 2245	-3	13 21013	-3 75	14 24778
11 42231	0 625	12 36961	0 25	13 35797	0 5	14 39836
9 853525	1 5	10 78439				
14 61191	-1 75	15 58658			-2 375	17 57974
11 38946	-2 5	12 34223	-3	13 32786	-2 875	14 39014
8 750171	-0 25	9 702943				
17 02943	0 25	17 99042				
16 64887	-4 875	17 63176	-4 875	18 58727	-4 75	19 64408
11 78371	1 125	12 69541	0 75	13 66461		
12 99932	0 25	13 9603	-0 5	14 97057	-0 375	16 03833
11 01711	1 25	11 9781	1 125	12 96099	1	14 01506
9 229295	-1 5	10 19576	-2 75	11 16222	-3 625	12 18891
15 78371	0 25	16 75565	0 5	17 72211	0	18 76797
	-3 125	11 54825	-4	12 56674	-4 125	13 577
11 30185	0 125	12 29843				
14 21492	-0 625	15 18138			-0 625	17 21287
10 25325	-3 625	11 19233	-4	12 15332		
12 05476	-0 375	13 04586	-0 25	13 98494	-0 125	15 08282
10 45859	0 875	11 37851	0 125	12 35318	0 25	13 37166
16 61054	-3 125	17 58795	-3	18 55989		
14 50787	-9 125	15 5729	-9 375	16 46544	-9	17 53867
14 89938	-4 5	15 88501	-4 625	16 86516	-4 75	17 859
10 94319	-1 25	11 88501	-1 25	12 8898	-1 25	13 92471
11 45243	-0 75	12 44079	-1	13 43463	-1 25	14 41478
14 85284	-3	15 78919	-3 25	16 87064	-3 875	17 84257
12 21903	1 375	13 14442	1 375	14 0835	1	15 154
	-5	14 76249	-5 125	15 6386	-5 125	16 77481
15 62218	0 625	16 58864	0 875	17 58522		
8 788501	4 125	9 73306	4	10 73785	3 625	11 78645
11 72348	-1 125	12 64613	-1 5	13 61533	-2	14 60643
14 23956	-1 25	15 2115	-1 75	16 18891	-1 75	17 22656
8 837782	0 375	9 752225	-0 125	10 76249	-1	11 8193
10 80356	0 5	11 78097	0 125	12 75838	0 25	13 83436
17 71116	0 375	18 65024			0 5	20 67625
11 21424	0 375	12 1807	-0 5	13 13347	-0 5	14 26694
10 03149	0 875	10 99795	0 75	11 98631	0 5	12 98563
			-2 25	19 09103		
10 06434	0 75	11 05818	0 5	12 01095	0 5	13 01848
12 7091	-2 5	13 66461	-2 5	14 63655	-2 875	15 66872
12 72827	-0 75	13 64819	-0 625	14 63655	-0 875	15 66051
13 295	-3 875	14 27515	-4 25	15 23888		
14 68583	-1 75	15 66051	-1 875	16 6078	-1 625	17 65366
15 67967	-5 5	16 65161	-5 5	17 64271	-5 5	18 64476
9 467488	-1 875	10 43669	-2 75	11 41684	-3 5	12 44901
14 8282	-2 875	15 86858	-3 125	16 84873	-3 125	17 76865

15 82204	-7	16 77481	-7 25	17 73306	-7 375	18 76523
13 70842	-0 375	14 77892	-0 5	15 65777		
10 59548	-1 875	11 55099	-2 25	12 55852	-2 375	13 53046
17 28953	-0 625	18 24504	-0 375	19 24435	-0 625	20 25736
14 1191	-2 75	15 07187	-3 5	16 06845	-4	17 05407
16 45722	-10 375	17 4538	-10 75	18 38193		
11 15127	1	12 18891	0 75	13 06502	0 875	14 13552
11 51814	-1 75	12 49829	-1 625	13 45106	-2	14 45859
9 004791	-3 625	9 968514	-4 375	10 95962	-4 75	11 9781
10 88022			-2 75	12 80767	-3	13 91923
15 16222	-1	16 0794	-1 5	17 03217		
10 07255	1 25	11 04997	0 625	11 99179	0 875	13 08145
9 754962	-0 5	10 65571	-2 5	11 7399	-3 25	12 65435
17 49213	-2 25	18 4668	-2 625	19 46338		
13 72485	1	14 68036	0 875	15 66324	0 5	16 67899
12 59411	0 25	13 57426			0 25	15 58932
9 007529	-1 5	10 00137	-2 75	11 01437	-3 5	12 04654
10 08624	0 75	11 01711	0 5	11 99452	0 125	13 01027
13 6783	-0 875	14 6475	-1 875	15 60301	-1 875	16 62149
8 966461	-1 375	9 91102	-1 875	10 92676	-2 25	11 90965
15 44148	-2 625	16 42984	-2 875	17 41547	-3 5	18 39288
11 38398	-0 25	12 3258	-1	13 32512	-1 25	14 33812
16 71458	1 25	17 65366	1 25	18 63929	1 25	19 67693
			-5 25	18 75154	-5 125	19 76455
	1	10 82546				
14 03422	-1 25	15 03628	-1 875	15 9425	-1 875	17 00479
12 77755	-2 375	13 7358	-2 5	14 69952	-3 125	15 71526
10 84736						
9 571527	-2 75	10 49966	-3 75	11 52088	-3 375	12 52841
12 03833	0 25	12 97468	-0 5	13 95756	-0 75	14 97604
9 924709	2 25	10 86105	1 625	11 83847	1 375	12 87611
15 51814	-2 125	16 47912	-1 75	17 46201	-1 875	18 4668
14 75702	-0 375	15 79466	-0 5	16 73922	-1	17 76318
14 7269	-2 625	15 66598	-3 25	16 70089	-3 375	17 66461
12 72279	-3 375	13 63176	-4	14 62834	-4 375	15 66872
13 62628	-3 625	14 56263	-3 625	15 55647	-3 75	16 59411
16 79398	-5	17 74675	-5 5	18 74333	-5 5	19 79192
16 90897	-1	17 86721	-1 625	18 83368	-1 375	19 86584
16 82409	0 25	17 79877	0 5	18 78439	0 375	19 81109
9 960301	-2 25	10 89117	-2 625	11 90144	-3 5	12 91444
9 691992	0 375	10 62012	1	11 62765	0 25	12 62423
11 12936	0 75	12 08487	0 25	13 05407	-0 375	14 09719
12 93635	0 625	13 90828	0 25	14 85558	-0 125	15 9425
13 85353	-0 125	14 74333	-0 75	15 72895	-0 375	16 7885
					-0 375	16 49008
11 4141						
10 78439	-4 75	11 74264	-5 5	12 72553	-5 5	13 73032
11 62765	-2 375	12 63244	-3 375	13 56605	-3 875	14 59822
11 31006	-3 625	12 26283	-4 125	13 25941	-4 625	14 25873
10 25051	-1 5	11 20602	-2 25	12 19165	-2 75	13 25667

13 29227	0 25	13 78508	-0 75	14 75975	-1 5	15 7755
8 84052	1 25	9 806981	0 75	10 80082	0 75	11 77823
11 34565	-1	12 30116	-1	13 37714	-1 625	14 3436
16 75017	-2 25	17 7577				
12 66256	-1 875	13 58248	-1 75	14 59001	-1 75	15 57837
11 32375	0 25	12 22177	0 125	13 21834	0 25	14 27242
15 6961	-7 25	16 66256	-7 125	17 63997	-7 375	18 69678
17 06502	-4 375	18 06708	-4 75	19 1102	-4 5	20 00274
16 22177	-2 625	17 19918	-3 375	18 17112	-3 75	19 16496
13 81793	-2	14 77071	-2 875	15 7399	-3 375	16 80219
11 83847	0 5	12 79671	0 625	13 76044	0 625	14 81451
16 88433	-0 5	17 87269	-0 875	18 83094	-1	19 85216
10 57358	0 375	11 5373	0 375	12 51472	0 625	13 51403
9 968514	0	10 96235	-0 625	11 97262	-1	12 97194
13 43737	-3 875	14 4011	-4 125	15 37577	-4 375	16 38877
	0 5	10 38741				
9 604381	0 375	10 5243	0 125	11 54278	-0 375	12 53936
10 32444						
					-0 5	19 80835
16 4052	-3 625	17 38809	-3 875	18 31622	-4 125	19 35387
17 87543	-2	18 4449				
9 535934	-2 5	10 52977	-2 875	11 51814	-3 875	12 564
17 06502	-1 5	18 05065				
13 83436	-4	14 71595	-4 75	15 71253	-3 875	16 80767
17 25941	-4	18 21492	-4 625	19 18686	-4 5	20 25188
10 84189	-0 75	11 72348	-1 5	12 73922	-2 25	13 7358
14 62286	-2 875	14 62286				
15 0883	-0 75	16 05476	-0 75	17 1499		
9 604381	0 5	10 53799	0	11 55099	-0 875	12 55305
11 59206	-3	12 5859	-3 5	13 58795	-4 125	14 59822
9 990417	-3 25	10 92402	-4 25	11 97536	-5 125	12 95277
10 95688	-4 375	11 90417	-5 375	12 87064	-6 125	13 89186
8 128679	2 25	9 108829	1 625	10 05065		
9 776865	-1 75	10 68857	-2 25	11 72895	-2 75	12 73374
11 06092	-1 125	11 99452	-1 75	12 97468	-2	13 98768
9 355236	0 75	10 28884	1	11 32923	0 75	12 30664
9 626284	-1 75	10 5462	-2 5	11 55099		
10 14647	2 125	11 07187	1 75	12 10678	1 5	13 09514
8 84052	0 5	9 812457	-0 625	10 83368	-1 125	11 81383
10 63381	-0 25	11 58932	-0 25	12 55305	-0 75	13 60986
13 23477	-2 75	14 22313	-3 375	15 17865	-2 875	16 25188
7 750855	0	8 741958	-0 5	9 727584	-1 75	10 73238
11 81656	0 875	12 7666	0 375	13 76865	0 5	14 85558
9 18549	-0 875	10 13826	-1 625	11 10746	-2	12 14511
12 83778	0 5	13 77413	0	14 77892	-0 75	15 74538
8 681725	1	9 659138	0 625	10 63929	0 125	11 69336
9 861738	-3 75	10 87748	-2 875	11 80561	-2 75	12 85147
10 39014	-2 625	11 33744	-3 5	12 38877	-3 875	13 38809
13 1937	-0 25	14 14921	-0 75	15 14853	-0 375	16 18617
10 93224	-1 875	11 8768	-2 5	12 88433	-2 875	13 90281
8 210814	1 5	9 158111	1	10 19028	1 125	11 15948
10 30253	-1 25	11 26626	-1 5	12 29021	-2 375	13 25941
12 26557	0	13 18549	-0 375	14 21766	-1 125	15 24162

8 531143	2 5	9 456536	2 25	10 48871	2 125	11 49897
15 12936	-3 625	16 09856	-3 625	17 08693	-4	18 07529
8 208076	-0 25	9 182752	-2 75	10 1629	-4	11 18138
13 02396	-5 25	13 94114	-6 25	14 93498	-6 25	15 94798
11 81656	-1 375	12 74196	-2	13 73853	-1 875	14 75154
13 10335	-1 25	14 02053	-1 875	15 05544	-1 5	16 06297
9 24846	1 5	10 17112	0 5	11 20602		
8 30664	0 75	9 226557	0 75	10 31348	0 5	11 30185
11 24435	-0 25	12 17248	0 5	13 15811	0 375	14 17659
13 76044	-5 875	14 75702	-6	15 71526	-5 875	16 78029
9 6564	0 125	10 63107	-0 25	11 57837	-0 625	12 64066
12 16975	-0 5	13 06229	-0 5	14 0397	-0 5	15 06913
8 865161	0 5	9 793292	0 25	10 81725	-0 875	11 8193
11 93155	-3 625	12 85968	-4	13 86995	-5 25	14 97604
8 651608	1 375	9 582478				
14 93224	-0 5	15 8768	-0 5	16 85969		
	-4 375	14 37645	-5 25	15 3347	-6 125	16 38056
7 619439	1 75	8 572211	1	9 546885		
9 842573	0 25	10 81451	0 125	11 7974	0	12 83231
12 77755						
10 15743	0 25	11 10198	-0 5	12 18891	-1 5	13 13073
10 9514	-1 5	11 89596	-2 75	12 87337	-3 375	13 92745
14 98426			-5 625	16 92266	-5 75	17 8809
16 32307	-5 25	17 27584	-5 5	18 28611	-5 875	19 24162
9 672827	0 875	10 63929	1	11 63313	0 75	12 68994
14 40931	-1 375	15 37851	-1 125	16 35592		
13 48665	-2 625	14 40657	-3	15 36482	-3 25	16 45996
9 24846			-0 375	11 16769	-0 375	12 22177
12 82957	-2 25	13 79877	-2 25	14 87748	-2 25	15 85489
7 709788	-2 25	8 703628	-2 875	9 69473	-3 5	10 75428
15 58385	-0 625	16 53936	-0 875	17 53593		
11 52361	-3 125	12 48734	-3 75	13 46749	-4 25	14 4668
11 70431	-0 5	12 65161	-0 625	13 65092	-0 375	14 70226
11 55373	-1 25	12 5065	-1 125	13 47844	-1 25	14 49966
9 54141	-1 375	10 47502	-1 5	11 56194	-2 5	12 57495
10 42574	0 375	11 35387	0	12 34771	0	13 34429
14 41478	0 06	15 32923	-0 5	16 42163	-0 25	17 31964
13 81246	-0 25	14 80356	-0 625	15 7974	-0 25	16 79124
13 13895	-4 625	14 09172	-5 375	15 1321	-5 5	16 10404
8 892539	-0 25	9 886379	-1 25	10 88843	-1 75	11 9206
15 31006	-1 375	16 25736	-1 125	17 26489	-0 875	18 24778
9 275839	-1 625	10 19028				
16 01643			1 75	18 05339		
8 898015	0 75	9 823409	0 5	10 84189	-0 5	11 87132
9 379877	-5 625	10 31622			-6 75	12 37782
17 02669	-5 375	17 95209	-5	18 95688	-5	19 98083
9 264887	1	10 17659	0 875	11 2115	0 875	12 17796
15 11567	-0 75	16 03012	-0 375	17 06229	-0 25	18 07255
17 43737	-4 25	18 43395	-4 75	19 40589	-5 375	20 46817
13 88912	-0 125	14 85832	0 25	15 9425	-0 75	16 90349
12 24367	-1 375	13 25394	-2 25	14 23409	-3 25	15 25257

17 05133	1 5	18 00958				
14 94867	-5 875	15 86311	-5 625	16 87611	-5 625	17 88912
9 774127	-3 75	10 72964	-3 75	11 73169	-3 875	12 75017
12 77755	-3	13 73853	-3 375	14 71595	-3 625	15 72348
8 966461	-10 25	9 850787	-11	10 94867	-11 5	11 89049
8 07666	-0 25	8 958248	-2	9 993155	-3 375	11 01164
	-3	13 80972	-3 625	14 80082	-4 25	15 82752
9 612594	-1 875	10 56263	-2 5	11 50445	-3	12 51472
10 14921	-1 75	11 16496	-2 75	12 13963	-3	13 18001
8 709104	-1 125	9 629022	-2 125	10 66119	-3 25	11 70157
9 623546	-3	10 53525	-4 25	11 52909	-5 5	12 53388
14 86105	-5	15 74812	-5	16 78029	-5 125	17 7577
8 572211	-2	9 557837	-3	10 59001	-3 875	11 55921
10 69952	-0 5	11 65777	-1	12 61875	-1 75	13 6345
9 14716	1	10 06708				
	0 125	12 94456	-0 125	13 97399	-0 125	15 00342
12 74743	0 625	13 66188	0 25	14 69405	0 5	15 66324
11 37577	-0 875	12 32854	-1 875	13 32512	-1 75	14 41752
8 843258	1 875	9 768652	2 125	10 76797		
14 66393	-3 5	15 58658	-3 25	16 55305		
16 45722	3 125	17 42916	3 25	18 39014	3 25	19 40315
13 17728	0 125	14 141	-0 5	15 10746	-0 25	16 11773
13 10883	2	14 02327	1 875	15 05818	2 125	16 07666
12 46544	0 25	13 46475	-0 625	14 43943	-1	15 47433
10 96783	-4	11 88227	-4 125	12 89254	-4 875	13 88364
15 71253	1	16 63518	1	17 65092	0 875	18 65024
10 72416	0 375	11 64682	0 25	12 68446	-0 375	13 66461
8 61054	1 25	9 535934	1	10 55168		
8 246407						
8 876112	0 625	9 817933	0	10 83368	0 375	11 83847
8 106776	-1 875	9 242984	-2 75	10 21218	-3 25	11 11567
12 71732	-5	13 68925	-5 375	14 69405		
8 388775	-1 125	9 305955	-2 125	10 30253	-3 125	11 33196
12 32033	-1 75	13 28405	-3 375	14 26968	-3 375	15 26899
10 50787	0 5	11 45243	0 625	12 55578	-0 125	13 59069
9 533196	0	10 45311	0	11 44969	-0 25	12 50376
10 04517	2	11 02259	1 25	12 03012	1	13 06229
7 898699	-4 375	8 818618	-5 5	9 850787	-7 375	10 85832
15 65777			0 75	17 59343		
7 950719	0 25	9 023956	-0 5	9 900068	-1	10 91307
13 30595	-3 75	14 24504	-4	15 27721	-4 25	16 25736
10 78439	0 125	11 74538	-0 75	12 70363	-1 75	13 69747
15 33196	-3 125	16 31211				
			0 75	10 83641	1	11 87132
13 5551	-4 5	14 55715	-5 25	15 46064	-5 5	16 49829
10 57632	1 125	11 55647	0 75	12 55578	0 875	13 70568
10 63929	-3 625	11 60301	-4	12 6078	-4 625	13 5989
9 667351			-0 5	11 57563		
15 23614	-1 75	16 23272	-1 5	17 1718	-1	18 26147
	-0 125	16 83231	0 125	17 86721		

			-2 5	15 38672	-2	16 35044
15 75359	-4 125	16 79124	-4 25	17 76318	-3 75	18 78987
13 69747	-4 5	14 63655	-4 75	15 66872	-5	16 63792
13 51677	-5 5	14 47228	-5 875	15 50445	-6 375	16 49281
9 114305	1	10 09172	0 5	11 0883	0 5	12 06023
10 05339	0	11 0308	-0 125	12 03012		
10 7269	1 25	11 61123	1	12 64613	1 25	13 62355
	0 25	11 17865				
15 63587	-3 5	16 57495	-3 5	17 57153	-3 625	18 57084
11 61944						
15 33196	-3	16 26831	-3 5	17 30048	-4 125	18 33265
16 51745	-3	17 51129	-3 125	18 44216	-3 375	19 46064
11 59206	-5 125	12 52841	-5 625	13 45654	-6 25	14 49144
15 36208	0 5	16 39425	0	17 34702	-0 125	18 31622
12 57769			0 75	14 57632	0 5	15 54004
13 19918	0 25	14 17659	0 25	15 24983	0 125	16 31485
	-2 125	16 18344			-2 5	18 26694
8 810404	-2 25	9 727584	-3 25	10 705	-3 875	11 74264
9 806981	-0 75	10 76797			-2	12 74196
	-0 5	14 49966	-1	15 57837	-0 875	16 50377
12 79945						
9 032169	-0 75	9 965776	-2	10 98973	-3	12 01095
12 62971	-1 5	13 59343				
8 651608	0 75	9 585216	0 75	10 57358	1	11 63313
	0 75	16 98289	0 625	18 04517		
7 953456	-2 875	8 969199	-3 75	9 971252	-4 25	10 98973
8 323067	1 5	9 314168	0 125	10 27242	0	11 32923
11 72348	0 875	12 62697	0 875	13 63176	0 625	14 66393
9 037645	1	9 037645				
11 17317	-0 625	11 69062	-1 125	12 64339	-1 625	13 64271
7 791924	-0 875	8 320329	-1 5	9 259412	-2	10 30527
7 047228	1	7 581109	0 25	8 550308	0 5	9 577003
13 58248	0 125	13 58248	0 25	15 06092	0 25	16 15058
10 18754	0 25	10 72416	0	11 67967	-0 25	12 6653
	0 875	14 06708	1 25	14 99247	1 25	16 02464
7 293634	1 375	7 835729	0 875	8 796715	0 625	9 790554
7 797399	0 875	7 797399				
11 30185	-0 25	11 30185	-1 5	12 77207		
7 348392	-0 5	7 857632	-1	8 826831	-1 25	9 867214
14 29432	-5 125	14 78713	-5 375	15 73443	-6 75	16 77207
13 58248	0	14 10541	-0 875	15 03901	-1	16 05476
8 271048	0 5	8 807666	-0 5	9 730322	-1 25	10 78166
13 67009	1	14 20671	0 25	15 16496	0 875	16 17249
13 18275	0 375	13 18275				
7 493497	1 25	8 032854				
9 040383	-0 875	9 568789				
8 197125	0 75	8 197125	0 875	9 629022		
13 4757	-3	13 99316	-3 875	14 93498	-4	15 98631
12 30664	-1 625	12 83231	-2 25	13 7796	-2 5	14 80356

12 08761	-1 5	12 62423	-2 125	13 54415	-3	14 55989
12 4052	-2 875	12 93361	-3 625	13 89186	-3 375	14 89391
11 7974	-0 375	12 29295	-0 75	13 1937	-1	14 2204
	3	13 88912	3	14 83915	2 875	15 87132
12 84326	-1 125	12 84326	-1 75	14 30801	-2	15 32375
14 12183	-0 25	14 65298	-1 125	15 57837	-0 25	16 59959
12 41889	-4 25	12 41889				
12 35318	-0 125	12 87064	0	13 80424	-0 5	14 8501
14 13826	0 125	14 13826	-0 125	15 58932	0 25	16 6078
8 668036	0 75	9 14716	0 5	10 13005	-0 5	11 16496
9 09514	1 75	9 626284	0 875	10 64203	0 125	11 58111
14 02327	-0 25	14 54073	-0 5	15 47159	-0 25	16 58043
14 53525	-2 25	15 06365	-2	15 99452	-2 5	17 03217
7 854894	1 5	7 854894				
7 433265	0 5	7 964408	0 25	8 919918	-0 75	9 941136
7 170431	0 125	7 170431			-2 5	9 708419
7 77002	0 25	8 29295	-0 5	9 226557	-1	10 24778
11 13484	-3 25	11 66051	-3 875	12 61875	-4	13 60986
8 219028	0 625	8 73922	-1	9 678303	-1 5	10 69678
13 96304	-3 75	13 96304				
7 446954	5 625	7 917864	5 125	8 862423	4	9 861738
13 44011	-2 375	13 95756	-3	14 91034	-3 375	15 92882
13 28405	0 75	13 28405	0 75	14 74059	0 75	15 81656
14 5681	-3 375	15 0883	-4	16 04654	-4 625	17 05955
13 51129	-1	14 04517	-1 25	14 97878	-1	15 99179
11 76454	1 125	12 31211				
8 481862	2 5	8 481862	1 75	9 954825	1 125	11 0089
11 83573	-11 75	12 35044	-12 25	13 29227	-12 5	14 28884
7 422314	0 625	7 950719				
9 557837	0 25	9 557837	-1 125	11 0308	-2	12 03012
7 531827	0 875	7 531827	0 5	9 004791		
9 514031	0 25	9 514031	-0 625	10 98973	-1 25	12 00274
8 262834	1 5	8 262834				
7 345654	1 375	7 863108	1	8 818618	0 5	9 798768
12 03559	1 25	12 55031	1	13 49213	1 25	14 56263
12 71184	0 125	12 71184				
11 58932	-1 625	12 08487	-1 875	13 05681	-2 5	14 09993
7 805613	-0 625	8 320329	-1 125	9 278576	-1 625	10 29432
8 303902	0 875	8 303902				
8 454483	3 875	8 454483				
10 30801	0	10 8063	0 75	11 79192	-0 25	12 78029
8 142368	0 75	8 142368				
7 778234	4	8 295688	3 875	9 267625	4	10 28884
	1	9 106091	0 75	10 09172	0 5	11 14579
14 23409	-2 375	14 74333	-2 625	15 68515	-2 5	16 77755
14 97878	-1 875	15 46338	-2	16 44353	-2 25	17 44559
9 218344	-1 875	9 218344	-3 25	10 71047	-4 5	11 72348
12 25188	1 25	12 76386			1 125	14 81177
7 77002	0 875	8 265572	0 375	9 234771	0	10 2423
8 17796	-2 75	8 703628				
7 767282	0 125	8 298426	-0 375	9 262149	-1 25	10 23682
12 57221	1	13 12526	1	14 0616	1 25	15 12936
8 919918	0 125	9 429158	-0 875	10 40931	-1 5	11 47159

14 33539	-3	14 83915	-3 625	15 78371	-3 25	16 84052
9 653662	2 125	10 15743	1 625	11 12936	1 5	12 14511
11 1896	1 75	11 69336	1 25	12 69815		
8 503764	0 875	9 007529			0 5	11 0527
10 40657	0 375	10 9295	0 25	11 88775	-0 75	12 89254
12 16427	-3 75	12 68172	-4 625	13 62902	-5 125	14 63107
9 013005	-1 25	9 519507	-2 25	10 48871	-3 625	11 47433
7 947981	1	8 454483	0 5	9 478439	-0 25	10 46133
10 35455	0 5	10 89117	-0 375	11 83025	-0 625	12 82957
7 830253	-1 5	8 358659	-1 625	9 316906	-2 5	10 33539
			-4	15 06913	-4 75	16 21082
10 55441	-1 25	11 07187	-1 875	12	-1 75	13 05681
9 620808	1 375	10 13826	1 125	11 10472	0 625	12 08761
12 00821	-3 75	12 52841	-4 75	13 48392	-5	14 4668
9 505818	-4 5	10 02601	-6 25	10 98973	-7 25	11 99179
7 901437	1 375	8 429843	1 125	9 357974	1 125	10 40383
9 61807	-4 375	10 21218				
6 86653	1 25	7 37577	1 375	8 361396	1 125	9 347023
6 732375	1 25	7 307324	0 75	8 194387	0 25	9 204655
14 62012	-5 375	15 11294	-6 125	16 05202	-6 375	17 11431
7 093771	1 75	7 572895	1 25	8 54757	0 625	9 519507
9 856263	-0 25	10 31622	-0 25	11 29363	-0 875	12 29021
16 71184	-0 625	17 18549			-0 375	19 15674
16 15606	0 625	16 64066	0 25	17 6345	0 375	18 72142
12 34497	-0 875	12 78029	-1 375	13 7358	-1 375	14 75975
8 465435	-3 875	8 465435				
9 355236	0 375	9 355236	-0 75	10 77344		
11 86585	-4 5	12 33402	-4 875	13 295	-5	14 30253
7 759069	1 375	7 759069			0 25	10 17933
17 21287	-4 625	17 21287	-4 75	18 61191	-4 5	19 68789
9 590692	-0 25	10 06434	-0 5	11 03901	-1	12 0575
9 505818	-0 75	9 505818	-3 5	10 91034	-3 75	11 89596
7 400411	0 75	7 835729	0 875	8 81588	1	9 831622
12 29021	1 125	12 77755	1 375	13 72211		
13 59069	-1 75	14 06434	-2 5	15 01164	-2 125	16 08214
7 542779	0 625	8 019165				
13 31964	0 5	13 80151			0 5	15 75907
16 44353	-2 875	16 90623	-2 75	17 87543	-2 75	19 00342
15 52635	1	16 04928	0 75	16 94182	0 875	18 03696
	-5	17 24846	-5 5	18 23409	-5 75	19 20876
	-0 375	12 6653	-1 375	13 56879	-2 25	14 56537
	0 875	9 746749	0 125	10 65298	0 25	11 67693
			1	16 9692		
	-2 75	9 070499	-3 375	10 01232	-3 875	11 03354
	-0 625	16 72827	-0 625	17 66735		
	-1 375	17 14716	-1 75	18 05065	-1 5	19 07187
			-1 25	11 01711	-3	12 02738
	-6 75	17 71663	-6 75	17 71663		
			-0 125	12 06571		
	0	16 33402	-0 25	17 27584	-0 5	18 28884
			0 375	16 64066		
			-0 25	14 88569	-0 25	15 9206

0 875	7 854894	1	8 747434		
-2 75	8 492813	-3 75	9 431896	-4 125	10 42026
-0 25	12 81588	-1 25	13 71663	-1 375	14 71321
-1 5	11 3128	-2 625	12 2026	-3 625	13 22656
-0 875	13 91923	-1 75	14 80356	-1 875	15 83299
-4 75	16 6078	-5	17 58522	-6	18 56263
		0	10 19302	0 25	11 21424
-0 25	15 02259	-0 625	15 89322	-0 875	16 95825
1 25	11 27995			1 125	13 24298
		0 5	9 259412	-0 25	10 28337
-1 5	17 41821	-1 5	17 41821	-1	18 97604
		1 125	8 525667	1 5	9 587954
		4 5	12 12594	4 75	13 1718
		-2	9 971252	-2 875	11 05544
		1	10 49144	0 75	11 50992
		-0 25	13 05407	-0 5	14 15195
-2 625	12 17522	-3 75	13 07871	-4 375	14 11362
-6 5	12 18344	-6 5	12 18344	-7 75	13 6783
-3	12 62697	-3 125	13 09788	-3 625	14 14921
		-5 125	17 00753	-4 875	18 02875
		-2 375	15 72622		
		0 125	7 118412	-0 125	8 114989
1 875	17 1937	1 625	18 10267	1 375	19 10472
-1 625	12 23819	-1 75	13 11978		
		-5 875	16 23272	-6 25	17 25393
		-0 125	9 577003	-1 75	10 57906
		1 25	9 160849	1 375	10 19028
		-1 125	17 295	-1	18 28884
		0	13 05133	-0 25	14 07529
		0 625	9 59343		
1	17 18001	0 75	18 09719	1	19 11841
-3 75	15 39493	-4 25	16 26283	-4 375	17 32512
3 75	14 65845	3 75	15 55373	4	16 59685
0 375	11 64408	-0 125	12 564	-0 125	13 58795
		0 5	9 284052	0	10 30527
-0 375	11 95346	-0 625	12 87337	-1 75	13 86721
-2 25	13 30048	-3 125	14 1848	-3 25	15 23888
		-2	15 91786	-2 5	16 91444
		-0 125	15 05818		
		-2 875	10 22587	-4 125	11 27995
		0 75	8 720055	0 625	9 724846
-1 125	12 65982	-1 875	13 52772	-1 625	14 54346
		0 5	12 95277	0 5	13 98768
-3 25	14 71321	-4	15 63587	-4	16 66256
1 125	9 253936			1 25	11 22793
0 375	9 453798	-0 125	10 38741	-0 25	11 39767
		1 25	11 81109	1	12 85421
1 375	9 415469	1 5	10 33539	1	11 33196
		-1 375	17 94935	-1 375	19 07187

		0 75	12 64887	0 875	13 66188
		-2 875	9 79603	-3 5	10 81451
		-4 25	13 1499	-4 875	14 22313
		-3	13 75496	-4	14 76523
-1 5	8 681725	-2 25	9 188228	-3 625	10 23956
		1	11 23614		
1 25	8 314853	1 125	9 38809	1 25	10 34634
-1 75	12 21903	-2 75	13 18001	-3	14 31622
-3 5	13 11157	-4	13 56605		
-1 625	9 207392	-3 375	10 19028	-4 25	11 18138
0 25	7 887748	-0 5	8 865161	-1 5	9 902806
0	12 27652	0	12 77207	-0 25	13 78234
		0 125	10 39562	-0 5	11 50445
0	7 151266	-0 875	8 095825	-3 75	9 207392
		-0 25	10 13552	-1 625	11 17043
-0 125	8 651608	-1	9 626284		
0 125	11 20055			-0 75	13 25667
-1 25	7 137577	-1 625	8 095825		
1	7 709788	1	8 698152	0 75	9 713895
-0 5	15 17865	-1	16 1807	-0 75	17 1499
		0 125	10 92402	-0 25	12 01643
		0 75	8 684463	0 5	9 681041
0 625	13 78782	0 625	14 72416	0 5	15 77823
-0 25	8 010951	-1 75	8 971937	-3	9 949349
0 5	13 3306	-0 75	14 26968	-2 25	15 34018
		-1 125	15 12115		
		0 625	13 40178	0 625	14 43943
		-1 375	9 004791		
-0 125	6 67488	-0 125	7 652293	-1 125	8 668036
-4 5	13 15537	-4 375	14 02053	-4 375	15 07461
0 75	8 553046			0 375	10 58727
		-0 25	9 251198		
-0 125	14 87201	-0 375	15 80835	-0 5	16 86242
-1 625	14 02053	-2 5	14 96783	-2 375	16 02738
		0 25	9 93566	-0 25	11 01437
		1 625	11 8987	1 125	12 9117
0 125	13 74127	-0 75	14 70226	-1 375	15 71253
1 375	6 954141	0 875	7 92334	0 75	8 966461
4	14 84463	3	15 78371	3 75	16 93634
		-1 125	10 19576	-1 875	11 1896
				-5 125	10 26147
		-1 625	10 81725	-3 125	11 84394
-3	15 15674	-3 125	16 09583	-3 125	17 16359
1 625	11 04997	1 5	11 98905	0 625	12 99658
-7 555	11 52088	-8 25	12 50103	-8 75	13 50308
1	8 438056	1 25	9 423682	0 75	10 43943
		0 75	8 807666	0 75	9 908282
1 125	7 947981	0 625	8 91718		
0 625	8 106776	0 25	9 070499	0 5	10 08898
-2 25	11 97262	-2 5	12 94182	-2 625	13 94935
		-1 625	13 28953	-2	14 32717
		0 75	9 497604		

		-4 25	10 68309	-5 25	11 63587
-0 375	11 91239	-1 125	12 8898	-1 875	13 91102
0 125	11 99179	0 25	12 93635		
		-0 75	9 251198	-1 375	10 31348
-2 75	11 75086	-3 75	12 71732	-4 5	13 80151
1	7 753593	0 875	8 736482	0 75	9 744011
		0 5	8 273785		
		-3 375	12 14784	-3 625	13 16906
		0 5	12 564	-0 25	13 66461
				-3 375	14 52704
0 875	8 320329	0 75	9 289528	1	10 36277
		3	8 807666	2 625	9 826146
1	11 75086	0 625	12 77755	0 375	13 82067
-5 125	12 41615	-5 75	13 36893	-5 875	14 44764
-3 75	12 49008	-4 125	13 44832	-4 625	14 48049
		-1 625	12 68172	-1 5	13 73306
		-4 125	15 87132	-4 625	16 95277
0 125	10 12731	-0 125	11 09103	-0 125	12 15606
		3	9 566051	2	10 61465
-2 25	14 39288	-2 5	14 89665	-2 75	15 89322
		-2	14 49144	-2	15 49076
-2 375	15 17043	-2 75	16 04107	-3 125	17 07598
		-2	12 29295		
		-2 125	17 96578	-1 75	19 07187
		-2 75	11 44148	-3 125	12 47912
-1 75	12 47912	-1 75	12 47912	-2 875	14 05065
-0 75	18 34086	-0 5	18 78987	-0 75	19 81656
		-0 375	10 9076	-1 125	11 91513
-3 625	11 0883	-4 125	11 98631	-4 625	13 02396
0 125	12 30938	-0 875	13 24846	-1 25	14 3436
-1 25	13 70842	-2	14 58453	-2 25	15 60575
		-0 875	15 90691	-0 5	16 93087
0 75	17 47844	0 375	17 96578	0 375	19 06913
-0 25	12 37235	-0 75	12 84052	-1 125	13 93292
-9 625	16 74743	-9 625	16 74743	-9 5	18 23409
		-0 25	15 07187	-0 25	16 12047
		-4	11 78097	-4 75	12 80219
-0 75	11 8412	-1 625	12 75565	-2 875	13 77139
		0 5	12 67625	-0 125	13 61259
-5 375	18 21218	-5 375	18 21218		
1	12 39151	-0 875	13 32238	-2	14 43669
		-2 75	16 73101	-3 125	17 71937
0 25	12 65708	-0 375	13 56605	-0 75	14 57906
		-2 75	18 78439		
		0 25	10 89117		
		-2 75	14 48323	-3 375	15 49897
		-2	19 22793	-1 75	20 25462
		-0 5	11 54004	-1 125	12 61328
-3 25	17 45106	-3 75	17 93292		

-3	9 875428	-4 25	10 79808	-4 875	11 84942
		-5 875	17 06776		
		5 125	16 53936	5	17 63176
-2 25	18 90486	-2 25	18 90486		
				-3 75	16 19165
				-0 5	12 846
		0 25	16 34223	0 5	17 41821
1	8 312115	1	9 300479	1 5	10 30801
		-1 75	15 98905	-2 25	17 02669
0	12 23272	-0 5	13 18549	-1 625	14 20123
-5	15 09651	-5 25	15 98083	-5 875	17 00205
				-9 75	17 82341
		-2 25	9 744011	-3 625	10 78439
		-6 625	13 84805	-6 75	14 88296
		-1	12 6078	-1 125	13 67556
		0 75	9 36345	0 5	10 40931
		-2 75	11 77276	-4 75	12 81588
				-2 875	11 55921
				-3	11 7399
		-1 625	12 5859	-2 25	13 61259
		1 5	10 80082	0 125	11 84394
-3 125	16 03285				
		-1 75	14 20123	-2 25	15 20876
0 125	7 709788	-1	8 629705		
		1 125	10 48871	0 875	11 55647
1 625	6 398357			0	8 377824
-3 25	12 47365	-4	13 42916	-3 875	14 48597
0 625	7 175907	0 5	8 136892	0 625	9 166325
-2 375	10 13552	-2 875	11 09651	-3 75	12 07666
0 75	7 471595	0 75	8 421629	0 5	9 508555
-3 5	14 84189			-5 25	16 83231
-3 75	10 06982	-4 375	11 01437	-5 375	12 04928
-0 375	9 924709	-1 25	10 84189	-2	11 83025
1 25	14 46133	1 125	15 34018	1 375	16 39425
0 375	11 37577	-0 125	12 25188		
-2 5	15 50992	-3 75	16 36961	-3 75	17 38261
-3 5	16 26831	-3 75	17 1499	-3 75	18 14374
-2 25	15 04997	-3 25	15 89596	-4	16 94182
2 5	8 807666	2 375	9 305955	1 75	10 36277
-5 75	7 581109	-6 5	8 090349	-6 875	9 073237
0 75	7 444216	0 5	7 953456	0	8 950034
1	10 04244	1	10 55441	0 75	11 56742
-2 75	15 24435	-3 5	15 72074	-4	16 73648
-2 625	15 2909	-3	15 73717		
-2	15 31554	-2 5	15 76728	-2 25	16 82683
-3	15 16496	-3 125	15 74264	-2 625	16 66804
-0 625	15 11841	-0 875	15 63313		
-2 75	15 09377	-3	15 55373	-3 5	16 56126
-2 75	15 10198	-3 125	15 54552	-2 625	16 58864
-0 25	15 12389	-0 125	15 58658	0	16 59685
-2	15 12115	-2	15 12115		

-0 25	15 95346	-0 25	15 95346	0	17 48939
0 5	15 51266	0 75	16 01095	0 25	17 02122
0 125	15 45517	-0 375	15 95346		
-2 375	15 40041	-2 375	15 40041		
-1 25	15 3922	-1 625	15 89596	-2	16 91718
		-3 75	15 86858	-3 75	16 98563
-2 875	15 36756	-3 25	15 82478	-3 875	16 87064
0 75	15 32923	0 75	15 32923		
-2 125	15 31006	-2	15 77276		
1	15 31006	1	15 80835	1 5	16 82409
-2 375	14 26968	-2 875	14 72416	-3 25	15 84394
		-0 625	14 57084	-0 25	15 65503
-2 5	14 07803	-2 625	14 54346	-3 125	15 56194
0 125	14 11088	0 125	14 56263		
-2 5	14 87748	-2 5	14 87748		
		-1 25	15 20876	-0 625	16 24914
-1 875	14 78439	-1 875	14 78439	-1 875	16 27926
-1 75	14 71595	-1 875	15 16769	-2 125	16 21355
-1 375	14 57906	-2	15 05818	-2 5	16 14511
		-3 25	15 0308	-3 5	16 06845
-2 5	14 45311	-2 75	14 93224	-2 625	15 95346
-2 5	14 53525	-2 375	15 00068	-2 625	16 01917
-2 625	14 42847	-2 625	14 90212	-2 375	15 95346
-5 75	14 44764	-5 75	14 44764		
		0 875	14 86927		
-3 75	14 37098	-3 75	14 37098	-4	15 85763
-0 125	14 33539	-0 5	14 80082	-0 5	15 83847
0 75	13 1937	0 375	13 69473		
0 5	13 08419	0 5	13 54415	-0 375	14 57632
-2 5	13 98494	-2 375	14 4668	-2 25	15 47433
0 5	14 02875	0 75	14 51335	1	15 59754
				-0 25	15 54825
0	13 90554	0	14 38467	-0 5	15 45243
-3	13 70294	-3	13 70294	-3 125	15 21697
-1 125	13 60986	-1 125	13 60986		
-2 375	13 50034	-2 75	13 99316	-2 625	14 99795
		-2 625	13 99042	-3 25	15 00342
-3 75	13 36071	-4 375	13 8371	-4 75	14 85284
-1	12 25462	-1 25	12 72827	-1 75	13 73306
		0 5	12 76386	0	13 69747
0 75	12 27926	0 75	12 27926		
1 875	12 25736	2	12 73648	1 5	13 78508
0 375	12 16701	0 375	12 16701		
-2 25	12 18891	-3	12 68172	-4 5	13 74949
-0 375	12 20808	-0 75	12 67899	-0 5	13 72211
0 375	13 04312	0 25	13 53046		
		-4	12 51198	-4 375	13 56057
-1 875	12 99658	-2	13 48939	-1 75	14 56537
-1 5	12 84052	-1 5	12 84052		
-1 375	12 72005	-1 25	13 19096		
1 25	12 66256	1	13 1499	1 125	14 15469
-6	12 70363	-6 375	13 28131		
-3 375	12 38056	-4	12 87337	-4 5	13 8809

-1 375	8 722793	-2 625	9 204655	-3 125	10 2204
-1	11 29637	-1 75	11 7399	-2 375	12 76112
-2 875	11 13484	-3 375	11 6167	-3 875	12 61054
-4 625	11 07461	-5 625	11 57563	-6	12 60507
-1 75	11 07734	-1 625	11 5729	-2 125	12 64339
1 25	11 98083	1 25	11 98083		
-3	12 04107	-2 75	12 53662	-3 25	13 58795
3 375	11 92334	3	12 41889	2 375	13 43463
-1 25	11 83847	-1 75	12 30116	-2 125	13 37166
0 25	11 83573	0 25	11 83573		
		-4	12 33949	-4 875	13 28953
4 375	11 81109	4 25	12 29021	4 5	13 3306
-3 375	11 75633	-3 875	12 24641	-4 375	13 26762
-1 375	11 74264	-1 625	12 21355	-2	13 23477
-4	11 6386	-4 25	12 12047	-5	13 16359
		-0 25	12 22177	-0 375	13 26489
0 5	11 68241	0 5	12 18891	-0 625	13 18275
		-0 5	12 00274	-1 25	13 08419
-0 875	11 45517	-0 875	11 45517		
0 25	11 45517	-0 375	11 93155	-1	12 98015
0 625	11 436	0 5	11 90691		
-4 25	11 39493	-4 625	11 89322	-4 5	12 92266
0 125	11 44695	0 375	11 93155	0 5	12 92539
0 75	9 393566	0	9 878165	-0 625	10 99247
-1 75	11 37303	-2 25	11 85489	-3 25	12 84326
-1 375	11 35661	-1 375	11 83573	-1 75	12 84052
2 375	10 26968	2 375	10 26968		
0 75	10 24504	0 5	10 74606	-0 125	11 75907
-0 75	10 21766	-1 5	10 69405	-1 5	11 68515
-1 125	10 17385	-1 375	10 64203	-2 5	11 70705
0 875	10 17933	1 125	10 68583	0 625	11 67967
-0 5	10 12183	-0 5	10 12183	-1 375	11 5948
0 5	11 02806	0 5	11 02806	0	12 564
0 125	10 84736	-0 5	11 34292	-1 25	12 32854
-0 75	10 83641	-1 875	11 33744	-3 375	12 33402
0 625	10 77071	0 125	11 2909	-0 25	12 29295
-1 875	10 65024	-2 375	11 15948	-3 125	12 17248
0	10 67762	0	10 67762		
		-6 125	11 18138	-7 5	12 1807
		0	11 35661	-0 5	12 33676
1 75	10 58727	1 75	10 58727		
2 125	10 6037	2 125	10 6037	1 5	12 15332
-3	10 56263	-3 875	11 06365	-5	12 04928
-1 375	10 61465	-1 625	11 05544	-2 5	12 08487
-0 625	10 48049	-0 875	10 98152	-1 5	11 98083
1 75	10 52977	1 25	11 02806	1 25	12 10951
1	10 51608	1	10 51608		
1 125	9 399042	0 375	9 91102		
-0 75	10 38467	-1 75	10 85832	-1 5	11 96441
		0 125	10 86105	-0 875	11 8987
1 625	10 07803	1 125	10 58179	1 125	11 56194

0 875	10 33539	0 875	10 33539	0 25	11 84942
-0 875	10 30253	-1 375	10 78987	-2	11 81383
-1 5	9 251198	-1 75	9 752225	-2 25	10 82272
0 625	9 226557	0 75	9 69473	0 75	10 705
		-0 25	9 69473	-1	10 73785
1 125	9 253936	1	9 738535	1	10 81725
		1	9 566051	0 625	10 61191
1 375	9 075975	1 25	9 582478	1	10 59001
-0 25	9 065024	-0 375	9 560575		
1 125	9 051334	0 875	9 54141	1	10 55989
1	9 051334	1	9 051334	-0 125	10 66393
-2 375	9 114305	-3 125	9 615332	-3 75	10 63929
		-2 125	9 741273		
				0 125	11 60575
1 25	10 0397	1 25	10 0397	0 75	11 54278
		1 25	10 46133	0 875	11 48255
-3 5	9 793292	-3 75	10 27242	-4 5	11 25804
-0 625	9 880903	-1 125	10 37645	-2 125	11 48255
0 625	9 782341	0 625	9 782341		
2 25	9 738535	1 75	10 19302	1 5	11 33744
-0 25	9 670089	-1	10 17112	-3	11 1896
		-0 875	10 14374	-2 125	11 16222
-2 875	9 661876	-3 375	10 11636	-4	11 1321
		0 75	10 19028	0 125	11 27173
		-8 125	10 19028	-9 125	11 20055
-3	9 711157	-3	9 711157	-4	11 22519
1 5	9 61807	1 25	10 08898	1	11 0883
0 75	9 59343	0	10 05339	-0 25	11 10472
-1 375	9 566051	-1 375	9 566051		
0 625	9 524982	0 625	9 524982		
0 75	9 486653	0 75	9 486653		
				0 25	11 08008
		0 75	10 02327	0 875	11 10472
		0 75	9 886379	1 25	10 92676
		0 875	9 886379		
		1 375	9 954825	1	10 98152
		-2	9 93566	-2 75	10 93498
		-2 875	9 823409		
0 875	9 319644	0 875	9 319644	0 625	10 89391
		-1 125	9 787817	-1 875	10 78439
		1 5	9 785079	1 25	10 81451
		0 75	9 823409	0 875	10 91307
		0 875	8 720055	1	9 839836
0 875	8 246407	0 875	8 246407		
				4 625	9 853525
1	8 232718	1	8 232718		
		1 25	8 750171	1 375	9 801506

				1 5	10 19302
1 375	8 276523	1 375	8 774812	1 125	9 752225
				-2 25	9 738535
		0 75	8 632443	0 5	9 702943
				-0 375	9 73306
		1 25	8 580424	1	9 612594
		0 75	9 478439	0 25	10 46954
0 375	8 985626	-0 375	9 467488	-1 5	10 45311
0 125	8 982888	0 25	9 46475	-0 25	10 48049
1	8 930869	0 75	9 415469	0 5	10 39836
		-0 25	9 371663	-1 5	10 40383
		-1	9 295004	-1 875	10 32444
0 75	8 835045	0 75	9 28679	0 75	10 4011
0 5	8 835045	0 5	8 835045		
		1 25	9 344285	0 75	10 3436
		-4 125	9 180014	-5 375	10 19849
		0 75	9 103354	0 375	10 15195
		1 25	9 133471		
		1	9 133471	1	10 15195
		1 375	9 065024		
		0 125	9 054072	-0 375	10 08077
		-3 5	9 078713	-4 25	10 12731
				-2 75	10 07803
		0	8 950034	-0 75	9 971252
		-2	8 960985		
		1	8 996577	0 625	10 03696
		0	8 919918	-0 5	9 954825
		0 125	8 870637	-1 125	9 894592
		1 125	8 643394		
		0 875	8 91718		
		1 625	8 796715	1 125	9 861738
		0 5	8 796715	0 375	9 867214
		0 75	8 862423	0 5	9 869952
		1 25	8 747434	0 875	9 817933
		1 25	8 898015		
		0 75	8 821355		
		2	7 712526	2	8 769336
		0	7 70705	-0 875	8 788501
		0 5	7 693361		
		-0 375	7 709788	-1 375	8 73922
		3 625	7 74538	3 5	8 826831
		1	7 624915	1 125	8 635181
		0 75	7 603012		
		0 75	7 534565	0 75	8 678987
		1 875	7 529089	1 375	8 566735
		1	7 589323	1 125	8 709104
0 875	8 112252	0 875	8 599589	1	9 582478

		0 875	8 438056	1 25	9 478439
		7	9 48939	6 625	10 55441
		-0 25	8 451745	-1 5	9 451061
		-0 875	8 481862	-0 875	9 555099
		0 5	8 492813	0 25	9 566051
		0 875	8 47091	0 75	9 497604
		-0 125	8 347707		
		1 25	8 355921	0 625	9 357974
		0	8 323067	-1	9 385352
		0 875	8 320329	1	9 344285
		0 25	8 224504	0	9 314168
		0 75	8 208076	0 5	9 229295
-0 375	7 750855	-1 5	8 191649	-2 875	9 256674
		0	8 227242	-1 375	9 300479
		0 875	8 186173		
				1 25	9 28679
		0 625	8 136892	0 75	9 232033
		0 875	8 114989	0 625	9 130733
		-0 625	8 145106	-1 125	9 169063
1 25	7 60575	0 875	8 07666	0 875	9 177276
3 25	7 60575	3 375	8 054757	2 75	9 070499
1 5	7 586585	1 5	7 586585	1 125	9 073237
0 625	7 572895	0 375	8 054757	-0 25	9 051334
-0 375	7 56742	-0 75	8 049281	-1 625	9 111567
1 25	7 641342	1	8 087611	1	9 138946
-2	7 529089	-2 75	8 114989	-3 75	9 026694
0 5	7 523614	0 625	8 005476	0 375	9 023956
		0 125	7 964408	-0 875	8 963723
		-0 25	7 890486		
		0 875	7 909651		
		-0 75	7 865845	-1 125	8 922656
		0 75	7 816564	0 625	8 843258
		-0 5	7 800137	-1	8 848734
		-0 875	7 753593	-1 75	8 788501
		-0 625	7 800137	-0 5	8 865161
		1 125	6 699521	1	7 720739
		-1	7 49076	-2	8 574948
		0 625	7 504449	0 25	8 591375
		1 25	7 498973		
		1	7 488022	0 875	8 542094
		-1 125	7 397673	-2 125	8 383299
		0 25	7 370294		
		2	7 353867	1 875	8 413416
		0 375	7 293634	0 5	8 320329
		0 875	7 277207	0 375	8 309377

		0 375	7 348392	-0 5	8 361396
		1 125	7 203285	1	8 202601
		0 375	7 19781	0 75	8 221766
				0 375	8 262834
		1 25	7 173169	1 25	8 167009
		1 25	7 132102	1	8 161533
		2 375	7 04449	2 375	8 109514
		0	6 94319	-0 875	7 983573
		1	6 951403		
		0 375	6 954141	0 5	7 991786
		0 625	6 954141	0 125	7 969883
		1	6 940452	1	7 950719
		0 5	6 929501	0 5	7 983573
		1 25	6 877481	1 5	7 909651
		1 75	6 90486	1 75	8 024641
		1 375	6 847365		
		-0 75	9 675565	-1 5	10 71869
-0 75	10 85558	-1 125	11 35113	-2 375	12 33402
-0 75	16 52841	-1	16 97194		
-5 625	17 09788	-5 625	17 09788	-6	18 61465
		1 875	9 034907	1 625	10 02601
		-2 375	9 355236	-3	10 38741
		-0 75	8 851472	-1 25	10 00684
		2	8 91718	2	9 938398
		1 375	7 665982	0 625	8 670774
		0 25	10 1848		
		0 625	7 509925	0 5	8 522929
		0	8 928131	-0 125	9 902806
		-1 125	8 862423		
		-1 25	7 964408	-2 375	8 936345
		1 125	8 985626	0 75	10 00958
		1 5	11 02259	0 5	12 03833
		-1 25	13 40726	-1 875	14 46407
		1	7 619439	0 75	8 626967
		1	5 03217		
		-1	15 38125	-1 5	16 39425
		0 375	10 03422	-0 75	11 06639
		-1 875	13 97125	-2 75	14 98699
		-2 25	9 234771	-3	10 26147
		-1 25	8 870637	-1 25	9 828884
		0 125	7 405886	-0 75	8 410678
		-2 75	11 68241	-2 75	12 64066
		4	8 591375	4 5	9 54141
		2 5	7 118412	2 5	8 095825
		0 75	7 786448	0 75	8 843258
		-4 75	7 619439	-6 375	8 54757
		-0 125	15 56742	0	16 54757
		-2 625	11 66051	-3 5	12 59411

1 5	6 798083	0 5	7 780972
6 625	8 525667	6 5	9 412731
-1 75	9 445585	-1 875	10 38193
-1 375	11 31006	-2 75	12 2245
-1 5	10 30527	-3 125	11 1896
-1 875	10 41478	-4	11 34018
1 375	8 646132	1 125	9 546885
		-4 125	9 002053
		-3 25	9 842573
		-3	11 70979
		2	7 742642
		1	7 364819
		3 5	7 559206
		-2 75	8 016427
		1 125	8 824093
		-1 125	11 88227
		-2 5	9 051334
		1	7 88501
		-0 25	15 44422
		-2 25	12 15058
		-2 375	11 29637
		1 25	9 221082
		-2 625	10 57632
		-2 5	11 22245
		1 75	8 388775
		2 125	11 91239
		-3	14 88569
		1 125	7 181383
		-2 875	13 0486
		-2 125	7 359343
		2 375	8 544832
		4 875	12 06023
		0 5	12 94182
		1 375	7 523614
		3 875	12 52019

seod_11	age_11	seod_12	age_12	seod_13	age_13	seod_14
-3	15 92608	-3	16 88706	-3 25	17 9165	-3 375
-1 625	15 82752	-2 625	16 82683			-3 625
-3 75	17 64271	-4	18 62012	-4 375	19 65229	-4 625
-5	13 24298	-4 875	14 23135	-5 125	15 21424	-5 75
-1	20 37235	-1 25	20 83231	-1 375	21 84805	-1 75
-1 75	16 01643			-1 875	17 99042	
		2 5	18 10541	2 75	19 10198	1 875
1 25	17 3744	1 75	17 92197	1 5	18 88022	1 125
-0 125	15 84942	-0 125	15 84942			
-5 125	18 56263	-5 375	19 53183	-5 5	20 53662	
0 375	14 46407	-0 125	15 45791	0 375	16 53388	0 375
-3 5	15 58932	-4 25	16 64339			-3 5
-4 125	16 62423			-5 25	18 61739	-5 625
0 625	16 81314					0 25
1	17 2293	0 5	18 1848			
-2 5	18 40931	-2 75	19 43053			
-4 625	19 99726			-4 625	22 01232	
-5 25	18 00685	-5 5	18 99795	-5 75	20 05476	-5 75
-1	15 06913					
-3 625	12 79671	-4 375	13 78782	-5	14 82272	-6
-2 25	15 78371	-3	16 80219			-4 125
-0 75	19 93155	-0 5	20 91718	-0 625	21 9384	-0 875
1 25	14 83368	0 5	15 75633			
-3	19 54278	-3 25	20 53936	-3	21 58248	-3 25
-3 875	16 34223			-4 25	18 34086	-4 625
-1 375	16 19165	-1 5	16 73374	-1 625	17 70568	-1 875
				-0 375	23 14579	-0 5
-5 5	17 81793	-5 875	18 78987	-5 625	19 82478	-5 875
0 25	15 40315	0	16 33949	0 375	17 36893	
				-2	20 03833	-2 5
-1 125	17 34428	-1	18 3217	-0 5	19 32923	-0 875
-6 625	19 1102	-6 75	20 10404	-7 375	21 09514	-7 875
-0 5	19 12936	-0 625	19 64956	0	20 6872	-0 5
-0 75	13 70021	-1 5	14 7488	-2 25	15 70431	-2 75
-4 25	16 98289	-4 5	17 99315	-4 25	19 02806	
						-0 875
-3 75	12 67625	-4	13 71389	-4 25	14 72142	-4 375
1	16 83778	0 625	17 82067	1 25	18 86653	0 75

0 875	20 11499	1 125	21 18823	1	22 141	
-3	13 53867	-3 875	14 5462	-4	15 56742	-5
-2 25	13 69747	-3	14 74333	-3	15 75633	-3 125
-0 25	15 72895	-0 25	16 67077	-0 375	17 68652	-0 5
0 5	12 71732	0 25	13 72485	-0 25	14 74333	-0 75
-6 875	15 50992	-7	16 49555	-7 5	17 53867	-7
-0 875	15 16769	-1 125	16 16701	-0 75	17 18001	-1 375
-1 625	13 07871	-2 125	14 0616	-2 125	15 08008	-2 125
-1 625	17 03765	-2 75	18 02327	-2 125	19 08282	-2 25
-3 625	18 26694	-3 375	19 29363	-3 625	20 27652	-3 5
-10	20 64339	-9 5	21 58522	-10 625	22 61465	-11 5
-2 5	13 97399	-3 125	14 98426	-3 625	15 9425	
0	17 70842	0	17 70842	0 25	19 24709	
-5 75	13 3306	-5 875	14 28611	-6 625	15 3128	-6 875
0 625	12 92813			1	14 92129	
-3	16 71458	-3 375	17 6783	-3	18 70774	-3 375
				-2 75	21 88912	-3
-1 125	18 81451	-0 875	19 78097	-0 625	20 79398	-1 125
		-3	21 78234			
-3 25	16 44079	-3 125	17 38261	-3	18 43395	-2 875
-0 625	15 68515	-0 75	16 69815	0	17 72211	-0 375
		0 375	13 95756			-0 125
-1 875	16 14237	-1 875	17 19097	-1 75	18 17385	-1 875
-5	15 12115	-5	16 10678	-4 875	17 11157	-5 125
-0 5	13 95756			-1 125	15 9781	-1 875
-3 5	21 11704					-3 75
-3 75	13 94661	-4 125	14 88843	-4 375	15 87406	
0 375	19 20329					0 5
-2 625	19 93703	-2 75	20 89254			-3
-2 75	16 83778			-2 75	18 82272	-2 875
-4 375	21 31143					
-4 875	21 00479					
-2 5	17 57153			-2 5	19 56194	-2 75
0 5	14 51061	-0 25	15 46612	-1	16 48186	-1 5
-9 625	13 74949	-9 125	14 75702	-10 125	15 85216	-11
-3 25	14 46133	-3 875	15 47433	-4 25	16 46817	-4 875

1	14 68857	0 5	15 21424			1
-7	13 60438	-7 25	14 61465	-8	15 59206	-8 5
1 25	12 67351	1	13 68652	1	14 69952	0 75
-3 125	19 46886	-3 125	19 46886	-3 375	20 9692	-3 625
-3 25	17 48118	-3 5	18 48049	-3 25	19 47159	-3 375
				0 5	20 58043	
0 125	18 20671			0 375	20 16427	
				-0 25	18 33265	
0 75	12 97468	1	14 05886			0 875
-1 875	16 88706					
-0 625	14 80356	-1	15 75086			
-5	16 15606	-5 125	17 14442	-5 375	18 14921	-5 625
-1	19 28542					
-2 125	15 45243					
-5 5	20 82683			-5 5	22 85284	
0 125	17 30869					-0 25
0 25	19 86311					
5 25	16 00548	5 5	17 013	3	18 02601	
-2	14 31622					-2 25
-4 375	16 09583	-5 125	17 08693	-5 25	18 10267	-5 75
-2 5	12 89802			-2 625	14 94319	
0 875	12 77481	1	13 71937	0 75	14 73785	0 5
-0 25	18 98426	-0 125	20 03012			-0 5
-6 75	19 78371	-7	20 79124			-7 5
-7 25	16 3039	-7	17 33333	-7	18 3217	-7 25
-2 75	13 41273	-3 25	14 42574	-2 75	15 44969	-3 75
-3 75	13 87543	-3 875	14 8282	-4 125	15 84394	
0 625	16 57495	0	17 53593	0 5	18 58727	0 5
		1	20 01643	1 125	21 03217	0 75
-6 75	20 9911	-6 75	21 5551	-6 25	22 50513	-6 625
-1 75	14 36277	-2 75	15 37577	-3 375	16 36961	-4 625
0 75	13 97399	0 25	14 95688	0 75	15 97262	0 875
1 125	18 25873	1 25	19 24983			1 125
-3	13 46201			-3 375	15 48528	-4
-0 5	13 55236	-0 625	14 53799	-0 375	15 61944	-0 75
0 375	14 91307	0 625	15 91239	0 25	16 91992	
-0 25	13 88364			-0 5	15 86858	-0 75
0 125	15 49076	-0 5	16 48734	0 125	17 50034	-0 375

1 5	14 72416	0 875	15 71253	1 875	16 72005	
-1 625	16 85147	-1 625	16 85147	-1 75	18 40931	
-4 5	18 53799	-4 25	19 57016	-3 75	20 61602	-4
-3 5	17 44832	-3 625	18 45311	-3 125	19 46064	-3 5
-2 5	19 04996	-2 5	19 04996	-2 375	20 58864	-2 75
		-5	19 3347			
0 75	13 38535	0 5	14 41205	1	15 47707	0 75
				-4 375	19 68515	
-5 625	20 82409	-5 5	21 83984	-6	22 81177	-6 25
-2 125	18 50513	-2 125	18 50513	-1	20 01369	-1 25
-5	14 0616	-5	15 07461	-5 125	16 08214	-5 25
-1 75	14 9514	-2	15 91239	-2	16 96372	-2 125
-2	20 96372					
0 25	16 60506	-0 5	17 59343	-0 25	18 57358	
-1 25	17 13347	-1 625	18 11088	-1 25	19 12936	-1 5
-5 375	14 65571	-5 625	15 66324			
-5 5	15 29363	-6 375	16 29295	-6 75	17 32786	-7 75
-5 875	17 02396	-6	18 02053	-6 125	19 0527	-6
		-0 5	14 71321			
-1 25	15 05544	-1 375	16 03833	-1 625	17 05955	-1 75
-0 75	14 48323	-0 625	15 44695	-1 375	16 43806	-1 625
-3 5	19 34839	-3 5	19 34839			
0 125	18 9295					
1 25	15 09103	1	16 03285			1 5
		-3 5	17 06229			
-3	18 13826			-2 875	20 18891	-3 625
-0 75	12 77755	-1 875	13 80698	-2 125	14 77618	-2 25
0	15 73443	-0 5	16 69815	-0 75	17 68652	-0 875
-4 75	14 00958	-5 375	14 9295	-6	15 95072	-6 625
-3 875	20 11499	-3 75	21 06502	-3 5	22 10814	
-2 625	19 92882					
-0 375	16 04654	-0 75	17 06229	-0 5	18 12183	
-2 25	19 03354	-1 875	20 11499			-2
-4 625	16 62423	-4 625	17 60712	-4 375	18 63381	-4 5
-1 375	15 83299	-2 125	16 7885	-2 25	17 7796	-2 625
-3 375	13 27858	-3 75	14 37645	-3 75	15 37303	-4 125
-4 125	16 63244	-4 25	17 68378	-4	18 72142	-4 25
				-1 5	15 25804	
-2 5	14 55989	-3	15 47707	-3 125	16 52841	-3 75
-0 375	13 08145	-0 875	14 13279	-0 875	15 17591	-1 375
-0 25	13 22929	-0 375	14 27789	0 125	15 28816	0
				0 875	23 43327	0 875
-1	20 82136					-1 375
-3 125	14 13826	-3 75	15 13484	-3 875	16 15879	-4 5
-2 5	18 97331	-2 75	19 92882			

-0 75	12 77755			-1 75	14 8501	-2 75
-2 75	18 8282	-3	19 76455	-2 75	20 79398	-3 25
-2 5	13 51951	-3 25	14 52977	-3 75	15 52361	-4 25
-1 625	13 93018	-2	14 94593	-1 75	15 93977	-2 125
-4 75	14 37372	-5 375	15 37029	-5 25	16 37235	-5 375
				-7 375	19 68241	-7 625
-3 25	15 86585	-4	16 83778	-3 75	17 84805	-4 125
-1	14 81451	-1	14 81451			-0 75
0	16 95551					-1 25
-3	14 96509	-3 25	16 01917	-3 5	16 9692	-4 125
-2 875	14 96783	-3 25	15 98083			-3 125
-4 25	16 52019			-4 25	18 52156	
-2	13 16906					
-7 25	18 93224	-7 5	20 01095	-7 875	21 03491	
0 5	14 34634	-0 5	15 28542	-1 625	16 29843	-2 5
-4 625	15 08282	-4 75	16 09309	-4 75	17 08966	-5
-2 875	20 09583	-3	21 08145	-3 125	22 09993	-3 375
-3 25	14 72142	-3 5	15 67146	-3 625	16 72005	
0 125	12 74743	0 25	13 77413	-0 625	14 75154	-1 375
-2	16 78029			-2 25	18 74607	-2 375
-2 875	21 52498	-2 875	21 52498			
0 75	18 43669	0 75	19 46886			0 75
-2 5	16 74743	-3 5	17 70568	-3 25	18 73237	-3 125
1 5	13 80151	0 625	14 7488	1 125	15 77002	0 625
-4 25	15 59206	-4 75	16 57495	-5	17 60164	-5 75
-1 75	20 55852					
		-2 75	15 58111	-2 5	16 62697	-2 5
-5 125	19 24709	-5 125	19 24709			
		1	19 58385	0 75	20 59685	
-4 25	14 141			-4 25	16 2026	
-4 375	12 75017	-4 75	13 72758	-4 625	14 73238	-5 5
-3 5	14 45038	-4 125	15 4141	-3 25	16 43532	-3 625
-2	13 51951	-2 5	14 55715	-3	15 5154	-3 875
-4	19 20602	-4 25	20 19165	-4 25	21 23203	-4 5
1	14 08624	1 125	15 09103	1 375	16 0794	1
-1 5	13 97947	-2 375	14 93771	-2 375	15 96988	-2 5
1 75	12 96372					1 125
0 25	18 10267	-0 125	19 08556	0 375	20 13689	-0 125
4 25	18 90486	4	19 82204	4 5	20 88706	3 75
0 75	13 60164	0 25	14 62834	0 5	15 68515	-0 125

-5 375	14 25051	-5 75	15 26352			
-2 875	16 45448	-3 5	17 42368	-3 625	18 43121	
0 875	21 0705	0 625	22 03696	1 125	23 12115	0 75
0 125	13 90007	-0 375	14 85832	-0 125	15 86037	-0 125
-4	15 31006					-5 375
0	15 44695	-0 375	16 37235	-0 125	17 41273	0
-2 25	18 6256	-1 75	19 64134	-1 75	20 62697	-2 375
-3 125	15 42231	-3 125	16 38877	-2 75	17 38261	
-1 375	13 22108	-1 375	13 22108			-3 25
-5 25	20 67077	-5 125	21 61807	-4 625	22 63381	-5 125
0 625	15 78919					
-0 25	17 01027	-0 5	17 97399			
1 25	15 0089	0 375	15 99179	1 125	17 05133	0 75
-4 125	13 22108	-4 875	14 21766	-5	15 22245	-5
-4 75	14 55168	-5	15 55373	-5 125	16 57495	-6 125
-0 875	18 2423					
-4 25	14 18207	-4 5	15 17865			-5
-0 125	16 06845					-0 375
0 375	14 4449			0 5	16 48734	
-5	18 96235			-4 5	20 9692	-5 125
-1 875	14 94045	-2 125	15 95619	-2	16 93087	-2 625
-0 625	15 47433	-0 5	16 50924	-1 25	17 43737	-1 25
-3 375	18 81999	-3 625	19 87406	-3 75	20 87337	-4 375
1 125	16 16427	1 375	17 20465	1 25	18 20123	0 625
2 375	12 83778	2 625	13 82888			3 25
-2 625	15 70157	-2 625	16 70363	-2 875	17 68925	-2 75
		-2 125	19 31006	-1 875	20 27926	-2
				0 125	16 86516	-0 375
0 875	14 04517	0 25	14 99521	0 75	15 99726	0 75
0 75	14 03422	-0 125	15 06913	0	16 07666	-0 25
		-3 75	17 70842	-3 625	18 7269	-3 5
-3 625	17 295	-4	18 27242	-3 375	19 34565	
-1 375	18 68857	-1 25	19 63587	-1 25	20 64339	-1
-5 75	19 64682	-5 75	20 65435	-5 5	21 67283	-6
-4 375	13 44559	-5	14 43121	-5 25	15 47707	-5 375
-2 875	18 77618	-2 875	19 77276	-2 75	20 8104	-3 125

-7 25	19 80835					
-0 5	17 77139					
-2 875	14 58179	-3 25	15 54004	-3 125	16 5859	-3 5
		-0 25	22 31348	0	23 30732	
0 75	15 16769	0 375	16 09856	0 875	17 10335	0 75
-2 25	15 47981			-2 375	17 48665	-2 75
-4 625	13 46749	-4 625	13 46749	-5 125	14 96509	-5
-3 25	14 91034					
		0 875	15 00342			
-3 75	13 64271	-4 25	14 69952	-4 125	15 6961	-4 625
0 75	17 71389					0 75
0 5	16 64339	-0 5	17 58248	0 125	18 62286	
-4 625	13 11157	-5 375	14 05886	-5 875	15 09377	-6 25
-0 5	14 02601					-1
-2 25	17 66461	-2 875	18 66393	-2 5	19 67967	-2 75
-2	12 94182	-2 5	13 94387	-2 5	14 94867	-2 625
-3 125	19 4141	-3 125	20 39425	-3 125	21 41273	-3 5
-1 5	15 32923	-2	16 37782	-1 75	17 34428	-2 5
						1 375
-5 25	20 75838	-5	21 78782	-4 75	22 7488	-5
-2 125	18 01232					-2 25
-3 375	16 76386	-4 25	17 73306	-4 25	18 73237	-4 5
-4	13 53867	-4 375	14 54894	-4 375	15 56742	-4 875
				-1 75	18 06708	-2 125
1 625	14 39288	1 25	14 87748	1 625	15 9425	1 25
				-1 75	21 52225	
-1 25	19 27721	-0 75	19 84394			
-2 75	18 69678	-2 625	19 72622	-2 5	20 73922	-2 875
-4 75	16 67625	-4 875	17 68925	-4 875	18 705	-5 5
-4 375	18 08898	-4 375	18 08898			-4 5
-5 375	21 23751	-5 25	21 76865			-5 5
-1 375	20 90075			-1 25	22 88022	-1 25
-4	13 91376	-4 125	14 94319	-4 125	15 9781	-4 375
-0 125	13 62081	-0 375	14 67762	0 125	15 66598	0 125
-0 5	15 09925	-1 125	16 07666	-0 75	17 12799	-0 75
-0 125	16 91444	-0 125	17 95756	0	18 94319	-0 25
-1 125	17 86721	-0 875	18 80356	-0 25	19 83025	-0 875
-0 125	17 46475	-0 375	18 47502	0	19 48802	-0 25
-5 75	14 76523	-6	15 76728	-5 75	16 79945	-6
		-4 5	16 62971	-4 75	17 59617	-4 625
-3 125	14 21218	-4	15 21697	-3 75	16 22998	-4 375

-2 5	17 27584	-2 75	17 83436	-3 25	18 75975	
0 5	12 85147	0	13 79055	1	14 84736	0 5
-2 375	15 34018	-3	16 31211	-2 625	17 36619	-3
-2 125	16 59411	-2 875	17 61259	-3 125	18 62286	-3 125
0	15 31828	-0 625	16 25188	-0 375	17 26763	-0 5
-7 625	19 69062	-7 5	20 63244	-8	21 68652	-8
-4 5	21 07598	-4 375	22 02601	-4 375	23 06366	-4 5
				-3 25	22 21492	-3 375
-4	17 78782	-4 75	18 87201	-4 25	19 84121	
0 75	15 82752	1	16 85147	0 75	17 85626	
0 5	14 54894	0 25	15 52361	1	16 53936	0 625
-1 125	13 97399					
-4 375	17 4976	-4 5	18 46133	-4 75	19 38946	-5
-0 625	13 56879	-1	14 57084	-1 25	15 58658	-1
0 375	14 73785	0 375	14 73785			
		-3 75	21 40726			
-4 375	13 53593	-4 25	14 54073	-4 25	15 55647	-5 125
-3 625	17 8371			-3 875	19 80835	
-4	21 27036					
-2 625	14 76797	-3 25	15 77002	-3	16 82409	-4
-0 75	19 12936					-0 625
-1 375	13 58795					-1 5
-4 75	15 64134	-5 25	16 67077			
-5 25	13 99042	-5 875	14 94319	-6 25	15 9425	-6 125
-6 875	14 96783	-6 75	15 96441	-6 75	16 97741	-7 125
1 5	12 09856	0 875	13 11704	1 375	14 141	0 875
-3 25	13 77687	-3 5	14 72964	-3	15 71526	-3 125
-2 25	15 01985	-2 25	16 00274	-2 25	17 02943	-2 25
0 75	13 32238	0 625	14 38467	1	15 36208	0 5
-4 75	13 66188	-5 25	14 60917	-6 25	15 6742	-6 5
1 625	14 15469			1 125	16 12594	
-1 625	12 91992	-2	13 85353	-2	14 91307	-2 375
-1	14 55989	-1 875	15 68241			-3 375
-2 5	17 19097	-3	18 20671	-2	19 22793	-2 375
-2 375	11 72622	-2 75	12 85695	-3 375	13 81246	-3 875
0 75	15 7974					
-2 125	13 20465	-2	14 19302	-2	15 1321	-2 625
-1	16 78576	-1 75	17 77413	-1 5	18 79261	-1 5
-0 25	12 65708	-1 125	13 67283	-1 25	14 66667	-1 625
-3	13 91102	-3 5	14 92676	-3 375	15 8987	-4 125
-3 875	14 85832	-4 125	15 37851			
-3 125	14 92129	-4	15 87953	-3 375	16 91718	-4 125
0 625	12 25188					-0 5
-2 75	14 31622	-3 375	15 28816	-3	16 29021	-3 5
		-1 25	17 24298			

2	13 05407	1 375	13 50856	1 5	14 49692	1 125
		-3 625	20 07392			
-6 625	16 9911	-6 625	17 94387	-6 875	18 97057	-7 75
-2 5	15 75633					
-1 75	17 06502	-2 375	18 05339	-2 125	19 0527	-2 625
		0	14 28611			-0 625
0	12 27926	-0 75	13 2731	-0 75	14 30801	-1 125
		-0 875	16 18891			
-5 875	17 71937	-5 625	18 76249	-5 5	19 75907	-6
-0 5	13 65092					
-0 75	16 13416	-1 625	17 11704			-1 125
-0 875	12 83504	-0 875	13 8371	-0 25	14 88022	-0 75
-5 75	15 90417	-5 875	16 90897	-6 375	17 95209	-7
				0 75	14 66393	
		0 25	19 92608	0 25	20 94456	
-6 5	17 87543	-6 375	18 3655	-7	19 40862	-7 125
-0 5	13 86448	-1	14 83094	-0 75	15 85489	-0 75
-2 5	14 12457	-3	15 0883	-3 5	16 1807	-4 125
-4	14 96509			-4 5	16 92266	-4 5
-5 875	18 92402			-5 75	20 93634	-6
-5 375	20 27652	-5 375	21 27584			-5 625
1	13 6345	1	14 72416	1	15 6961	0 75
-2 875	17 44011	-3 625	18 39836	-3	19 4141	-3 25
-0 75	13 22382					
-1 875	16 87885	-2	17 82341	-0 75	18 88296	-1 125
-3 75	11 69062	-4 125	12 71458	-4 75	13 75496	-5 25
				-4 75	17 5551	-5 125
-0 25	15 68789					
-1 875	15 49624			-2 125	17 61259	
-3	13 50856	-3 75	14 50513	-4	15 49897	-5 125
0	14 35455			0	16 44901	
-0 125	18 2998			0 5	20 33676	0
-0 5	17 89733					-1
-5 875	17 11157	-5 5	18 11362	-6 125	19 15948	-6 125
		-3 25	13 91923	-3 125	14 9295	
		-1 25	20 33676			
-1 625	12 81588	-3 625	13 89733	-3 875	14 88569	-4 375
-6 625	13 31964	-7 125	14 423	-6 875	15 4141	-7 375
-4 875	21 49487	-5 125	22 05339	-5	23 03354	-5 5
0 75	13 1937	-0 125	14 20397	0 125	15 2115	-0 25
-0 125	19 08282	-0 375	20 04928	-0 75	21 10062	
-5	21 4538	-4 5	22 52704	-4 75	23 50719	-5
				1 375	19 61123	
-0 75	17 9603					
-3 375	16 30664	-3 375	17 28679	-4	18 3217	-4 625

		-2 25	14 2204	-2	15 32923	-2 75
-5 625	18 96509	-5 75	19 91786	-5 625	20 95277	-6
-4 625	13 69747	-5	14 74059			
-3 625	16 74743					-5 25
-12 375	12 93361	-11 125	13 89186	-12 875	14 92129	-12 75
-4 125	11 97262	-4 5	12 98563	-4 75	13 99863	-5 5
-4 125	16 86242	-4	17 83436	-3 875	18 8501	-4 125
-3 375	13 50856	-4	14 55715			-4 5
-3	14 19849	-3 75	15 17591	-3 75	16 19439	-4
-4 125	12 63518	-4 5	13 65914	-4 625	14 31896	-4 875
-6 25	13 59343	-6 375	14 57358	-7	15 61123	-7 5
-5 125	18 86105					
-4 75	12 56126	-5	13 61259	-5 25	14 6037	-5 625
-1 625	14 65298	-2 25	15 65229	-2 625	16 70363	-3 25
-0 5	15 92608	-0 375	17 0486	0 25	18 05065	
0 75	16 78029	0	17 70842			
-2 25	15 38398	-2 375	16 4271	-2 375	17 35797	-2 75
3 5	20 91992	3 75	21 49487	3 5	22 45038	
-0 625	17 6345	-0 625	17 6345			
2 25	17 05681	2	18 04244	2 5	19 08282	2 25
-1 5	16 54757	-2 375	17 4757	-2 5	18 5024	-3
-4 625	14 91034	-4 875	15 88501	-4 5	16 92813	-4 625
1 25	19 6386					
-1 375	14 71869	-2 125	15 66872	-2 25	16 69541	-2 75
0 125	12 67351	-0 25	13 63723	-1	14 63381	-1 25
-0 125	12 87885			-0 875	14 91581	-1 25
-4	12 1013			-5	14 13279	
		-5 875	17 6783			
-4 25	12 30664	-4 5	13 31691	-4 875	14 33539	-5 5
-3 625	16 31759			-3 75	18 34086	
-0 375	14 57358	-0 75	15 49624	-0 25	16 55305	
-0 375	13 577			0	15 54825	-0 5
				0 75	16 02464	0 25
-8 375	11 91513	-8 625	12 85695	-9 625	13 90828	
0 75	19 66324					
-1 75	11 90691	-2 375	12 89802	-2 25	13 95209	-2 875
-4 125	17 29226	-4	18 25599	-4 25	19 2909	-4 25
-2 625	14 79261	-3 125	15 73717	-3 125	16 82683	
0 875	12 82957	0 75	13 85626	1 25	14 8501	1
-5 75	17 51951	-5 625	18 55989	-5 75	19 57016	-6 375
				1 25	16 6872	
-5 125	14 63929	-5 5	15 60575	-6	16 6078	-6 25
				-1 375	21 29774	
0 25	19 95619					

-2	17 44559					
				-4 25	21 81793	-4 75
-5	17 67009	-5 25	18 66119	-5 25	19 74264	-5 5
-6 5	17 52772	-6 625	18 48323	-7	19 5948	
0 375	13 58248	-0 125	14 1191	0 125	15 11294	-0 625
-1 125	14 52156	-1 125	14 52156			
1	15 14853	1	15 14853	1 25	16 72827	0 75
-3	19 62491	-3 375	20 60506	-3	21 60438	-3
-4	19 31554	-3 875	20 39151			-3 875
-3 25	20 56126					-3 125
-6 375	15 53183	-6 5	16 57495	-6 5	17 51403	-6 375
-0 25	19 33744					
0 875	16 53936			1	18 59822	
-2 5	19 26626	-2 375	20 22177	-2 5	21 19097	-2 75
-4 5	12 77481	-5	13 72485	-5 375	14 76797	
-2 375	13 7358	-2 5	14 85558	-2 75	15 8412	-3 25
-1	17 56331	-1 75	18 50513	-1 5	19 56468	-2
-3 5	13 01574	-4 5	14 01232	-4 5	15 02259	-4 875
0 875	12 59959	1 25	13 65366	1 25	14 66119	
-4 875	12 01095			-5 875	14 04517	-6 375
-0 5	12 32307	-1 375	13 37714	-1 5	14 35455	-2 125
0 625	15 71253			0 75	17 68652	
-1 875	14 68036	-2 625	15 65503	-2 5	16 67077	-2 75
-2 625	11 26078			0	13 30048	-2 5
0 5	10 52977	-0 125	11 5948	-0 75	12 62423	-1 5
-0 5	13 68104	-1 25	14 72416	-1	15 70705	-1 25
1 25	17 10335	0 75	18 03422			
0 375	10 76797			0	12 85695	-0 5
-1 5	10 83368	-2 5	11 87953	-2 875	12 9117	-3 875
-7	17 77687	-6 5	18 76249	-6 75	19 8193	-7 625
-1 25	17 12799	-2 125	18 07803	-1 875	19 07187	-2 375
-2 25	11 8193	-3 75	12 75565	-4 25	13 81246	-5 25
0 625	17 22108					
		0	12 0575	0	13 04038	
-4 5	12 52019	-5 375	13 62628			-7 25
-4 375	17 05955	-4 25	17 99589	-4	18 96509	-4 5
-3 125	15 83573	-3 25	16 81588	-3 125	17 87817	

-3	15 58111	-3 75	16 57221	-3 5	17 59069	-3 375
-3 625	15 91513	-4 125	16 90349	-3 5	17 90828	
-0 75	15 26899	-0 875	16 30664	-1 25	17 24298	-1 5
-2	16 38056	-2 25	17 34428	-1 75	18 38193	-2 25
-0 125	17 60712	-0 25	18 6694	-0 5	19 63313	-1
		0	18 69405			
-1 25	12 13142	-2 5	13 15537	-2 25	14 13279	-3 125
-0 5	12 60507	-1 5	13 63723	-2 25	14 59822	-2 5
-0 25	17 50856	-0 75	18 57358	-0 875	19 54825	-0 875
-2 75	18 04791	-3 125	19 02259	-2 75	20 03833	-2 875
-1	10 90212	-1 25	11 90144	-1 25	12 95551	-2 125
-3 625	10 70226	-4 375	11 65777	-5 875	12 69268	-7 125
-1 125	11 2553	-1 5	12 29843	-1 625	13 34702	-2 75
-4 375	14 62012	-5	15 64134	-5 25	16 69268	-5 75
-2 5	11 67146	-3	12 69815	-3 875	13 70294	-4 5
3 375	10 89391	2 5	11 84942	2 25	12 8679	1 625
-3 625	16 9473	-4 375	17 95209	-4	18 97604	-4 375
0 5	16 84599	0 375	17 86995			
-4 5	18 04791	-4 5	19 15127	-4 25	20 10951	
-0 25	16 99384	-0 375	18 05339	-0 125	19 07187	
-13 125	15 28542	-11 625	16 33676	-13 625	17 30595	
				-0 625	12 97194	-1 5
-2 25	13 01301	-3	14 141	-3 5	15 06365	-4 125
		-1 375	12 04928			-3 5
-1 375	13 02943	-1 375	14 05065	-1 5	15 02532	-1 875
0 75	10 83094	-0 125	11 81109	-1	12 8104	-1 875
1 25	15 61944	1 25	16 5859			
-2 75	15 05544	-3 625	16 07392	-4 125	17 10609	-4 375
-1 875	11 34839			-2 875	13 37988	-3 25
0	11 80287	-0 875	12 80493	-1 625	13 7796	-2 25
0	13 81793	-0 125	14 83368	0	15 84942	-0 5
1	11 64956	0 75	12 6872	0 5	13 65914	0 5
3 125	11 35387	3 375	12 36961	3 5	13 3744	
0 25	12 06023	0	13 18001	-0 625	14 15195	-1
-2 5	17 74949	-2 625	18 72416	-2	19 7755	
-2 25	18 47775	-2 625	19 47433	-2 125	20 47639	-2 375
-5 25	12 75565	-5 625	13 77139	-6 125	14 74333	-6 875
1 25	15 81109	1 5	16 80767			
0	11 23066	-0 125	12 29295	-0 125	13 30595	-0 25
-1 75	11 24983	-2 25	12 32307	-3 5	13 27584	-4 125
-1 75	12 47639	-2	13 4757	-2 75	14 46407	-3 5

1 125	13 12252	0 75	14 19028	0 75	15 18412	0 625
0 125	12 56126	0 125	12 56126			-0 375
-1	13 88912	-2	14 90212	-2	15 93703	-2 25
-5 75	15 63313	-5 375	16 69541	-5 375	17 67556	
-4 125	12 47912	-4 5	13 57426	-4 75	14 5462	
-1 125	12 00274	-1	12 47912	-1 25	13 46475	-2
-1 25	13 82341	-1	14 91855	-1 375	15 8987	
-3 75	11 3347	-4 5	12 36961	-5 625	13 34429	-6 75
		-5 125	18 1848			
-1 5	14 05339			-2	16 10404	-2 25
0 75	13 10335	0 25	14 12183	0 875	15 17591	0 125
-5 375	15 50445	-5 5	16 5065	-5	17 54962	-5 5
-7 875	13 08693	-8	14 03696			-8 375
0 875	11 37303	0 5	12 42437	0 875	13 40726	0 875
1 5	10 37372	1 125	11 41136	0 5	12 39973	-0 5
0	10 31622	-0 75	11 21971	-1	12 25188	-1 625
-6 125	18 10267	-6 25	19 08008	-5 75	20 15332	-6 125
						-1 25
-1 5	13 36345	-2 125	14 34634	-3	15 3347	-3 375
-0 125	20 67625	-0 125	21 24025	0	22 21766	-0 25
0 625	19 65503	0 75	20 64339			
-1 375	15 79466	-0 75	16 80493	-1 25	17 78508	-1 5
-1 875	12 78029	-2 75	13 76318	-3 25	14 76797	-3 875
-5 125	15 33744	-5 625	16 32033			-5 5
-5	21 15811	-5	21 15811	-4 875	22 7488	
-1 5	13 03765	-1 875	14 06434	-1 75	15 09377	-2 125
-4 25	12 89528	-4 5	13 9165	-4 25	14 91581	-4 875
1	10 85558	0 375	11 85763	-0 25	12 85421	-1 25
-1 875	17 07598	-2 125	18 04517	-1 75	19 06092	-2
-0 125	10 98699	-1 5	12 03559	-1 375	13 00205	-2
0 5	16 84599					
-2 5	19 95619	-2 5	20 96099	-2 25	21 95756	
0 75	19 5373	0 75	19 5373			
-5 875	20 20808	-5 5	21 19097			-6 5
-2 875	15 60849	-3 875	16 61054	-4	17 60164	-4 625
0 625	12 66256	0 375	13 69199	0 75	14 71595	0 75
-4 75	11 98631	-5 25	13 01848	-5 375	14 07255	-5 75
-1	20 1013					
-4 25	13 10062	-4 875	14 06708	-5 375	15 04175	
-1 5	14 47775	-1 75	14 98152	-2 75	16 01095	-3 25
-0 25	19 32923	-0 625	20 29843			
-0 25	16 91171	-0 875	17 92471	-0 125	18 94319	-0 375

0 625	10 85284					
-4 75	11 41958	-4 75	12 41615	-5 25	13 44559	-5 375
-1 5	15 74538	-1 75	16 73922	-1 625	17 73853	-2
-4 875	14 25873	-5 625	15 27447	-6 125	16 29021	
-6	19 65229	-6 5	20 63518			-7 375
-0 5	17 9822	-0 25	19 00616			-0 5
-0 75	11 28268	-2	12 30116	-2	13 30869	-3 125
-0 75	19 88501			-0 5	22 00137	
1	10 67488	0 875	11 55647	1	12 59411	0 5
4 75	14 63929	5	15 17591	5 125	16 17249	4 625
-3 125	12 06023	-4	13 02669	-4 375	14 06434	-4 875
0 5	12 50376	0 125	13 54689	-0 375	14 54346	-0 25
-0 625	15 14579	-0 5	16 11499	-0 75	17 09514	-0 75
-4 25	15 1102	-4 75	16 1232	-5 25	17 10062	-5 25
-8 375	14 73511	-9	15 70979			
-4 125	15 16769	-5 125	16 14511	-5 125	17 15264	-5 625
						-0 625
		-3 25	18 75154			
-1	9 114305	-2 375	10 13005	-3	11 14305	-3 75
2	20 19439	1 5	21 11978	2	22 13279	2
-6 25	18 25325	-6 25	19 23066	-6 5	20 24914	-6 625
-2 75	11 61944	-3 625	12 67899	-4 625	13 64819	-5 375
1 25	11 22519	1	12 24367	0 75	13 21834	-0 25
-0 75	19 40862			-0 5	21 42094	-0 5
-0 125	15 07734	-0 25	16 08214	0 125	17 08145	0
				0 125	13 67556	
-4 5	18 40383	-5 25	19 30185			
4	17 65366	3 375	18 60096			
-0 375	14 6256	0 125	15 62491	-0 25	16 64339	-1
-0 25	11 36208	-1 5	12 34223	-1 375	13 35524	-1 5
-2 875	15 40041	-2 875	15 40041	-2 75	16 91171	
-3 25	16 27378	-3 5	17 23477	-3 375	18 22587	-4
-1 875	17 9822	-1 625	19 01437	-2 375	19 95893	-2 375
0 25	17 19644					-0 25
-4 875	12 27926	-4 875	13 24025	-5 25	14 27515	-5 5
0 5	10 77892			-0 5	12 7447	-0 25
-1 75	15 54278	-2 75	16 55578	-2 375	17 55236	-3 25
0 125	15 04723			0 375	17 03491	0
-4 25	17 65366	-4 875	18 63929	-4 75	19 66872	-5 5
1	12 2245	0 25	13 16359	0 875	14 23682	0 375
-0 375	12 43806	0	13 4976	-0 5	14 44764	-1 125
1 5	13 859	1 125	14 84189	1 25	15 84394	1
1 375	12 91992	1 75	13 39083			1 125
-1 375	20 06571			-1	22 05339	

0 75	15 16222	0 75	15 16222			
-4 25	11 87406	-4 875	12 83504	-5 25	13 87817	-5 75
-5 25	15 16496	-5	16 24093	-5 5	17 19644	-5 375
-5 75	16 34771	-5 75	16 34771			-6 5
-4 375	11 17317			-6 375	13 21834	
						0 5
		1 125	12 38604			
		-3 25	16 28474			-3 625
-5 25	15 63313					-6 125
-4 75	12 1807	-5 75	13 1937	-6	14 20945	-6 375
-2 375	10 85558	-3 5	11 91239	-3 5	12 91992	-4 375
-0 5	14 81725	-1 375	15 78645	-1	16 78576	-1
-1 25	12 43258	-2 25	13 44011	-2 5	14 48049	-3
-5	10 16016	-5 875	11 14579	-6 375	12 09583	-6 625
-2 25	12 21355	-3 25	13 20739	-3	14 27242	
-1 5	11 65503	-2 25	12 62971	-2 25	13 65092	-2 75
		-1 625	15 19507			
-3 75	10 08898	-5	11 10746	-5 875	12 12047	-7
0	10 78713	-0 75	11 72622	-1 25	12 74196	
-0 75	18 17659	-1 375	19 19781	-1	20 25188	
-0 5	12 95551	-1	13 9603	-1 25	14 94867	
-0 25	10 67214	-0 75	11 7755	-1 875	12 74743	-2 625
0 75	16 79124					
-3 75	10 94319	-3 75	11 99179	-3 625	13 00205	-4 375
-2 625	16 3039	-2 75	17 31964	-2 625	18 32991	-3 125
-1 5	17 14168	-1 75	18 11088	-1 375	19 14031	-1 375
0 625	15 41136	0 25	16 44901	0 625	17 41273	0 375
-2 875	11 02259	-3 75	12 04381	-4 25	13 01574	-4 875
-2	9 697468	-2 875	10 68036	-3 5	11 69884	-3 75
-5	16 64066	-4 875	17 05955	-4 5	18 06434	-5 125
-1 25	12 09583	-1 5	12 53388			-2 625
						-0 125
-0 5	17 88638	-0 25	18 89391	0 25	19 89596	-0 125
-2	17 57426	-2	17 57426	-1 75	19 06092	-2 25
						-0 25
0 75	13 91376	0 625	14 9295	0 375	15 98357	0
-1 75	16 77755	-2 5	17 74401	-2 25	18 75975	-2 75
-0 75	9 919233	-2 625	10 94045	-3 75	11 98083	-4 125
		-3 125	13 28679			-4 875
-5 375	11 16496	-6 375	12 13415	-7 25	13 17728	-8
-3 75	12 81862	-4 625	13 88912	-5 125	14 88296	-5 625
-3 25	18 19849	-3 25	19 17864	-2 75	20 24093	-2 875
0 5	14 00137	-0 375	15 00342	-0 875	16 03285	-1 5
-9 5	14 57906					
0 5	11 44148	-0 5	12 42163	-0 625	13 51129	-1 25
0 125	10 96509			-0 75	12 96646	-1 25
0 125	11 2115	0	12 17522	0 125	13 16085	-0 25
-2 75	14 96235	-3 5	15 96988	-2 875	16 97741	-3 25
		-3	16 34223			
-0 75	11 58658	-1 75	12 52841	-2 25	13 59343	-3 25

-5 625	12 67077	-6 25	13 71663			-7 625
-2 625	14 98152					
-2 625	11 34565	-3 125	12 3258	-3 125	13 32512	
-4 375	14 78987	-4 875	15 72622			
0	10 72964	0 125	11 81383	-0 125	12 77481	
0 125	10 37372	-0 5	11 36756	-1 375	12 30664	-1 75
-3 75	14 19576	-4 25	15 19233	-4 25	16 19712	-4 875
						-3 875
1	11 42231	0 375	12 33128	0 375	13 3525	0 125
2 125	10 88843	1 625	11 80014	1 75	12 89802	1 875
0	15 23066	-0 625	15 76454	-0 375	16 78303	-1 25
-6 75	15 47707	-6 375	16 45448	-7	17 41547	-7 625
-4 75	15 47707	-5	16 48186			-5 25
-0 625	13 60438	-0 75	14 12731	-0 625	15 12663	-0 75
						1 25
-3	16 8679	-4	17 91376	-4 5	18 94045	-4 25
				-1 5	18 53251	-2 25
-3 5	18 09172	-3 75	19 08008	-3 5	20 10951	-3 625
-2	14 29979	-1 75	15 37577			-1 75
-1 5	20 0219					-2
-3 625	13 51951	-4 25	14 48049	-4 375	15 5154	-4 5
		-3 5	16 03833	-3 875	16 98015	-4 5
-0 75	20 92813	-0 5	21 90281			-0 5
-1	12 91718	-1 5	13 92745	-1 25	14 94593	-1 75
-4 75	13 99042	-5	15 04175	-5 25	16 03833	-5
-1 625	15 37303					
-2 125	16 63792	-2 375	17 60164	-2 125	18 61739	
-0 5	17 91923	-0 25	18 95688	-0 25	19 97262	-0 5
0 5	19 98905					
				-2 125	16 95551	
-0 125	17 18549			-0 25	19 1102	-0 5
-5 375	13 7796					
		-2 875	14 30527			-3 5
-3	14 81177	-4 125	15 78919	-4 375	16 82409	-4 625
-0 625	14 6256	-1 25	15 60575	-1 5	16 67077	-2
-5 125	20 73374					
-2 25	15 35661	-3	16 33128	-2 75	17 35524	
-2 75	18 79808			-2 75	20 7885	-2 875
-1 5	15 65229	-2 25	16 6078	-2 25	17 60438	-3
-2	20 85695					
				0 125	15 03354	
-3 75	16 55852					
-3 875	19 98631	-4 125	21 00479			-4

-6	12 80493	-5 75	13 82888	-5 875	14 86927	-6 125
-5 75	19 19507	-5 625	20 17522	-5 5	21 18823	-5 5
				6 125	20 61875	
						-1 75
-3 75	17 08419	-4 25	18 10267			-4 5
				-0 75	15 78097	-1
				0 625	20 36687	0 375
0 875	11 30732	0 75	12 3614	1	13 3306	0 625
-2 625	18 06434	-2 75	19 01437	-2 375	20 06571	-2 625
						-4 5
-5 875	18 02327			-6	20 05202	
-9 5	18 69405	-9 75	19 7755	-9 75	20 76386	-10
-4 5	11 77823	-5	12 79124	-5 125	13 80972	-5 5
-7	15 88227	-7 25	16 89802	-7 875	17 87817	
-1 25	14 6694					
0 75	11 38946	0 25	12 43258	0 25	13 39357	0 375
-5 75	13 80698	-6 625	14 88569	-7 625	15 86037	-7 5
-4	12 564	-4 5	13 53867	-5 5	14 56263	
-3 125	12 65435	-3 5	13 68652	-3 25	14 72416	-3 875
-3	14 64203	-3 75	15 62491	-3 75	16 64887	-4 5
1 375	12 87337	0 75	13 83162	1 125	14 92402	0 75
		-3	19 98905			
-2 5	16 21355	-3 25	17 21013	-3 5	18 25599	-4 25
-2 75	11 21424	-2 75	11 21424	-4	12 64613	
0 5	12 49829	-0 125	13 52498	0 5	14 55441	-0 25
-0 25	9 486653	-0 625	10 4449	-0 5	11 42505	-1 375
-3 75	15 47433	-4	16 43806	-3 625	17 45106	-3 75
0 875	10 16564	0 25	11 18412	-0 125	12 17796	-0 75
-4 75	13 13347	-5 375	14 10814			
0 375	10 54346	-0 625	11 47707			-3 5
-5 375	17 84257	-5	18 78439	-5 375	19 83847	
-5 75	13 0924	-6 25	14 09719	-6 125	15 09103	
-2 25	12 81314	-3	13 83162	-3 125	14 87748	-3 5
0	14 39836					
-4	18 41478	-4	19 38125	-3 75	20 4627	-4
-3 75	19 24435			-4	21 23751	
-4 25	17 94661	-4 5	18 9295	-4 625	19 98631	-4 75
1 5	11 28542	1 125	12 33676	1 5	13 36893	1
-7 375	10 0616					
-0 5	9 976728	-1 5	10 98699	-1 625	12 01917	-2 125
-5	17 7577	-5 75	18 79535	-5 5	19 75907	-6 375
-2 125	17 79877	-2 5	18 81177	-1 75	19 83025	-2 5
-2 375	17 71389					
-3 875	18 09993	-4	18 61739			-4 875
-2 75	17 6564					
0	17 63997					

-1 75	17 94387	-1 75	18 98152			-2 5
						-4 5
-4	17 88638	-4 25	18 94319	-4 375	19 93977	-4 625
						-2 75
1 5	17 78508	1 125	18 81451	1 75	19 8987	1 25
-4 375	17 25667	-4 375	17 25667			
-3 25	16 66804	-4	17 60712	-3 75	18 62286	-4 25
-0 25	16 70089					-1
-0 75	17 74127	-1	18 22313			
-3 25	17 10335	-4	18 09446			
-3 875	17 0705					
-2	17 02396	-2 5	17 94387	-2 125	19 06913	-2 25
-2 875	17 0486	-3	18 03696	-2 375	19 08282	-2 625
-2 625	16 91718	-2 625	17 97399	-2 875	18 94319	-3
1 125	16 93908					0 625
-1 25	16 85421	-1 75	17 87269	-1 625	18 93224	-2
-1	15 62218	-1 125	16 60233	-1	17 59617	-1 125
-2 25	16 50103	-3	17 48939	-3 25	18 51335	-3 5
1 25	16 57221	0 375	17 57153	1 25	18 56263	0 5
-0 75	16 92813	-1 5	17 4538	-1	18 46407	-1 5
-3 75	16 17796	-4	17 26763			
-3	16 02464	-3 25	17 01027	-3	18 01232	-4 25
-3 625	16 01095	-3 75	16 99658	-4	18 05339	-4 5
-4 5	15 88501	-4 625	16 90623			-4 875
-2	14 8063	-2 75	15 80287	-2 5	16 7666	-3 5
-0 125	14 72964	-0 625	15 74812	-0 375	16 79945	-0 5
-0 125	14 85832					
1 5	14 83641	1 25	15 75907	1 75	16 78303	1 5
-5 25	14 77071					
-0 5	14 69952	-0 375	15 73443			-0 75
-4 875	14 59548	-5	15 60575			
-2 25	15 54004	-2 25	16 59411			
-1 625	15 22519	-2 5	16 22998			
0 75	15 67146	0 5	16 20534	1 125	17 23203	1 375
-6 25	15 2909			-6 5	17 26489	-6 625
-4 625	14 89938	-4 75	15 87953	-5	16 91444	

-4 625	11 21697	-5 625	12 2245	-5 25	13 22656	-5 875
-2 625	13 90281	-3 5	14 81451	-3 25	15 78371	-3 375
-4 25	13 65092	-5 125	14 65571	-5 25	15 66051	
-7 125	13 61533	-7 25	14 58453	-7 875	15 63313	-8 625
-2 5	13 63997	-2 75	14 58179	-2 625	15 67967	-2 75
-1 75	15 04723	-1 75	15 04723			-3
-3 875	14 62286	-4 25	15 61944	-4 5	16 62423	-5
2 375	14 51061					2 5
-2	14 40657	-2 625	15 38398	-1 875	16 4052	
5	14 36003	4 625	15 34018	5 125	16 32854	5 125
-4 75	14 23956			-5 375	16 34771	-5 625
-2	14 32991	-2 25	15 26899	-1 125	16 282	-1 375
-5 5	14 25599			-6 625	16 22177	
				-0 875	16 27378	-1 25
		-1 25	15 11567	-0 75	16 1013	-0 875
-1 5	14 21766	-2 5	15 21971	-2 375	16 2026	
-1 5	14 06434	-2 25	14 99795	-1 875	16 07118	
-2 75	14 04791	-3 625	15 00068			
-2 375	13 94935	-3 125	14 96235	-3 375	16 0219	
		0 5	14 96783	0 625	16 01643	0 25
-5 875	13 88638	-6 375	14 88296	-7	15 91786	
0 25	14 00137					
-1 25	11 85763	-2	12 90623	-1 75	13 9165	-2
-3 25	13 85626	-3 75	14 85558	-3 875	15 91513	-4 25
-2 25	13 88364	-2 875	14 85558	-3	15 90965	-3 375
0	12 80219	-0 125	13 79055			-0 75
-1 5	12 77207					
-3 125	12 75291			-5	14 74333	-6
0 25	12 73648	-0 25	13 71937	-0 125	14 75702	-0 75
						-0 375
-1 125	13 33881	-2 25	14 37372	-2 375	15 37029	-3 125
-4 375	13 37714	-5 5	14 34634	-5 875	15 34839	-5 75
-0 625	13 36071	-1	14 31348	-0 375	15 35113	-0 625
-3 875	13 1499	-3 875	14 25599	-3 875	15 15674	-3 875
-7 375	13 19918	-7 5	14 22861	-8 25	15 31554	
-1 125	13 37166	-2 25	14 3436			
1	13 66735	1	13 66735	0 25	15 17043	0 375
-6 5	13 13895	-6 75	14 08077	-7 375	15 08008	
-2 75	13 08693	-3 5	14 12457			
-1 625	12 9692	-1 875	13 99863	-1 875	15 04175	
1 25	13 01301	1 375	14 0835	1 5	15 10472	1 25
0 5	13 59617	0 375	14 06982			
-1 5	11 90144			-2 375	13 97399	-2 875
-2	12 99932	-2	13 96578	-2 5	14 96235	-2 125
-1 5	12 94182	-2 125	13 94661			
0 875	12 57495	1 125	13 577	1 375	14 60917	1 125

-0 125	12 91718			-0 125	14 89391	-0 75
-2 25	12 82135	-3 25	13 82615	-2 75	14 84736	-3 75
-2 375	11 72348	-2	12 79945	-1 75	13 8152	-2 125
0 25	11 70157	-1 125	12 73374	-1	13 74949	-1 75
-1 75	11 78371	-2 5	12 72279	-2 5	13 73853	-3 25
1	11 84668	0 5	12 7666	0 75	13 84805	
0 5	11 64956	-0 625	12 65161	-1 625	13 64271	-2 5
0 5	11 57016	1	12 6653	1	13 65366	1
-0 875	12 13142	-1 125	12 64887	-1 5	13 65914	-2 25
-0 75	11 60301	-2 125	12 58042	-2 625	13 65366	-3 5
-4 625	11 66872	-5 125	12 6872	-5 875	13 6783	-6 125
-4 125	11 85763	-4 125	12 82409			-6
-0 25	12 59411	-0 625	13 48939	-0 5	14 52704	-1 375
0 125	12 53936			-2 125	14 49144	-2 75
-4 875	12 32033	-5 375	13 35524	-5 625	14 35729	-6 375
-3 25	12 39151	-4 625	13 40999	-5 375	14 39288	-6 5
-3 75	12 19165					-4 625
-2 625	12 21355	-3 625	13 15264	-3 5	14 19302	-4
-3 75	12 1807	-4	13 18275	-4 625	14 15195	
-0 25	12 23272	-1 5	13 20192	-1 5	14 24778	-2 625
-9 75	12 17248					
-4 25	12 22724	-4 875	13 26489	-5 25	14 28337	-5 5
0 875	12 09583	0 75	13 14716	1	14 1629	0 375
-0 75	12 17796					-3 25
						-4 625
0 25	11 95072	0 375	12 97194			0 125
				0 5	14 05339	-0 125
		-0 75	13 01027			-2 625
0 75	11 92882					
-2 75	11 98083	-3 5	12 95825	-3 625	13 97399	-4 25
-3 625	11 91513					
-2 25	11 78097					
1 25	11 79192	1 25	12 87064	1 125	13 90828	0 75
0 375	11 83573	-1	12 85421	-0 75	13 88364	-1 25
		0	11 85216	-1	12 83231	-2
4 125	10 73238	4 5	11 80561	4 5	12 75838	4 375
						-4 75
		1 25	11 86037	1 25	12 846	

1 125	10 84189	0 25	11 80287	0 5	12 78029	-0 375
-3 375	10 69131	-5 125	11 70705	-6 25	12 76934	-7 5
-0 625	10 68857	-1 375	11 62765	-1 75	12 66256	-2 25
		-1	11 70979	-1 25	12 67077	-1 75
		-0 5	12 51198	-1	13 57153	
-2 125	11 44695	-3 125	12 46544	-3 375	13 46475	-4
-0 25	11 49897	-1	12 49829	-0 75	13 51677	-1
0 875	11 48802	0 25	12 44079	0 25	13 47023	-0 125
		-3 125	12 49281	-4	13 3963	-4 5
-2	11 34565			-2 625	13 3525	-3 5
0 75	11 37029	-0 125	12 29569	0 25	13 37714	-0 25
		-2 25	12 34771			
				0	13 38262	-1
-6 25	11 79192	-6 625	12 23272	-7 5	13 25941	-8 25
0 375	11 14853	-0 625	12 13415	-0 75	13 20192	-1 125
				1 25	13 14168	
1 25	11 18686			1	13 19096	0 5
						0 875
						-0 875
-4 625	11 21424					
-3 5	10 98973	-4 5	12 01095	-5 125	13 06776	-5 875
-1 375	11 06913	-1 25	12 0575	-1 5	13 0486	-2 625
-4 375	11 04997	-5 125	11 98631	-5 875	12 97468	-6 5
0	11 00616					
-1 75	11 02532	-3 25	11 92608	-3	12 99384	-4
-1 5	10 86927	-2 75	11 89596	-3 25	12 93908	-3 75
0 625	10 76797	0	11 66051	-0 625	12 67625	-1 125
0 75	10 89117	-0 5	11 90691	-0 5	12 93635	
				-0 375	12 92539	
0 5	10 88569	0 375	11 8987	0 125	12 93635	-0 5
				0	12 83504	-0 75
				-1 625	12 96646	
1 75	9 73306	1 625	10 83094	1 75	11 81656	1 25
-1 5	9 774127	-2 25	10 77071	-1 75	11 80014	-2
-1	9 702943			-3	11 75633	-4
-2 375	9 722108	-3 25	10 77071	-4 25	11 75359	-5 5
2 375	9 831622					
0 875	9 648186	0 75	10 64476	0 75	11 66872	0 125
0 75	9 686516					
						-1 75
0 375	9 639973	-0 25	10 62012	-1 25	11 55099	-1 75
0 5	9 615332	-0 625	10 61738	-1	11 62218	-1 875

		6 375	12 55305			
-2 125	10 4668	-2 75	11 46886	-3	12 49281	-3 75
-1 375	10 51335	-2 25	11 53183	-2 625	12 59138	-3
0	10 53251	-0 25	11 51814	-0 625	12 60507	-0 75
1	10 5024	0 625	11 56468	0 5	12 58864	0 375
-1 625	10 40383	-3 125	11 40041	-3 5	12 46817	-4 375
0 75	10 35455	0 75	11 37851			
-2 75	10 93224	-2 75	10 93224	-3 75	12 43532	-4 75
				0 5	12 47091	-0 25
-0 25	10 28063	-1 5	11 28542	-1	12 33949	-1 75
-0 75	10 32444	-1 5	11 27995	-3 25	12 31759	-4 375
-3 625	10 20123	-4 5	11 26352	-5 125	12 2601	-5 625
-1 625	10 34086	-2 25	11 35113	-3	12 29295	-4
		0 5	11 27721			-0 375
0 75	10 23409					
		0 5	11 24435	0 75	12 24093	0 75
				1	12 23546	
-2 5	10 16838	-3 375	11 17317	-3 875	12 18891	-4 75
0 75	10 11636					
2 375	10 13279	2 125	11 14853	1 375	12 08487	1 125
0 75	10 05613	0 75	11 12936	0 25	12 13963	0 125
-0 875	10 08077	-2	11 05818			-4 125
-2 625	10 08077	-3 75	11 06913	-4 25	12 10678	-4 5
0 5	10 09993	0 5	11 16222	1	12 19165	0 5
-4 25	10 06982	-4 75	11 10472	-5 25	12 0219	-5 25
0 25	10 09993			0 25	12 08214	
-1 375	9 976728	-1 75	11 06365	-1 875	11 99179	-2 875
-1 75	9 883641					
-2 125	9 87269	-3	10 89665	-3 25	11 94524	-3 625
0 5	9 812457	0 625	10 91855	0 5	11 88227	0
-1	9 886379	-1 5	10 92129	-2	11 8412	-2 25
-2 125	9 826146	-2 875	10 79535	-2 625	11 86311	-3 25
-0 5	9 785079	-1 875	10 81177	-2	11 81383	-3 625
0 5	8 711842	-0 5	9 752225	-1 375	10 68309	-2 75
-3	9 486653	-3 625	10 58179	-4 125	11 55373	-5
-1	9 52772	-2 375	10 5462	-2	11 54552	-2 5
1	9 601643			-0 125	11 58658	-1 25
0 875	9 500342	0 5	10 59822	0 375	11 5948	0
-3 5	9 379877	-4 375	10 39288	-5 25	11 40862	-6
-0 875	9 366187	-1 5	10 43395	-1 625	11 3922	-3
1 875	9 453798	1 875	10 41478	1 125	11 41684	0 75
0	9 412731	-0 75	10 39562	-1 875	11 41958	-3 125
-1 75	9 300479	-3 625	10 39014	-4 5	11 34292	-6

-1 5	9 327858	-3 125	10 31075	-4 25	11 31554	-5 375
1 125	9 793292	1 125	9 793292			-1 5
0 5	9 215606	0 25	10 28611	-0 75	11 29637	-1 75
0	9 727584	0	9 727584			
		-2 125	10 25873	-3 25	11 18138	-4
1 25	9 226557	0 5	10 17933			0 5
0 5	9 199179	-0 5	10 21766	-1 625	11 1896	-2 5
		1 75	10 11636	2 25	11 17865	1 75
-2	9 015742	-2 75	9 968514	-3 25	10 98699	-4 5
0 5	8 985626			-1 25	11 0527	-2 25
-0 125	8 939083	-1	9 952087	-1 25	10 97331	-1 75
1 25	9 54141	1 25	9 54141			-1 125
0	8 98015	-0 75	10 03422	-1 75	10 97331	-2 125
0 75	8 906229	-0 375	9 919233	-0 125	10 93498	-0 5
1 875	9 034907	2	10 01506	2 25	11 01711	
1	9 467488	1	9 467488	-5 125	10 94319	-2 625
-2 25	11 68515	-3 125	12 68446	-3 5	13 72211	-3 75
-2 875	13 39083	-3 25	14 40657	-4	15 37577	-4 375
-1 5	19 11567					
-5 875	19 6386	-5 625	20 6653	-5 75	21 67283	-6
-3	11 32923	-3 5	12 4271	-4	13 44832	-5
1 625	10 93498	0 375	11 93155			
0 25	9 746749	0 5	10 77071	-0 25	11 76454	-1 25
		-0 125	13 31417			
0 125	11 0089	-0 5	11 96715	-0 75	12 96372	-2 25
-3 25	10 88569	-4	11 88227	-4 5	12 91718	-5
-3 25	9 916495			-5 125	12 00821	-6 5
1 25	11 02532					
0 25	13 04312	-0 625	14 12731	-1 125	15 0883	-1 75
-1 875	15 41958	-2 5	16 45996	-3	17 53046	-3 75
0 75	9 763176	0 375	10 7488			
1	7 066393	1 125	8 104038	0 625	9 144422	
-1 125	17 41547	-1 125	18 45859			-1 125
						-1 625
						-5 625
-4 25	11 2909	-5 125	12 23272			-6 875
-1 625	10 90486	-2 125	11 91786	-2 625	12 90623	-3 5
-1 75	9 355236	-3 375	10 39562	-3 875	11 4141	-4 5
-3	13 61533	-3 5	14 65024			-3 75
4	10 56263	3	11 55647	3	12 55578	2 75
2 5	9 133471	1 25	10 10267	0 75	11 10472	0 625
0	10 30253	-0 5	10 83641	-1	11 82204	-2 25
-6 875	9 52772	-7 375	10 56263			-10 125
		-1	18 5243	0	19 57563	-0 5
-4 25	13 59343	-4 875	14 59822	-5 375	15 58385	-6

-0 375	8 687201	-1 875	9 749487	-2 875	10 72416	-3 25
6 125	10 40931	5 875	11 45517	5 625	12 46817	5
-2 625	11 46064			-3 375	13 38262	
-3	13 21834	-3 125	14 21218	-3 375	15 23888	-4 125
-4 75	12 20534	-4 5	13 22656	-4 875	14 23409	-4 875
-5 375	12 31485	-6 5	13 31964	-7	14 41478	
0 875	10 53525	0 75	11 54004	0 75	12 55852	0 25
-5 875	10 07529					
-4 25	10 88022	-5	11 92334	-5 75	12 84873	-6 5
-3 5	12 6872	-4 125	13 74127	-4 125	14 72416	-4 75
1 75	8 665298	1 875	9 7577	1	10 68583	0 75
0 5	8 273785	-1 5	9 300479	-2 25	10 30801	-3 5
2 625	8 517454	1 25	9 50308	1 25	10 50513	0 75
-4 25	8 941821	-6 125	9 993155	-7 25	10 98973	-8 375
1	9 7577	0 75	10 81725			-1 125
-2	12 81588	-2 375	13 8152	-3	14 83094	-3 5
-3 5	9 963039	-4 25	10 98699	-4 5	12 01095	-5 125
0 875	8 881588	-0 125	9 859001	-0 125	10 88569	-1 375
				-1 375	18 4668	
-2 375	13 06229	-3 25	14 10541	-3 25	15 12389	-3 375
-2 75	12 2245	-2 75	13 22382			
1 125	10 14647	1 125	11 24435	1 125	12 25736	0 75
-4 125	11 61944					
-3 375	12 20534	-4 125	13 18823	-4 5	14 21492	-5
0	9 295004	1 125	10 31348	1 25	11 32649	0 375
				2 5	14 8501	2 125
-3 5	15 88775	-4	16 85421	-3 5	17 86721	-4
0 25	8 197125	0 5	9 21013			
-3 125	14 00137	-4 125	14 98699	-4 125	16 01643	-4 625
-3	8 262834	-2 875	9 273101	-2 75	10 34908	-3 25
		1 625	10 52977			
4 5	13 02669	4	14 07803	4 625	15 01437	4 5
0 5	13 82615					
0 75	8 418891			0 125	10 48597	-0 5
		3 75	14 44764	3 75	15 45791	3 125
		-8 125	9 207392	-7 125	10 19576	-7 375
		-1 75	12 18617	-2 625	13 2512	-3 5
		-5	13 2731	-6 5	14 28063	-7 625
		-11 625	16 16975	-12 125	17 1718	-12 5
		0 5	8 810404	0 75	9 820671	0 375
		-2 25	8 232718	-3 875	9 207392	-4 75
		-3 375	11 68789	-3 75	12 62697	-4 75
		1 5	9 930184			-0 875
		0 125	7 578371	-0 625	8 588638	-1 375
		0 75	13 18823	0 5	14 17933	0 25
		5 625	7 381246	5 75	8 358659	5 5
		0 75	8 372348	0 75	9 38809	
		1 5	7 457906	1 625	8 479124	1 375
		-15 5	8 153319	-16 125	9 138946	-16 75
		0	7 60575	-0 375	8 605065	-0 375
		-8 125	15 94524	-8 375	16 91444	-8 625

-4 625	11 65777	-5 25	12 62423	-5 5
-1	9 585216	-2 375	10 60096	-3 5
-3 875	10 04244	-5 125	10 97057	-6
-1 25	11 43874	-2 125	12 3833	-3 5
1 375	7 011636	1 25	7 969883	0 375
-10 25	8 21629	-10 125	9 160849	
-0 5	8 670774	0 125	9 585216	-0 875
-0 625	7 011636	-1 625	8	-2 875
-5 375	14 67762	-6	15 5948	-6 5

age_14	seod_15	age_15	master_acd_c	master_acd_c	master_acd_c	master_acd_c
			3 86	3 95	3 92	3 89
			3 57	3 64		3 68
18 93224	-4	19 94524	3 6	3 65	3 73	3 71
18 83368	-3 625	19 87953	3 32	3 46	3 63	3 63
20 66256	-4 625	21 6783	3 76	3 88	3 97	3 99
			3 63	3 63	3 57	3 7
16 17522			3 15	3 25	3 29	3 34
22 90212	-2 125	23 82204	3 59	3 56	3 73	3 59
			3 03	3 02		
			3 2	3 24	3 27	
			3 24	3 39	3 37	3 43
			3 49	3 51	3 57	
20 11225			3 75	3 75	3 6	3 77
19 95072	1 125	20 93634	3 67	3 7	3 85	3 73
			3 34	3 23	3 53	3 47
	-6 375	22 55989	3 44	3 47	3 52	3 56
			3 18	3 2		
17 47023	0 375	18 42847	3 49	3 53	3 55	3 54
18 63381	-2 875	19 63587	3 48		3 69	3 79
19 66324	-6	20 63518	3 3	3 31	3 38	3 38
19 75907			2 77	2 78	2 91	2 95
			3 38	3 44	3 51	3 58
					3 72	3 69
	-5 125	24 07392	3 65	3 67	3 66	3 63
			3 2		3 22	
21 02396			3 37	3 45	3 43	3 32
			3 35	3 37	3 43	3 5
15 80835	-6 25	16 82409	3 06	3 23	3 22	3 27
18 83641	-4 125	19 80014	3 77		3 96	3 98
			3 33	3 35	3 35	3 46
22 95962	-0 625	23 87953	3 54	3 62	3 71	3 67
			3 4	3 5	3 58	3 66
22 62286	-3 375	23 5729	3 6	3 55	3 6	3 61
19 30185	-4 5	20 31485	3 71	3 79		3 88
18 69131	-2	19 718	3 64	3 7	3 72	3 79
24 15606	-0 5	25 11431	3 8	3 92	3 94	3 96
20 80493	-5 5	21 80424	3 63	3 65	3 77	3 74
	-0 125	19 36756	3 61	3 4	3 65	3 5
21 00479	-2 25	21 98768	3 39	3 44	3 49	3 54
20 33949	-0 375	21 27036	3 63	3 71	3 72	3 71
22 14647	-7 5	23 08829	3 69	3 76	3 75	3 77
			3 25	3 3		
21 63724	-0 375	22 67214	3 32	3 37	3 42	3 4
16 69541	-3 375	17 67283	3 02	3 13	3 25	3 33
	-5	20 95004	3 5	3 56	3 54	3 58
			2 93	2 98	2 96	3 12
23 15127	-0 875	24 05202	3 95	4 05	4 09	4 08
15 75359	-4 625	16 6872	3 32	3 44	3 47	3 59
19 82204	0 75	20 7885	3 79	3 81	3 83	3 82

			3 11	3 17	3 19	3 26
16 564	-5 5	17 5551	3 58	3 75	3 86	3 95
16 76386	-3 25	17 71663	3 14	3 25	3 34	3 35
18 68857	-0 375	19 68515	3 25	3 33	3 33	3 34
15 75086	-1 25	16 72827	3 19	3 26	3 35	3 42
18 54073	-7 375	19 50171	3 42	3 5	3 61	3 51
18 21218	-1 25	19 17317	3 62	3 67	3 81	3 81
16 08487	-2 375	17 05955	2 83	3 11	3 25	3 26
20 06023	-2 625	21 05407	3 65	3 89	3 86	3 88
21 24846	-3 625	22 1848	3 13	3 31	3 37	3 41
23 63039	-12 125	24 6078	3 73	3 82	3 89	3 86
	-3 625	17 96578	3 23	3 27	3 35	
			3 58	3 72	3 68	3 74
			3 76	3 84	3 79	3 92
			2 86	2 95	3 01	3 12
	-0 125	21 21287	3 36	3 4		3 5
16 29295	-7 25	17 25941	3 74	3 87	3 81	3 99
	1	16 89254	3 66	3 48	3 68	3 75
19 68515	-3 125	20 67899	3 5	3 67		3 89
22 89665	-3	23 80835	3 68	3 8	3 75	3 78
21 89459	-1	22 84189	3 63	3 74	3 79	3 72
			3 59	3 64	3 64	3 62
			3 64	3 67	3 65	3 63
19 41136	-2 625	20 39973	3 71	3 82	3 77	3 78
18 7269	-0 5	19 6742	3 2	3 44	3 56	3 6
15 9425	-0 25	16 9473	2 69	2 86	2 94	2 94
19 22519	-2 125	20 12594	3 69	3 73	3 72	3 73
18 12731	-5	19 10198	3 6	3 77	3 84	3 82
			3 28			
16 95004	-1 5	17 8809	3 99	4 04	4 11	4 21
24 17796			3 41	3 51		3 53
	-4 75	17 86174	3 85		4 11	4 09
			3 57	3 31	3 53	3 48
22 27515	0 5	23 2553	3 51	3 57	3 56	3 55
22 94593	-2 875	23 95072	3 69	3 74	3 79	3 77
19 81656	-2 625	20 78029	3 69	3 81	3 89	3 88
			2 99	2 99		
			3 52	3 58	3 64	3 41
			4 03	4 11	4 14	4 1
			3 66	3 68	3 72	3 75
			3 72		3 78	3 79
20 56947	-2 875	21 62081	3 88	3 9		3 85
			3 68	3 79	3 72	3 8
			3 11	3 22	3 23	
17 46475	-1 75	18 39288	3 33	3 46	3 5	
16 79671			3 41	3 55	3 36	3 65
17 51403	-4 75	18 4668	3 81	3 85	3 9	3 97

17 25941	0 75	18 17385	2 89	3	2 99	3 01
16 60506	-9	17 57153	3 5	3 57	3 59	3 62
			3 72	3 78		
15 75359	0 625	16 69815	3 33	3 41	3 54	3 6
	-1 25	19 2909	3 55	3 7	3 69	3 7
22 02053	-4 125	23 00342	3 34	3 37	3 16	3 44
	-5 625	23 75633	3 76	3 82	3 85	3 83
20 49555	-3 5	21 52225	3 56	3 56	3 64	3 62
			3 36	3 34	3 47	3 41
			3 6		3 75	3 71
			3 52	3 55		
			3 31	3 33	3 42	3 43
			3 51		3 57	3 58
			3 25	3 31	3 33	3 31
16 01369	0 875	17 013	2 63	2 67	2 77	2 78
	-1 875	20 87611	3 56	3 66	3 71	3 85
			3 65			3 82
19 15948	-5 625	20 15879	3 63	3 71	3 71	3 71
			3 19	3 27	3 3	3 26
			3 36	3 29	3 57	3 63
			4 02	4 08	4 12	4 14
20 35318	-0 375	21 36893	3 54	3 52	3 58	3 55
			3 76	3 92		
			3 15	3 32	3 44	3 49
	4 625	19 9781	2 88	2 56	2 71	2 79
17 27858	-2	18 21766	3 62	3 71	3 72	3 87
19 16496	-6	20 1013			3 69	3 66
			3 52	3 49		
	0 5	16 63792	3 01	2 9	2 98	3 03
	-3 25	16 92266	3 4	3 5	3 64	
			3 25		3 33	3 3
15 70431			3 26	3 36	3 4	3 44
22 05339	-0 25	22 97878	3 7	3 64	3 65	3 62
22 78439	-7 375	23 73443	3 87	3 83	3 9	3 95
19 3347	-7 375	20 34223	3 9	4 01	4 06	3 99
16 45175					3 47	3 56
			3 23	3 38	3 46	3 47
19 52635	0 75	20 57221	3 25	3 26	3 37	3 38
			3 34	3 46		
22 07255	0 5	22 99247	3 33	3 39	3 48	3 44
			3 19	3 25	3 32	3 31
23 51814	-6 75	24 52293	3 71	3 75	3 85	3 82
17 3525	-4 5	18 32717	3 17	3 21	3 25	3 35
16 96099	0 5	17 93292	3 3	3 3	3 42	3 44
21 23751	1 125	22 13552	3 14	3 14	3 16	3 13
16 43258	-4 25	17 41547	3 67	3 79	3 85	3 87
16 58864	-0 625	17 56879	3 24	3 34	3 45	3 48
	-0 5	18 86105	3 04	3 1	3 22	3 2
16 87337	-1	17 84805	3 22	3 29	3 3	3 48
18 53525	-0 375	19 52909	3 44	3 52	3 51	3 45

	1 875	18 70774	2 65	2 68	2 82	2 86
			3 56	3 6	3 71	3 68
21 5551	-4 25	22 57084	3 68	3 73	3 79	3 83
20 44901	-3 75	21 45927	3 72	3 77	3 46	3 8
21 62354	-3	22 58179	3 98		4 1	
			3 96	4 04	4 06	4 01
16 42437	0 875	17 40452	2 89	3 02	3 2	3 26
			3 37	3 41	3 5	3 47
23 81383	-6 125	24 75291	4 03		4 15	4 18
21 05407	-1 75	21 9822	3 25	3 37	3 38	3 39
17 08966	-5 375	18 09172	3 88	3 91	4	4 06
			3	3 1		
			3 27	3 35	3 57	
			3 65	3 67	3 73	
17 93292	-2 5	18 90486	3 61	3 57	3 78	3 79
			3 45	3 58		
			3 4	3 36	3 31	
	-0 75	20 52293	3 57	3 65	3 76	3 79
20 15332	-1 625	21 11704	3 71	3 74	3 78	3 84
			3 41	3 48	3 56	3 57
	-4 625	18 141	3 42			
18 28611			3 11	3 25	3 28	3 3
			3 67		3 72	
20 03833	-6 375	20 98015	3 52	3 57	3 54	3 61
			3 53	3 48	3 55	3 62
18 04244	-1 875	19 04449	3 51	3 61	3 71	3 72
17 40452	-2	18 35181	3 55		3 79	3 79
	-4 25	22 90212	3 77		3 82	3 86
	-0 5	22 90212	3 55	3 66	3 59	3 75
			3 49	3 59	3 65	
18 05613	1 25	19 06092	3 18	3 13	3 22	3 27
	-4	20 14784	3 37	3 53	3 54	3 57
21 13621	-3 25	22 13005	3 98	4 05	4 08	4 06
15 81109	-2 125	16 69541	3 38	3 38	3 68	3 64
18 62834	-1 125	19 62491	3 33	3 5	3 59	3 62
16 93361	-7 375	17 93019	3 16	3 32	3 35	3 36
	-3 75	24 04107	3 61	3 59	3 46	3 62
	-3 625	23 90144	3 69	3 75	3 81	
			3 45		3 58	
22 13552			3 89	4 02	4 07	4 06
19 65229	-4 75	20 60506	3 42	3 49	3 52	3 51
18 83094	-4 75	19 86037	3 34	3 49	3 65	3 79
16 35044	-4 125	17 33607	3 97	3 82	4 01	4 14
19 7399	-4 25	20 73101	3 96	4 03	4 06	4 05
			3 63	3 71	3 74	
17 55784	-4	18 53525	3 24	3 4	3 43	3 52
			3 89	3 9	3 88	3 96
16 16427	-1 125	17 18823	3 29	3 39	3 38	3 44
16 282	0	17 27036	3 49	3 56	3 63	3 71
24 49555	1	25 44832	2 99	3 06	3 19	3 13
23 89049			3 58	3 75	3 73	
17 22108	-4 875	18 11088	3 41	3 51	3 58	3 56
			3 69	3 7	3 76	

15 82752	-3 125	16 79124	3 42	3 51	3 63	3 68
21 8152	-3 375	22 74607	3 54	3 6	3 66	3 61
			3 55	3 63	3 63	3 63
16 53662	-4 75	17 51677	3 74	3 83	3 92	3 92
16 91171	-2 5	17 90007	3 67		3 82	3 89
17 4538	-5 875	18 3436	3 59	3 59	3 84	3 89
20 72005	-7 5	21 62081	3 7	3 67	3 77	3 77
18 88843	-4	19 8768	3 87	4 03	4 06	4 06
17 32238			3 01			
20			2 94	2 99	2 88	3 05
18 0178	-4 375	18 94593	3 5	3 59	3 64	3 67
17 98768	-3 125	19 04449	3 41	3 48	3 51	3 58
			3 47	3 55	3 63	3 64
			3 54	3 6	3 69	3 75
					3 7	3 76
	-9	22 91034	3 56	3 61	3 62	3 63
17 36345	-2 875	18 26694	3 34	3 31	3 4	3 4
18 11362	-5 375	19 1102	3 86	3 89	4 01	4 04
23 15127	-3 75	24 09035	3 45	3 57	3 55	3 56
	-4 75	18 67488	3 34	3 42	3 53	3 6
15 7399	-1 375	16 73648	3 2	3 17	3 29	3 35
19 74538			3 59		3 68	
	-2 5	25 02396	3 75		3 79	3 91
21 46201			3 02	3 06	3 16	3 14
19 69884	-3 25	20 7447	3 24	3 29	3 37	3 4
			3 03		3 2	
16 8104			3 27	3 34	3 4	3 36
			3 16	3 23		
18 57084	-6	19 60849	3 23	3 37	3 42	3 35
			3 17		3 26	
			3 68	3 69	3 72	3 8
17 67283	-2 375	18 63107	3 54	3 66	3 7	3 87
			3 86	3 91	3 96	3 95
			3 8		4 03	4 08
	0 625	22 57906	3 46	3 47	3 52	
			3 21	3 28		
	-4 25	18 16838	3 45	3 59	3 59	3 61
15 75359	-5 625	16 68446	3 35	3 41	3 53	3 55
17 42368	-3 75	18 47502	3 21	3 28	3 38	3 31
16 54757	-3 75	17 4757	3 19	3 3	3 42	3 41
22 26147	-4 375	23 23066	3 75	3 81	3 87	3 9
17 07871	1	18 05065	3 61	3 7	3 75	3 82
16 93908	-2 75	17 97399	3 38	3 49	3 51	3 54
	-2	17 07598	3 39			3 53
15 9781			3 15	3 23	3 32	3 49
21 16359	0 25	22 13005	3 42	3 46	3 5	3 52
			3 76		3 77	3 72
21 93292	4 25	22 81451	3 32	3 39	3 41	3 4
16 64066	-0 5	17 61807	3 28	3 37	3 39	3 44
			3 53	3 46	3 54	3 56
			3 24	3 31		

			3 18	3 06	3 01	3 11
			3 86	3 9	4 01	3 98
	-3 875	20 43532	3 3	3 32	3 49	3 53
24 07666	0 875	25 06229	3 55	3 49	3 31	3 48
16 85969	0	17 83162	3 31	3 28	3 32	3 38
			3 77	3 75	3 8	
18 24778	-5 375	19 26899	3 3	3 47	3 46	3 48
18 40383	0	19 39494	3 45	3 48	3 57	3 63
			3 28	3 31	3 44	
21 6345	-2	22 60643	3 49	3 5	3 6	
			3 97	4 03	4 03	4 06
15 74538			3 23	3 35	3 33	
			3 48	3 48	3 51	
23 68515	-5 5	24 60506	3 67	3 75	3 82	3 75
			2 97	3 07	3 16	3 22
	-1 25	20 99658	3 39	3 41	3 5	3 49
18 06981			3 3	3 36	3 43	3 41
16 20534	-5 625	17 17728	3 36	3 54	3 61	3 7
			3 19	3 29	3 24	3 21
17 60438	-6 625	18 57632	3 15		3 26	3 23
			3 44	3 69	3 7	
			3 57	3 64	3 65	
17 20465	-5 125	18 17112	3 55	3 61	3 67	3 74
19 07461	-0 375	20 10404	3 51	3 59	3 7	3 75
			3 12	3 21	3 34	3 27
			3 39	3 44	3 43	3 44
			3 38	3 34	3 39	3 33
21 89459			3 6	3 58	3 56	3 62
17 91923	-2 75	18 94319	3 52	3 6	3 69	3 67
18 43669	-1 25	19 50992	3 46	3 75	3 71	3 82
21 83436			3 38	3 21	3 33	3 42
19 18412	0 75	20 16701	3 59	3 58	3 47	3 52
			3 77		3 82	3 84
			3 7	3 71	3 7	3 75
15 81383	2 5	16 81588	3 41	3 41	3 39	3 31
18 68036	-3	19 67967	3 6	3 68	3 77	3 82
21 24025	-2 25	22 23956	3 57	3 63	3 73	3 74
			3 4	3 45	3 48	3 63
17 79329	-0 375	18 82272	3 41	3 57	3 57	3 52
			3 4	3 38	3 48	
			3 53	3 67	3 69	3 76
17 07324	0 75	18 03148	3 52	3 55	3 72	3 78
			3 56			3 61
17 01848	-0 125	18 06981	3 43	3 54	3 66	3 66
19 72074	-3 875	20 64066	4 04	4 12	4 14	4 17
			3 75	3 89	3 98	3 96
	-3 5	21 33333	4 06	4 11	4 16	4 21
21 73853	-1 25	22 65024	3 8	3 81	3 79	3 9
22 71595	-5 875	23 68789	3 88	3 96	4	4 03
16 48186	-5 25	17 44285	3 26	3 32	3 42	3 43
21 80698	-2 875	22 80903	3 77	3 86	3 82	3 86

			3 58	3 67	3 69	3 71
			3 76	3 69	3 83	3 81
17 57153	-3 625	18 59548	3 45	3 65	3 67	3 49
			3 54	3 59	3 58	3 56
			3 48	3 53	3 59	3 56
			3 78	3 82	3 89	3 9
18 17659	0 625	19 08829	3 43	3 47	3 42	3 53
18 5243	-3	19 56468	3 75	3 83	3 8	3 85
15 98083	-5 25	16 95825	3 5	3 58	3 66	3 69
			3 53	3 58		3 74
			3 6	3 68	3 68	3 69
			3 44	3 45	3 44	3 52
16 68994	-4 625	17 68925	3 15	3 33	3 44	3 56
			3 86	3 91	3 97	3 92
20 77481			3 56	3 64	3 63	3 69
			3 51	3 62	3 68	
16 11225	-6 5	17 03217	3 63	3 77	3 83	3 99
17 14168	-1 25	18 05339	2 84	2 93	2 98	2 99
20 65708	-2 75	21 65092	3 56	3 59	3 65	3 75
15 95346	-2 625	16 94182	3 46	3 52	3 6	3 68
22 43669	-3	23 49076	3 26	3 27	3 41	3 43
18 36003	-2 5	19 37577	3 55	3 59	3 76	3 73
23 73443	1 5	24 71458	3 77	3 76	3 49	3 73
23 80835			3 94			3 95
			3 55		3 71	
21 04586			3 46	3 49	3 58	3 59
19 75633	-4 125	20 7447	3 5	3 54	3 59	3 57
			3 2	3 2		
16 58864	-4 75	17 52225	3 69	3 77	3 83	3 77
19 01711	-2 25	20 03559	3 4	3 52	3 65	3 62
16 90349	1	17 89459	3 2	3 19	3 35	3 33
			3 45	3 44	3 5	3 56
			3 6	3 66	3 63	3 72
21 70568	-2 625	22 68857	3 6	3 67	3 7	3 7
19 72348	-5 75	20 65708	3 46	3 52	3 61	3 63
20 61875	-4 625	21 62628	3 77	3 84	3 79	3 77
23 78097			3 9	3 98	4 05	4
23 91239	-1	24 86242	3 47	3 55	3 57	3 42
			3 22	3 25	3 32	3 41
16 92266	-4 75	17 91102	3 28	3 35	3 41	3 31
16 7091	-0 125	17 64271	3 3	3 44	3 67	3 71
18 1191	-0 75	19 16769	3 19	3 24	3 33	3 38
19 9206	0	20 93634	3 33	3 43	3 42	3 42
20 82409	-1	21 77139	3 19	3 26	3 26	3 4
20 44901	-0 25	21 46475	3 3			
			3 81	3 91		
17 77413	-6 25	18 74333	3 63	3 72	3 7	3 72
18 61739			3 19	3 29	3 38	3 42
			3 49	3 64	3 68	3 75
17 23751	-3 875	18 22587	3 72	3 84	3 95	3 99

	-4 25	20 81588			3 43	3 48
15 83299	0 25	16 83778	3 34	3 37	3 32	3 39
18 35181	-3	19 3347	3 61	3 64	3 73	3 72
			3 7	3 74	3 72	
19 61396	-3 625	20 66804	3 73	3 87	3 8	3 82
18 34908	-0 625	19 29637	3 49	3 68	3 79	3 86
22 68036	-8 125	23 65777	3 96	4 02	4 02	4 01
24 0794	-4 625	25 01848	3 44	3 57	3 64	3 58
23 27173	-3 375	24 17522	3 46	3 49	3 55	3 58
	-4 375	21 78234	3 61	3 67	3 72	3 85
			3 34	3 44	3 51	3 54
	0	24 90349	3 42	3 49	3 69	3 63
17 59343	0 875	18 53251	3 13	3 04	3 16	3 05
			3 38	3 53	3 53	3 6
20 39973	-4 875	21 37714	3 74	3 88	3 95	3 96
			3 53		3 58	
16 58316	-1 25	17 52225	3 67	3 85	3 92	3 99
			3 22	3 42		
			3 65			
			3 61	3 66	3 73	3 61
					3 36	
16 55852	-5 5	17 54415	3 74	3 85	3 8	3 94
			3 45	3 51	3 47	
	-4 875	21 76318	3 84	3 88	4 03	4
	-4 75	25 25667	3 43	3 44	3 3	3 36
17 78782			3 58	3 53	3 73	3 71
					3 74	
22 14647	-0 75	23 04723	3 37	3 42	3 45	3 5
16 62149	-1 5	17 59343	3 4	3 4	3 5	3 59
			3 61	3 76	3 8	3 89
16 96099	-6 25	17 9165	3 45	3 44	3 54	3 59
17 9384	-7 5	18 91581	3 12	3 14	3 19	3 23
15 12663	0 875	16 10404	3 03	3 07	3 17	3 21
16 73101	-3 125	17 68925	3 53	3 74	3 54	3 83
18 01232	-2 25	19 08282	3 47	3 55	3 64	3 66
16 34223	0 625	17 30869	3 11	3 23	3 3	3 27
16 57769	-7	17 56058	3 79	3 91	3 95	4 01
			3 16	3 21	3 32	3 23
15 89322	-2 375	16 87064	3 4	3 48	3 6	3 79
17 63997	-3	18 65298	3 45	3 49	3 47	3 77
20 23272	-2	21 24298	3 79	3 84	3 89	3 87
14 86105	-4 25	15 73169	3 23	3 27	3 39	3 48
			3	2 91	2 94	3 05
16 17796	-2 75	17 14716	3 19	3 32	3 47	3 44
19 77276	-1 25	20 76386	3 3	3 36	3 52	3 58
15 66598	-1 625	16 65435	3 33	3 32	3 44	3 5
16 90349			3 24	3 29	3 41	3 41
			3 76	3 8	3 87	3 99
			3 26	3 32	3 31	3 38
17 9384	-4 125	18 93224	3 58	3 7	3 73	3 73
15 24709			3 1	3	3 13	3 2
17 28953	-3 5	18 28063	3 45	3 63	3 69	3 77
			3 29	3 36	3 45	3 47

15 51814	1	16 48734	2 75	2 88	2 89	2 93
			3 7	3 74	3 8	3 81
			3 43	3 48	3 51	3 78
19 95346	-7 875	20 94456	3 51	3 52	3 57	3 61
			3 54	3 55	3 66	3 67
20 15058	-2 375	21 08966	3 4	3 43	3 45	3 54
16 26831			3 23	3 29	3 24	3 32
15 31006	-1 125	16 26557	3 31	3 3	3 38	3 41
			3 4	3 47	3 49	3 56
20 73648			3 58	3 55	3 53	3 58
			3 62	3 77	3 87	3 94
19 13758	-1 25	20 1013	3 88	3 89	3 93	4
15 84942	-0 875	16 82957	3	3 12	3 16	3 27
18 95688	-7	19 8987	3 69	3 74	3 85	3 86
			2 97	3 06	3 17	
			3 57	3 61	3 64	3 64
20 4627	-8	21 39083	3 69		3 81	3 8
			3 03	3	3 18	3 21
16 81862	-0 875	17 79877	3	3 12	3 19	3 33
			3 89	3 92		
17 19918			3 17	3 34	3 35	3 47
17 92471			3 37	3 63	3 63	3 66
21 94661	-6 375	22 92129	3 63	3 64		3 67
23 31006	-5 25	24 24914	3 39	3 47	3 48	3 52
16 71184			3 82	3 94	4 05	3 92
	-1 25	22 37372	3 73	3 79	3 86	3 87
20 4627	-3	21 44011	3 77	3 79	3 86	3 9
			3 27	3 34		3 47
19 91239	-1	20 86516	3 64	3 71	3 69	3 7
14 75975	-5 375	15 6961	3 29	3 41	3 55	3 6
			3 79	3 83	3 85	3 89
18 49966	-5 25	19 50992	3 89	3 9	4	4 04
			3 31	3 22	3 56	3 42
			3 72	3 73	3 79	3 84
16 53388	-5 625	17 47023	3 2	3 45	3 54	3 57
	-0 75	18 38193	3 51	3 63	3 71	3 72
21 35524	-0 25	22 3436	3 09	3 13	3 25	3 36
20 84599	-1	21 77413	3 44	3 53	3 56	3 56
20 15879	-6 5	21 11978	3 6	3 68	3 7	3 75
			3 47	3 56	3 69	3 84
			3 53	3 52	3 59	3 58
			3 21	3 34	3 42	
			3 36	3 29		3 37
15 86858	-4 875	16 83504	3 23	3 3	3 35	3 37
16 37509	-7 5	17 37714	3 34	3 47	3 5	
24 04654	-5 5	25 00479	3 65	3 69	3 7	3 68
16 22177	-0 5	17 19918	2 69	2 8	2 81	2 8
	-0 875	23 07734	4 12	4 18	4 19	4 22
24 52019	-4 875	25 42368	3 85	3 95	3 91	3 91
			2 94			
	-1 375	21 90281	3 44	3 46	3 5	3 51
19 29637	-4 75	20 26831	3 5	3 56	3 6	3 68

16 27378	-3 25	17 22382	2 87			
			3 04	3	2 69	
21 93292	-6 25	22 9076	3 77	3 81	3 88	3 84
			3 48	3 57	3 63	3 72
19 82751			3 74	3 8	3 82	3 87
15 86858	-13 5	16 85695	3 67	3 69	3 74	3 69
15 01437	-5 75	15 97262	3 1	3 17	3 29	3 38
19 86858	-4 125	20 83504	3 79		3 88	3 91
16 59959	-4 625	17 5551	3 66	3 77	3 83	3 92
17 16906	-4 375	18 18754	2 99	3 13	3 16	3 27
15 6742	-5	16 63244	3 46	3 5	3 62	3 76
16 57221	-7 875	17 53046	3 53	3 62	3 7	3 76
			3 81	3 86	3 86	3 94
15 59754	-5 875	16 564	3 02	3 44	3 4	3 52
17 61533	-4	18 62286	3 4	3 46	3 47	3 53
			3 28	3 37	3 49	
			3 18		3 33	3 38
	0 5	20 75291	3 62	3 72	3 76	3 77
18 43669	-2 25	19 36482	3 37	3 42	3 54	3 66
			3 36	3 55	3 53	3 63
			3 55	3 66	3 64	3 67
	3 5	24 4627	3 3	3 33	3 42	3 43
	-0 5	21 16359	3 63	3 64	3 77	3 72
20 11499	2 25	21 09514	2 76	2 93	3 05	3 05
19 50719	-2 75	20 42984	3 32	3 38	3 5	3 58
17 9603	-4 75	18 89665	3 56	3 66	3 75	3 7
	0 5	23 74538	3 45	3 45	3 49	3 45
17 73032	-2 125	18 66119	3 42	3 52	3 71	3 74
15 60301	-1 25	16 57769	3 34	3 37	3 46	3 53
			3 05	3 12		
15 90965	-1 75	16 84599	3 46	3 47	3 53	3 58
	-5 625	16 1807	3 2	3 05	3 62	3 61
			3 83	3 85	3 93	3 94
15 34018			3 21	3 42	3 47	3 53
			3 57	3 6	3 66	3 76
	-1 25	18 55989	3 22	3 32	3 35	3 41
16 57769	-0 5	17 51677	3 29	3 32	3 44	3 49
17 06502	0 25	18 00958	2 75	2 82	2 84	2 98
	-10 875	15 88501	3 39	3 47	3 54	3 62
	0 625	23 68241	3 58	3 64	3 65	3 67
14 96783	-3 25	15 93703	3 31	3 44	3 47	3 53
20 29021	-5 125	21 28405	4	4 07	4 06	4 13
	-3	18 78987	3 23	3 33	3 38	3 45
			3 8	3 85	3 87	
15 78371	0 875	16 79398	3 56			3 77
20 55305	-6	21 53867	4 21	4 24	4 32	4 32
			3 47	3 41	3 57	3 66
17 62902	-6 75	18 63655	3 88	3 9	4 04	4 06
			3 45	3 42		3 58
			3 6	3 7	3 68	3 71
			3 59		3 72	3 65

	-2 125	21 35797	3 44			3 51
22 81177	-4 75	23 84942	3 81	3 96	3 95	3 97
20 68994	-5 625	21 66735	3 56	3 6	3 62	3 68
			3 65	3 6	3 79	3 81
16 10951	-0 75	17 09514	3 49	3 59	3 62	3 66
			3 31	3 38	3 43	3 49
17 64819	0 75	18 66119	3 74	3 74	3 81	3 95
			3 69		3 78	
22 66667	-2 875	23 58658	3 41	3 43	3 5	3 47
			3 36	3 46		
22 36003	-4 25	23 35934	3 73	3 73	3 7	3 7
23 53183	-3 5	24 50924	3 48	3 51	3 52	3 54
18 51061			3 9	3 92	4 02	4
			3 55	3 6	3 54	3 57
			3 33	3 38		3 36
			3 17	3 3	3 31	3 3
22 2642	-2 875	23 18412	3 55		3 63	
			3 85	3 87	3 95	4 17
16 7666	-3 125	17 77687	3 47	3 59	3 69	
20 60506			3 24		3 31	3 4
			3 38	3 57		
15 99179	-4 625	16 98015	3 59	3 84	3 86	3 88
			3 46	3 5	3 58	
	0 75	16 62697	3 47	3 58	3 57	3 6
			3 51		3 53	3 51
15 03901	-7 125	15 96715		3 23	3 39	3 41
15 33196	-2 25	16 34771		3 29	3 29	3 47
	0 25	19 72074		3 8	3 88	3 9
					3 32	
17 66735	-3 25	18 66393			3 33	3 39
14 37098	-0 75	15 35113			3 68	3 7
13 56057	-2 25	14 54073			3 69	3 76
					3 47	3 49
16 69541	-1 5	17 73306			3 57	3 63
					3 56	3 61
13 83984	-0 75	14 77071			3 38	3 4
					3 26	
					3 43	3 68
13 86448	-4 375	14 83641			3 55	3 58
20 88433	-7 625	21 84531			3 45	3 76
20 13142	-2 625	21 11704			3 28	3 31
14 77071	-6 25	15 78097			3 48	3 56
	0 625	21 23751			3 43	3 39
					3 33	
	0 125	15 01437			3 28	
15 64682	-7 5	16 6078			3 54	
					2 98	3 11
20 02464	-4 5	20 97467			3 91	3 83
					3 54	3 69

18 58727	-3 5	19 60849	3 48	3 53
	-3 875	19 95072	3 78	3 77
18 22861	-1 25	19 23066	3 71	3 69
	2 875	20 88433	2 79	2 61
19 3922	-2 25	20 38056	3 58	3 61
20 63244	-0 75	21 66461	3 85	3 86
			3 34	3 4
	-0 5	21 68925	3 25	3 36
15 14579	-3 25	16 13963	3 71	3 78
15 61396	-2 75	16 61602	3 22	3 17
20 56126	-1	21 56331	3 65	3 69
21 12526	-3 375	22 06434	3 35	3 26
			3 08	
13 93566	-2 875	14 99795	3 49	3 66
13 75222	-7 5	14 64476	3 56	
14 31075	-3 25	15 26899	3 67	3 75
17 65092			3 47	3 54
14 72964	-4 75	15 67693	3 12	3 24
			3 85	
13 87817	1 25	14 85558	3 2	3 11
19 95346			3 78	3 79
			3 28	3 37
			3 71	3 7
			3 88	3 87
			3 48	
	0	16 02464	3 2	3 46
			3 43	3 46
13 97125	-1 75	14 89665	3 45	
16 03559	-4 125	17 01027	3 62	3 72
14 10267	-3 75	15 01985	3 16	3 17
16 02464	-2 125	17 03491	3 92	3 93
			3 34	
13 83984	-2 25	14 79808	3 41	3 47
	0 75	19 54004	3 38	3 43
			3 94	
18 10267	-4 75	19 14305	3 73	3 74
14 32717	-3 5	15 29911	3 97	4 05
14 79808	-2 375	15 81656	3 36	
			2 97	
16 86516	-0 25	17 81246	3 58	3 63
14 63929	0 5	15 64956	3 31	
	3	15 31006	3 46	3 53
15 17591	-1 5	16 10678	3 31	3 28
			3 85	3 77
21 47296	-2 5	22 48871	3 86	3 91
15 73169	-7 125	16 7091	3 63	3 71
	1 5	19 8193	3 63	
14 31622	-0 625	15 24435	3 4	3 49
			3 81	
14 29979	-4 375	15 25257	3 61	3 74
			3 76	3 78
15 47707	-3 75	16 42984	3 6	3 69

			3 53	3 49
16 19165	0 75	17 16906	3 52	3 49
			3 33	3 3
15 0527	-0 5	16 05476	3 53	
16 93908	-2 25	17 92745	3 17	3 1
			3 76	3 8
	-5 25	16 51198	3 46	3 53
14 44216	-2 125	15 436	3 31	3 46
	-2 75	17 83984	3 47	3 73
14 36003	0	15 34292	3 58	3 66
			3 65	3 71
17 06776	-2 5	18 0397	3 66	3 75
16 14784	0	17 12799	3 72	3 66
18 50787	-5 25	19 52635	3 29	3 2
16 08761	-8 5	17 05955	3 63	3 46
14 40383	0 625	15 37577	3 65	3 64
			4 1	
13 3744	-1	14 3436	3 33	3 43
13 27584	-2	14 20397	3 45	3 57
21 18275	-5 875	22 09719	3 67	3 65
13 64271	-1 5	14 58179	3 05	3 18
16 34497	-3 75	17 33333	3 36	3 45
23 23614	-0 25	24 17522	3 65	
			3 61	3 47
18 7707	-1 625	19 7399	3 67	3 68
			3 61	
15 82752	-3 75	16 77207	3 37	3 5
18 32991	-5 75	19 36208	3 77	3 85
			3 4	
	-4 875	24 72005	3 29	3 26
16 0794	-2 25	17 02669	3 38	3 41
16 00548	-5	16 9473	3 68	3 68
13 83162	-1 75	14 84189	3 55	3 62
			3 67	3 7
20 07118	-1 75	21 06229	3 64	3 7
14 01232	-2 25	14 98699	3 87	
			2 79	
	-3	23 90965	3 54	3 51
			3 57	3 54
23 28542	-6 125	24 18617	4 08	4 08
18 62012	-4 5	19 61123	3 58	3 62
15 72895			3 46	3 55
	1 5	23 05818		3 74
15 02532	-5 875	16 00821	3 94	3 98
			3 58	3 55
			3 4	3 44
				3 7
				3 87
17 03491				3 23
			3 29	3 35
	-0 125	22 60643		3 87
19 90144	-0 375	20 90075		3 55

			3 37	3 42
14 46407	-5 125	15 40315	3 82	3 86
18 75154	-2 125	19 73443	3 5	3 74
	-5 875	18 22587	3 73	3 75
			3 86	3 85
22 62834	-8 125	23 52361	3 83	3 87
	-1	16 25462		3 86
21 013	0	21 91102	3 56	3 57
			3 23	
14 33812	-3 875	15 27721		3 52
				3 64
13 58795				2 77
17 15264				3 09
15 01985	-5	15 99726		3 64
15 54004	-0 75	16 51472		3 34
18 17385	-1	19 12663		3 7
18 11088	-5 5	19 11841	3 56	3 54
				3 96
18 15195	-5 625	19 12663		3 75
22 09446	-1 625	23 09103		3 6
	-2 875	21 7796		3 85
12 16427				3 41
23 14031	2	24 13689	3 31	3 33
			4 26	4 24
21 2731	-6 875	22 25051		3 42
14 63381	-6 25	15 59206		3 78
14 20397	0 25	15 16496		3 5
22 41478	-0 625	23 32375		3 52
18 09993	0	19 10472		3 8
				3 51
			3 58	3 59
			3 72	3 78
	3 875	21 58522	3 27	3 31
17 60438	-1 375	18 55989	3 39	3 42
14 37098	-1 875	15 29637		3 32
	-3 25	18 96783	3 39	3 4
19 25804	-4 125	20 2245	3 48	3 49
20 95004	-2 75	21 93019		3 55
20 16427	-0 375	21 12252		3 73
15 29363	-5 5	16 24914		3 65
13 79877				3 86
18 60096	-3	19 56194	3 69	3 74
18 00958				3 65
20 68446	-5 25	21 65914	3 67	3 61
15 19507	0 625	16 16427	3 72	
15 47159	-1 5	16 39151	3 25	3 33
16 88706	1 25	17 82341		3 41
15 38672	1 125	16 33676	3 56	3 59
	-1 625	23 98357		3 62

				3 33
14 88296	-6 125	15 79466		3 33
18 17659	-5 625	19 15401		3 95
18 87201				4 24
				3 64
16 2245				3 65
	-0 125	15 35934	3 65	3 72
18 20397			3 68	3 79
18 60917				3 69
15 20876	-7	16 18344	3 54	3 65
13 95756	-4 25	14 86105	3 34	3 51
17 81246	-1	18 78166		3 68
15 44148	-3 25	16 42163		3 74
13 12252	-7	14 15195	3 72	3 87
				3 82
14 74059	-3 5	15 63039	3 26	3 53
			3 51	
13 11978	-7 75	14 13279	3 52	3 61
			3 61	3 74
			3 57	3 51
	-1 75	16 91444		3 76
13 79603	-2 5	14 79535		3 38
			3 39	3 41
13 99863	-4 375	14 98426	3 52	3 67
19 33196	-3 25	20 32033	3 36	3 46
20 1807	-1 375	21 12799		3 64
18 46954	0 625	19 43874		3 51
14 05339	-5 25	15 03628		3 85
12 70089	-3 875	13 64819	3 02	3 09
19 13758	-5 25	20 06571	3 54	3 48
14 58453	-3 125	15 54552	3 44	
14 29158				3 23
20 90349	0	21 86448	3 7	3 75
20 0575	-2 375	20 99932	3 21	3 28
15 08282				3 39
16 93908	0 125	17 99589		3 46
19 75359	-2 5	20 82409	3 44	3 51
12 95277	-5 375	13 9165	3 05	3 15
			3 52	3 49
15 27447	-5 625	16 26557		3 65
14 23956	-8 625	15 16222		
15 8768	-6 25	16 82136		3 73
21 16085	-2 75	22 15469	3 69	3 67
17 05955	-0 375	18 00137	3 58	3 62
				3 37
14 45038	-1 5	15 46612	3 31	3 38
13 92745	-1 75	14 85558		2 93
			3 38	3 39
14 12731	-0 625	15 06639	3 28	3 34
18 03696	-3 75	18 94045	3 13	3 12
	-3 375	19 3922		3 62
14 61738				3 51

15 69062	-8 875	16 65708		3 65
			3 52	3 65
			3 43	3 52
				3 37
			4	4 08
			3 12	3 15
13 35797	-2	14 29979		3 41
17 20739	-4 625	18 18754		3 8
				3 97
18 46133				
14 3436	-0 125	15 29363	3 51	3 58
13 87817	1 625	14 83094		3 24
17 83436	-1 5	18 73237	3 26	3 33
18 48323	-7 375	19 40862	3 68	3 56
18 48597			3 74	3 79
				3 88
				4 09
16 15332	-0 75	17 08966	3 12	3 25
14 61191				3 51
19 88227	-4 5	20 90075		3 75
19 5729	-2 125	20 55578		3 43
21 09514	-4	22 11088	3 47	3 48
17 37166				3 63
23 02806	-2 25	24 02464		3 66
16 47091	-4 25	17 44832		3 42
18 01506	-4 75	18 98426		3 39
23 8768	-0 625	24 89528		3 59
15 94798	-1 75	16 92813		3 77
17 01574	-5	17 97399	3 67	3 72
			3 96	4 08
			3 84	3 87
20 99384				3 36
				3 32
				3 93
				3 76
20 09583	-1 25	21 05407		3 64
				3 41
16 28474				
17 76044	-4 875	18 73785	3 57	3 63
17 61807	-2 375	18 59274		3 5
				3 41
	-2 5	19 43327	3 56	3 61
21 80424	-2 75	22 72416		3 82
18 65845	-3	19 62218	3 55	3 53
				3 62
				3 21
				3 57
				3 36
				3 88
23 00616				3 65

15 85216	-6 375	16 84326	3 5	3 46
22 17933	-6	23 16496		3 91
				3 44
24 48186				3 74
20 14784	-4 75	21 10609		
16 82683	-0 75	17 76044		
21 38261	0 875	22 3217		3 61
14 30801	0 5	15 31554	3 35	3 49
21 013	-2 75	22 05065		3 97
18 22313	-4 75	19 25804	3 87	4 06
			3 67	3 68
21 76044	-10 75	22 75702		
14 80082	-5 375	15 80287		3 68
				3 57
				3 87
14 423				3 49
16 87337	-8 5	17 88364		3 74
15 68515	-3 5	16 67899		
17 66735	-4 5	18 64476		3 61
15 85216	1	16 93087		3 91
			3 5	
19 2334	-5	20 21082		3 48
			3 35	3 52
15 56468	0	16 50924		3 29
12 39151	-1 375	13 39357	3 1	
18 47228			4 11	4 1
13 14442	-1 75	14 12183	3 13	3 13
			3 64	3 72
13 44285	-3 875	14 49144	3 21	3 3
			3 84	
			3 46	3 47
15 87953	-3 875	16 86516	3 65	3 74
			3 22	3 31
	0	18 31896	3 62	3 68
21 45106	-3 5	22 44216	3 99	3 94
			3 48	3 47
20 98563	-5 125	21 98494	3 8	3 89
14 3655	1 25	15 27995		3 44
				3 52
12 97741	-2 75	14 03149		3 21
				3 45
20 78576	-6 625	21 78508		3 46
				3 75
20 91444	-2	21 81793		3 45
				4 04
				3 41
20 6872				3 72
				3 85
				3 37
				4 08

			4 02
			3 56
			3 3
			3 42
20 94182			3 74
20 94182			3 6
20 93634	-4 625	21 95209	4 03
			3 47
20 84873			3 4
20 7885	1 5	21 77139	3 18
			3 48
	-0 5	20 6078	3 39
19 6386	-4	20 66256	3 62
19 68515	-0 75	20 69541	3 17
			3 65
			3 51
			3 6
			3 85
			3 44
	-4 75	21 12526	3 52
20 03833	-2 125	20 96646	3 72
20 07118	-2 625	21 05133	3 68
19 98631	-3 125	20 91171	3 27
			3 52
19 97536	0 25	20 98289	3 35
			3 64
19 88501			3 68
			3 81
18 61191	-1 625	19 5948	3 57
19 5729	-3 75	20 47639	3 95
19 63587	0 875	20 60233	3 5
	-0 125	20 53936	
19 48802	-1 5	20 41342	3 31
			3 93
			3 87
19 07187	-3 625	19 99179	3 69
19 06913	-4 625	20 00821	3 53
18 97057	-5	19 88227	3 89
17 76591	-3 25	18 79535	3 94
17 76318	-0 625	18 73511	3 48
			3 72
17 78508	1 625	18 77892	3 12
			3 69
			3 7
17 70568	-1	18 69131	4 09
			3 64
			3 51
			3 73
			3 5
			3 94
18 24778	1 125	19 21424	3 12
18 22313	-6 625	19 23066	3 48
	-5 625	18 88843	3 45

14 21218	-6 75	15 20055		3 41
16 79398	-3 625	17 78234		3 71
				3 42
16 60233	-8 75	17 60164	3 73	3 76
16 65435	-3 125	17 56331		3 76
17 56605	-3 125	18 50513		3 37
17 54962	-5 25	18 52704		4 15
17 48392				3 5
	-3 875	18 34908		3 47
				3 2
				3 76
17 32238	4 625	18 28884		3 44
17 27036				4 33
17 33333	-1 25	18 29158		3 7
				3 98
17 28679				3 43
17 11704	-0 875	18 09993		
	-3 875	18 19028		3 6
				3 16
				3 44
	-3 875	17 9822		3 53
16 95825	0 25	17 9165		2 9
	-7 125	17 8809		4 23
				3 47
14 88022	-2 125	15 89322		3 42
16 90897	-4 5	17 86174		4
16 90623				3 77
				3
15 79192	-0 625	16 78303		3 08
				3 81
15 77276				3 64
15 71253	-0 75	16 68446		3 01
				3 62
16 61602	-0 625	17 54962		3 42
16 3614	-4	17 37988		3 69
16 36687				3 67
16 28747	-0 875	17 31143		3 75
16 19712	-4 25	17 17454		3 88
				3 91
				3 81
				3 71
				3 61
16 10404	0	17 07871		3 55
	-8 75	17 09788		3 93
				3 85
				3 8
16 13689				3 19
				3 43
14 98426	-2 75	15 98905		3 21
15 91786	-2 75	16 8898		4 02
				3 17
15 60575	0 875	16 57769		3 66

15 91239	-0 625	16 85695	3 42
15 86585	-3 875	16 81588	3 5
14 84189	-2 5	15 8412	3 81
14 74606	-2 75	15 72622	3 57
14 76249	-3 5	15 75359	3 76
	0 5	15 79192	3 23
14 59822	-3 125	15 63313	3 16
14 61738	1	15 59206	3 26
14 66119	-2 875	15 55921	3 39
			3 54
14 55715	-3 75	15 52361	3 63
14 62834	-6 5	15 63587	3 49
14 81725			3 69
15 55099	-1 5	16 52841	
			3 6
15 47159	-3	16 4271	2 87
15 2909	-6 25	16 28474	3 74
15 41684			3 58
			3 47
			2 97
15 18412	-4 875	16 25188	3 39
15 17591	-4 375	16 15332	3 82
			3 62
15 24709	-3 375	16 23545	3 5
			3 7
15 26352	-5 375	16 2026	3 7
15 11841	0 25	16 09583	3 33
15 14853	-3 625	16 12594	3 66
15 1321	-4 875	16 10951	3 87
			3 64
	-3	16 01369	3 6
15 04723			
15 14305	0	16 04107	3 6
			3 18
14 91034			3 32
			2 96
14 96783	-4 875	15 94798	3 84
			3 31
			3 39
			3 57
14 84189	0 25	15 8193	3 5
14 88569	-2 25	15 85489	3 25
13 80698	-2 875	14 78439	3 33
			3 06
13 7577	3 875	14 75428	
13 76865	-5 875	14 81451	3 63
			3 8

	0 25	15 15674	
13 7796	-0 875	14 75428	3 68
13 6783	-8 5	14 63655	
13 73032	-3	14 66667	3 77
13 68104	-2 125	14 65024	
			3 28
	-2 375	15 52088	3 46
14 49144	-4 25	15 43874	3 23
14 49692	-1	15 44422	3 29
14 43943	-0 25	15 43053	3 34
14 42847	-4 625	15 42779	3 64
14 41205	-4	15 34292	3 77
14 31348	-0 875	15 31554	3 71
			3 34
14 39014	-1	15 39493	3 21
14 28063	-9	15 24435	3 5
14 20123	-0 75	15 17591	3 26
			3 3
14 19849			3 71
14 16564			3 47
14 14374	-1 25	15 0883	3 99
			3 73
14 01506	-6 75	15 02806	
13 99316	-2 875	14 97331	2 97
13 97125	-6 875	15 02806	3 81
			3 38
14 02053	-4	14 95962	3 4
13 90828	-4 5	14 86653	3 13
13 7577	-1 25	14 69678	3 41
	0	14 9514	3 35
	-0 625	14 89117	3 19
	-2 5	14 86379	2 8
13 87817	-0 375	14 88569	3 39
13 85353	-1	14 8063	3 63
			3 34
	-0 75	14 91581	3 06
12 80767	1 5	13 76044	3 01
12 75565	-2 5	13 7577	3 38
12 75838			3 6
12 73374			3 79
	0 5	13 80424	3 28
12 68994	-0 625	13 68652	3 52
			2 97
12 59685	-1 625	13 59069	3 58
12 56674	-2 75	13 57974	3 37
12 64613	-2 625	13 65092	3 18
			3 54

			3 38
			3 55
13 50034	-3 75	14 51061	3 34
13 56057	-2 875	14 55715	3 33
13 53046	-1	14 57084	3 25
13 52498			3 31
13 44832			3 72
			3 35
13 37988	-4 875	14 41752	3 33
13 37988	-1	14 3436	3 39
13 30048	-2	14 28611	3 47
13 26762	-5 25	14 28337	3 47
13 31417	-5 875	14 25051	3 73
13 34429	-4 875	14 34908	3 68
13 2731	-0 75	14 24504	3 57
13 27036	0 75	14 19849	3 31
			3 29
13 19644	-6	14 21218	3 26
			3 35
13 16906	1	14 11636	2 87
13 10062	0	14 05613	3 29
13 10335	-5 125	14 02601	3 59
13 10335	-5	14 09446	3 72
13 14716	0 5	14 1629	3 59
13 02122	-5 875	14 00137	3 76
			3 21
13 09788			3 53
			3 58
	-1 875	13 94387	3 37
12 95825	-4 125	13 93566	3 8
12 85421	-0 5	13 81793	3 61
12 91992	-3	13 90007	3 5
12 87337	-3 5	13 76865	3 8
12 85147	-4 5	13 84531	3 34
11 6961	-2 75	12 69815	3 01
12 55305	-5 375	13 53593	3 58
12 53388	-2 625	13 53593	3 38
12 62149	-1 375	13 58522	3 06
12 54483	-0 375	13 56331	3 4
12 42437	-6 5	13 44011	3 98
12 45448	-3	13 37714	3 8
12 36687			3 37
12 40246	-3	13 35524	3 41
12 3833	-7	13 32786	3 03

12 30664	-5 625	13 30322	3 28
12 27378			3 32
12 23546	-2 25	13 25394	3 42
12 17522	-4 375	13 18823	
12 23272	0 125	13 12799	3 45
12 15058	-2 625	13 18275	3 26
12 16975	1 5	13 1499	3 49
11 99452	-5 375	13 02122	3 4
	0	13 0486	3 61
11 98357	-2 625	13 01301	3 3
12 00548			3 21
12 01369			3 28
11 96988	-2 25	12 9911	3 22
11 95072	-0 5	12 96646	3 48
	1 75	12 95277	3 57
11 98631	0 5	12 92813	3 54
14 69952			3 71
16 36413	-4 5	17 42094	4 05
	-0 75	23 02806	3 47
22 68309	-6 25	23 62765	3 89
			3 39
14 42847	-5 5	15 34565	3 7
			3 25
			3 16
12 78576	-2	13 76044	3 31
	-1 5	16 25736	3 51
			3 41
13 95209	-3	14 96235	3 51
13 93292	-5 375	14 89665	3 32
12 95825	-6 625	13 95483	3 3
			3 5
16 08487	-2 25	17 08966	3 76
18 52704			3 71
	-0 75	13 70294	3 32
			3 13
20 4846			3 84
15 16222			3 55
19 04996			3 67
14 25599	-7 125	15 25257	3 34
13 88638	-3 75	14 88843	3 37
12 39151	-5	13 34976	3 4
16 65982	-3 5	17 67556	3 59
13 56057	2 75	14 58453	3 31
12 13142	0	13 11704	3 46
12 77755	-2 5	13 8152	3 47
12 55578	-11 25	13 61807	3 21
20 55578	0	21 48118	3 7
16 63792	-6 25	17 66187	3 5

11 72622	-4 875	12 69268	3 37
13 4976	5	14 4449	
	-4	15 42231	3 49
16 29021	-4 5	17 22656	3 64
15 21971	-5 375	16 21082	3 8
			3 68
13 59617			3 2
13 8809			
15 75359	-4 625	16 65982	
11 72622	0 75	12 75565	
11 36208	-4 625	12 34771	
11 53456	0 625	12 54209	
11 99726	-8 875	12 93635	
12 77207	-1 625	13 80698	
15 86585	-3 625	16 83504	
12 99384	-6 125	13 96578	
11 85216	-2 375	12 80219	
16 09309	-4 25	17 13347	
13 22108			
15 19507	-5 25	16 16153	
12 38604	0 75	13 37988	
15 85216	2 375	16 89802	
18 9076	-4	19 86584	
16 9911	-5 625	18 02875	
11 34565	-3 25	12 30664	
	0 125	13 53046	
15 99726			
11 50171	-1 125	12 5065	
16 50377			
11 15948	-7	12 2026	
14 17659	-4 5	15 16769	
15 26352	-8 125	16 19439	
18 1629			
10 82272			
10 1848			
13 65092	-4 75	14 66667	
11 92334	-0 5	12 9117	
9 52772	-2 75	10 56263	
15 16769	0	16 16701	
9 355236	5 375	10 35729	
9 442847	1 375	10 4668	
10 10814	-17 75	11 12115	
9 620808	-0 375	10 62012	
17 87817	-8 625	18 89117	

13 66188	-5 875	14 66119
11 52361		
11 96715	-6 5	12 9473
13 36345	-4 375	14 4011
8 91718	0	9 891855
	-11 625	11 17865
10 64203	-1 625	11 58111
8 914442	-3 875	9 878165
16 57495	-6 5	17 5551

master_acd_c	master_acd_c	master_acd_c	master_acd_c	master_acd_c	age
	3 89				
					15 17525
	3 73	3 76		3 74	13 87459
	3 63	3 65		3 62	
	3 98	3 99		4 08	15 60515
	3 7				
	3 36	3 39		3 35	11 18017
	3 67		3 54	3 44	
	3 46	3 52			
	3 71			3 23	
	3 79		3 7	3 66	
	3 56		3 58	3 58	13 31599
	3 52	3 59		3 61	
	3 67	3 67		3 51	
	3 77	3 8		3 72	13 59803
	3 49	3 51			
	3	3			
	3 49	3 48		3 52	15 16429
	3 73	3 73		3 66	16 38554
	3 68	3 68			17 97919
	3 22				
	3 43	3 44		3 46	15 95564
	3 49	3 52			
	3 25	3 3		3 37	
	4 04	4 08		4 09	
	3 42				14 07722
	3 71	3 7		3 74	
	3 65	3 52		3 63	
	3 6	3 62		3 4	17 49726
	3 92	3 87			
	3 83		3 84	3 78	13 69387
	3 84				
	3 82	3 73		3 72	
	3 64	3 67		3 59	
	3 59				15 94195
	3 72	3 7		3 7	
	3 79	3 89		3 81	17 07284
	3 39		3 41	3 48	
	3 38	3 42		3 46	
	3 59	3 61		3 59	14 94524
	2 86				
	4 16				
	3 63	3 66		3 65	10 7092
	3 85	3 85		3 86	

3 26	3 28		3 24	
3 93	4 02		4 07	11 64294
	3 37		3 39	11 72234
3 38	3 32		3 29	
3 4	3 47		3 6	
3 55	3 55		3 54	13 50767
3 81	3 83		3 81	13 13801
3 36	3 37		3 39	
3 94	3 98		3 92	14 99726
3 38	3 43		3 4	
3 89	3 89		3 89	
3 4	3 43		3 4	
				16 45674
3 11				
3 49		3 54	3 54	15 25465
3 99	4 05		4	11 27054
3 82	3 92			10 85706
3 87	3 68		3 94	14 76177
3 81				17 92169
3 71	3 79		3 72	16 78806
3 65			3 67	
3 79	3 84		3 84	14 35378
3 51	3 68		3 63	13 64458
3 06			3 12	10 87349
3 77	3 77		3 74	
3 85	3 87		3 8	13 20099
3 67				
4 23	4 3			11 87842
3 52	3 46			
4 19	4 1		4 1	11 84283
				17 11391
3 59	3 57			17 14403
3 8	3 75		3 74	17 85597
3 9	3 81			
	3 63			
4 1	4 01			
				15 27656
3 8				16 87568
3 95	3 88			
3 78				
3 54	3 78		3 83	
3 68	3 69		3 69	
4 01	4 01		3 86	

3 02		3 03	3 08	12 14951
3 66	3 72		3 69	11 56079
3 45	3 62		3 65	10 70099
3 7				13 26123
3 31		3 06	3 06	16 92497
3 87				
3 63	3 64		3 59	
3 73				
3 64				
3 46	3 47			16 07339
3 62				
3 33				
2 87	2 91		2 91	10 95564
3 78	3 85			14 75356
3 91	3 86		3 84	12 71632
3 45	3 77		3 75	14 11829
3 32	3 3			17 23439
3 62	3 68			
4 12	4 14			
3 63	3 49			15 2437
3 5	3 54			17 83406
2 93	2 88		2 68	
3 92	3 97			12 26999
3 7	3 72		3 77	14 18127
3 06				10 62432
3 66	3 7			
				15 71468
3 45	3 59		3 64	10 64075
3 77	3 66		3 7	
3 9	3 93		3 93	17 68072
4 02	3 97		4 03	
3 62	3 62		3 6	
3 49	3 49		3 51	11 80723
3 38	3 36		3 39	
3 44			3 48	
3 32				
3 85		3 88	3 89	
3 41	3 4		3 42	12 32749
3 54	3 6		3 56	11 91128
	3 16		3 11	16 1172
3 91	3 98			11 36364
3 53	3 56		3 65	
3 22	3 27		3 22	12 86692
3 51	3 52			11 86199
3 54	3 47		3 47	

2 89	2 82		2 89	12 75192
3 67		3 73	3 73	14 30997
3 89	3 89		3 91	16 50876
3 73	3 77		3 71	
4 03		4 05	4 05	
4 02			4 13	16 26232
3 29	3 3		3 2	11 3828
3 52				15 56955
4 12	4 11		4 19	
3 44		3 47	3 47	
4 07	4 09		4 17	
3 8	3 8		3 85	12 91073
3 36	3 36			
3 79	3 91		3 84	14 49069
3 87	3 89		3 92	15 16429
3 64	3 6		3 61	12 66156
3 7				
3 28	3 39		3 38	13 27492
3 58	3 63		3 56	14 94797
3 87			3 91	11 59091
3 67	3 69		3 69	
3 78	3 75		3 88	12 39047
3 82		3 85	3 85	16 82092
3 7	3 78			16 96878
3 22	3 22		3 1	
3 6			3 58	
4 09	4 14			16 0816
3 82	3 91		3 74	10 70646
3 63	3 64		3 39	13 59529
3 42	3 54		3 49	11 89759
3 52	3 49		3 43	
	3 82			
3 42	3 37		3 39	
4 09	4 11		4 07	
3 49	3 51		3 45	14 58379
3 69	3 73		3 75	13 74589
4 16	4 17		4 2	
4 05	4 08		4 04	
3 52	3 56		3 61	
3 95				17 00986
3 43	3 46		3 47	
3 75	3 7		3 76	
3 21				
	3 75			
3 52	3 62		3 62	12 09474
3 74	3 8		3 73	

3 73	3 78			10 8023
3 66	3 65		3 61	16 72782
				17 91895
4 02	4		4	
3 9	3 88		3 95	
3 88	3 92		3 77	
3 73				15 58324
4 1	4 09		4 16	
		3 46	3 46	
3 08	3 15			
3 69	3 65		3 65	12 93264
3 61	3 61		3 62	12 94085
3 67	3 67			14 477
3 86	3 88			11 07065
3 71				12 57667
3 68	3 65	3 6	3 64	
3 41	3 47		3 5	12 26177
4 02	3 95		4 01	13 02848
3 58	3 55		3 55	
3 59	3 54		3 56	12 6506
3 5	3 51		3 49	10 70099
3 75	3 75			
3 84		3 86	3 86	
3 1	3 09		3 15	
3 4	3 42		3 43	
3 45	3 47		3 52	
3 38	3 39		3 45	13 53779
3 71	3 75			
3 86			3 82	
3 97		3 91	3 91	16 73877
3 4			3 5	
3 69	3 73			
3 58	3 58		3 56	10 65444
3 46	3 41		3 43	
3 48	3 54		3 58	
3 89	3 88		3 89	
3 9	3 91		3 9	
3 59	3 6		3 6	11 88938
				11 01588
	3 32			10 87623
3 53	3 54		3 54	
3 74				
3 42	3 35		3 35	16 79902
3 53	3 55		3 62	11 62651
				15 51205

3 08				
3 99	4 05		4 08	12 21796
3 62	3 59		3 58	14 40307
3 52	3 43		3 56	
3 49	3 46		3 54	
3 54	3 54			13 21194
3 74	3 8		3 78	13 3598
3 62	3 6		3 62	
4 06	4 1		4 04	
		3 47	3 47	
3 79	3 82		3 73	
	3 29			13 66648
3 5	3 42		3 49	
3 4	3 4		3 38	12 96276
3 78	3 82		3 78	11 16375
3 3				17 72453
3 27	3 28		3 3	
3 61	3 63			
	3 69		3 67	
3 68	3 65			13 98686
3 35	3 3			
3 33				16 46769
3 56	3 57			
3 79	3 74		3 61	
3 77	3 79		3 8	
3 16	3 44		3 3	
3 63	3 62		3 51	
3 81				15 64075
				17 58762
3 23	3 44		3 41	10 73932
3 88	3 93		3 88	13 6172
3 72			3 67	
3 72				
3 66				12 76013
3 38				
3 9				13 13527
3 77	3 81		3 89	
3 71	3 73		3 74	
4 15			4 28	14 63855
3 87				
	4 1		4 19	15 24096
3 89	3 86		3 87	16 61008
3 96	3 98		3 99	
3 5	3 56		3 43	11 4184
3 88	3 86		3 92	

3 7	3 68			
	3 82			15 65991
3 72	3 75		3 73	12 56024
3 56			3 58	
3 64				16 07065
3 54	3 65		3 59	13 06681
3 66	3 79			
3 64		3 64	3 64	10 96112
3 72	3 74			12 80942
				17 0345
3 64			3 54	
3 59	3 54		3 66	
3 67	3 73			
3 7	3 65		3 71	
3 94	3 94		3 95	11 01588
3 09	3 18			
3 75	3 72		3 7	15 60515
3 73	3 76		3 76	10 92826
3 43	3 42		3 43	17 41785
3 82	3 84		3 86	
3 55				
3 99	3 95		3 91	
3 62	3 61			15 94469
3 66	3 68		3 66	14 70153
3 86	3 87		3 87	11 52245
3 67				
3 43		3 4	3 42	11 84009
3 5				
3 74		3 88	3 79	16 74151
3 7	3 7		3 72	16 70318
3 65	3 66		3 66	14 63034
3 81		3 85	3 85	
4 02		4 06	3 99	
3 47	3 52			
3 39				
3 46	3 51		3 41	11 90307
3 72	3 77		3 7	11 62924
3 36	3 28		3 4	
3 47	3 47		3 46	
3 43	3 42		3 37	
3 35	3 37		3 37	
3 72	3 75		3 75	12 72727
3 41			3 39	13 56791
3 68				13 26123
3 98	4 04		4 09	12 19332

3 55		3 58	3 6	14 76177
3 22	3 56		3 22	10 8023
3 81	3 93		3 88	13 37897
3 77	3 85		3 51	
3 8	3 81		3 8	
4	4 03		4 12	17 64239
3 54	3 62		3 65	
3 54				
3 82	3 82		3 86	15 74206
3 5	3 56		3 56	13 76232
3 64				
2 93	3 24		3 26	12 51643
3 57	3 63			11 97426
3 94	3 91		3 89	15 37788
4 11	4 17		4 1	11 54436
		3 58	3 58	
3 7				
3 74			3 69	
4 02	3 93		3 96	
4	3 95			
3 18	3 51			
3 84	3 89		3 91	12 74096
	3 22			17 15224
3 75	3 77			
3 83	3 87		3 87	13 58981
3 58	3 75		3 69	11 977
3 28	3 24		3 23	
	3 28		3 26	
3 65	3 9		3 84	11 73056
3 69	3 59		3 66	
3 34	3 34		3 34	
	4 04		4 05	11 55257
3 34	3 31			12 10843
3 81	3 84		3 84	10 83516
3 82	3 9		3 89	
3 94	3 89		3 94	15 18072
3 47	3 59		3 57	
2 85	3 05			13 77054
3 56	3 63		3 59	
3 68	3 65		3 69	
3 53	3 58		3 58	10 64075
3 42	3 45		3 58	
3 97		4 04	4 01	
3 39				15 1506
3 82	3 81		3 8	12 88609
3 25	3 15			
3 78	3 84		3 84	12 29189
3 48			3 55	14 21961

2 96		2 87	3 01	10 49014
3 84			3 84	17 08927
3 86				
3 61	3 59		3 59	14 93702
3 67	3 7			13 74042
3 58	3 55		3 62	
			3 51	11 20756
3 55	3 61		3 65	10 3149
3 61			3 24	13 15991
3 53	3 55		3 52	
4 01	4 01			
4 15	3 98		4 12	
3 32	3 37		3 36	
3 85	3 9		3 9	
			3 62	
3 85		3 89	3 85	
				9 548193
3 36	3 39		3 27	11 79901
3 51	3 51		3 49	
3 68	3 63			12 87514
3 74	3 77			16 92497
3 53	3 55		3 5	
4 09	4 06		4 06	11 63472
				16 35816
3 92	3 96		3 93	
3 53	3 57			11 16922
3 71	3 75		3 75	
3 6	3 62		3 71	9 696057
4 06				
3 37	3 44			13 65279
3 86	3 93			
3 67	3 67		3 75	11 56353
3 74	3 75			
3 29	3 3			
3 62	3 62			15 79956
3 75	3 72		3 74	15 13417
3 89			3 94	10 88992
3 56			3 56	17 26725
3 55	3 54		3 6	10 84337
3 56	3 55		3 59	
3 71		3 76	3 7	
2 84	2 87		2 88	11 21303
4 18	4 27		4 29	17 06462
3 96	3 9		3 95	
3 53	3 56			
3 61	3 62		3 56	

			3 35	
3 89	3 9		3 87	
3 74	3 66		3 78	11 7333
3 84	3 79			14 71796
3 76	3 76		3 75	
3 34	3 45		3 37	9 994524
3 92	3 94		3 85	14 80285
3 91	3 97		4 01	
3 25	3 26		3 27	12 14129
3 82	3 81		3 82	
3 78	3 82		3 83	11 53067
3 98	3 98			16 78259
3 56	3 66		3 73	
3 57	3 57		3 59	12 62048
3 33	3 38		3 29	
3 8	3 75		3 81	14 69606
3 61	3 63		3 66	13 32694
				16 55531
3 18		3 41	3 46	
3 79		3 77	3 77	15 10953
2 98	3 08		3 04	
3 65	3 7		3 76	14 4414
3 72	3 75		3 79	12 8943
3 4	3 52			
3 85	3 85		3 88	
	3 71		3 79	
3 6	3 67			10 83516
3 7	3 78			10 21358
			3 96	14 69606
3 58	3 58		3 61	
3 74	3 76			14 27163
3 5	3 58		3 56	
3 5	3 52			11 45126
3				12 03176
3 67	3 72		3 51	
	3 69			17 59584
3 43	3 67		3 73	
4 01	4 07		4 05	15 2793
3 59	3 63		3 7	
3 82	3 8		3 83	
4 32	4 35		4 34	
3 7				12 5575
4 01	4 03		4 09	
3 72				17 17415
	3 62			

3 51	3 49			15 38883
3 98				17 76561
3 67	3 65		3 67	15 67087
3 66	3 76		3 86	
3 71		3 71	3 76	11 08981
		3 52	3 52	12 03176
3 92		3 94	3 94	12 64786
3 53	3 56		3 52	17 57393
3 52	3 71		3 47	17 30285
3 63	3 57			
4 05	4 07		3 97	13 45838
3 6	3 63			
3 36	3 4			
3 27				
3 59	3 6		3 66	
4 2	4 14		4 11	10 70646
3 59	3 74		3 75	
3 33	3 36		3 35	
3 96	3 96		3 96	10 99124
3 65	3 7		3 67	10 57503
3 49	3 43			
3 61	3 65		3 71	
3 93	3 98			13 63363
3 47	3 45		3 54	12 64513
3 83	3 85		3 76	
3 84	3 93		3 89	
3 46				
3 69	3 81		3 81	
3 68	3 57		3 57	
3 52	3 6			
				12 77382
3 69	3 77		3 76	
3 73	3 75		3 74	
3	3 11		3 35	
3 62	3 72		3 72	
3 39	3 35			
			3 31	
	3 64		3 65	
3 92	3 83		3 81	14 93702
3 75	3 77		3 72	13 78149

3 29	3 49	3 49	13 546
3 78	3 76	3 76	
3 69	3 69	3 8	
2 72			
3 65	3 64	3 68	
3 94	3 83	3 85	
3 43			13 80613
3 39		3 37	
3 82	3 88	3 92	
3 25	3 27	3 31	
3 7	3 73	3 82	
3 21	3 42	3 41	
3 74	3 76	3 89	
3 86	3 97	3 99	
3 56	3 89	3 88	
3 6	3 57	3 56	
3 29	3 36	3 34	9 679627
3 11	3 23	3 09	
3 85	3 82	3 73	
3 45	3 41	3 16	14 74261
3 73	3 76	3 8	
3 88	3 88	3 83	
3 55			
3 49	3 51	3 44	
3 77	3 88	3 88	
		3 35	9 006024
4 07	4 11	4 04	
3 48	3 61	3 6	
3 39	3 45	3 43	13 49398
3 75	3 78	3 77	
4 13	4 15		9 279846
	3 65	3 74	
3 66	3 72	3 62	
	3 46	3 46	
3 5	3 49	3 55	
3 44	3 51	3 54	10 0931
3 8	3 62	3 72	
3 92	3 94	3 94	16 44578
3 73	3 7	3 75	10 71194
3 64	3 67	3 72	
3 55	3 54	3 55	9 236035
3 79	3 86	3 87	9 263417
3 7			
3 68	3 69	3 62	

3 61				15 78587
3 54	3 54		3 63	
3 61		3 75	3 75	
3 26	3 3		3 31	
3 82	3 7		3 76	
3 52	3 54		3 56	
3 51		3 65	3 63	
3 77	3 8		3 78	
3 75	3 85		3 9	9 318182
3 66			3 7	
3 8	3 7			12 00164
3 73	3 76		3 73	
3 24	3 27		3 23	
3 72	3 75		3 62	10 99124
3 65	3 71		3 38	
3 59	3 54		3 57	
3 69	3 81		3 81	
3 72	3 68		3 64	16 05422
3 3				
3 41	3 51		3 57	
3 64		3 65	3 69	
3 5	3 56		3 58	17 63691
3 73	3 76		3 72	13 73768
	3 67		3 72	10 77492
3 82	3 74		3 8	13 29682
3 63				
3 28		3 26	3 26	
3 43	3 4		3 48	11 04053
3 66	3 72		3 65	10 91183
3 73	3 75		3 83	
				13 72399
3 51	3 47		3 52	
	3 98		4 04	
2 82	2 83			
3 53	3 58		3 54	17 87787
3 55		3 44	3 44	16 94414
4 11	4 05		4 05	
3 67	3 7		3 73	13 57065
3 63	3 64		3 6	10 65444
				16 97152
4 04	4 06		4 04	10 01369
				17 66977
3 41	3 5			
3 79	3 82		3 81	11 01862
		3 34	3 31	12 06736
3 44	3 37		3 44	17 2782
				16 64293
3 51	3 54		3 54	14 88773

	3 54			
3 87	3 96		3 98	
3 52	3 77		3 74	13 71851
3 82	3 77		3 86	12 20427
3 87				14 80559
3 95	3 93		3 94	17 58762
3 86				10 19441
3 57	3 58		3 55	15 8954
3 3				
3 73	3 75		3 76	9 260679
3 56	3 57			
2 79	2 87		2 81	
3 04	3 1	1 99	3 15	12 1276
3 72	3 78		3 79	9 972618
3 38	3 44		3 06	10 49288
3 71	3 75		3 78	13 05586
3 59	3 6		3 58	13 0805
3 99	3 92		4 01	
3 75	3 82		3 85	13 09967
3 65				
			3 96	15 72837
3 43	3 55		3 58	
3 34	3 31		3 34	
3 45	3 45		3 45	16 23494
3 88	3 94		3 94	9 578313
3 39	3 64		3 75	9 162103
3 43	3 45			17 29737
3 66	3 63		3 69	
				9 594743
3 59				
3 81	3 83		3 7	16 26506
3 32	3 31		3 38	
3 4	3 46		3 51	12 56572
3 48	3 52		3 61	9 285323
3 49		3 49	3 49	12 87514
3 53	3 54		3 53	14 18675
3 56	3 57		3 6	15 92004
	3 68			15 06024
3 73	3 72		3 61	10 22727
3 88	4 04			
3 53	3 79		3 84	13 52957
3 19	3 68			12 95455
3 66	3 64		3 64	15 63801
3 86	3 87		3 79	
3 42	3 44		3 48	10 38883
3 41	3 34		3 36	11 81271
3 58		3 6	3 57	10 3368
3 66	3 69			17 95181

3 31		3 33	3 33	12 6506
3 39	3 4		3 43	
3 93	3 84		3 88	13 1517
4 24		4 2	4 2	13 75685
3 66	3 71			9 189486
				11 23768
3 83	3 81		3 8	9 389376
3 78			1 59	13 18182
	3 85			
3 61	3 6		3 58	10 19168
3 32	3 68		3 38	
3 66	3 75		3 75	
3 79	3 87		3 91	10 39704
3 88	3 89		3 95	
3 96	3 98		3 99	
	3 58		3 66	9 627602
3 64			3 5	
	3 73		3 74	
3 8	3 85		3 88	
3 51	3 6		3 58	
3 76	3 79		3 83	10 92552
3 48	3 51		3 62	
3 32	3 37			
3 67	3 7		3 72	
3 46	3 48		3 5	14 27163
	3 58		3 67	15 12322
3 55	3 6		3 59	13 40361
	4 08		4 09	
3 21	3 26		3 28	
3 56		3 59	3 51	
3 67		3 64	3 67	
3 73	3 75		3 79	15 81051
3 28		3 35	3 35	14 96988
3 43				
3 47	3 46		3 51	11 90033
3 51	3 52		3 54	14 70427
3 25	3 5		3 62	
3 3				15 78587
3 81			3 98	10 19715
3 75	3 77		3 71	
3 78	3 78		3 77	
3 71	3 71		3 7	16 09803
3 61	3 69		3 72	11 99069
3 35	3 4			12 50274
3 4	3 68		3 34	
2 94	3 16			
3 46	3 48		3 62	9 071741
3 21	3 24		3 19	12 94359
3 7			3 65	13 29135
	3 7		3 76	

3 66	3 75		3 68	10 68456
3 45	3 61			
				12 93812
3 49	3 53		3 59	
4 14	4 17		4 12	12 71906
3 2	3 38		3 22	
	3 5		3 65	
3 85	3 84		3 82	12 14951
4 06				12 56572
3 81				
3 54	3 67		3 68	9 290799
3 37	3 32		3 35	
3 35		3 41	3 43	12 7793
3 67	3 67		3 73	13 37076
3 84	3 81		3 82	
3 92				12 68346
3 97				15 87349
2 9		3 32	3 31	
2 94				9 56736
3 78	3 82		3 83	
3 42				14 49343
3 43	3 53		3 52	16 04326
	3 67		3 65	
3 76	3 72			
3 46	3 46		3 52	11 44304
3 39			3 46	
3 64	3 59		3 62	
3 82	3 86		3 85	10 90909
3 79	3 74		3 69	11 98795
4 09	4 11			13 25027
3 85	3 87		3 82	14 58653
3 37	3 36		3 36	
3 49	3 57			
3 92				
3 69				
3 64	3 67			15 07393
3 43	3 44			11 78259
			3 8	
3 58	3 67		3 63	
3 59	3 59		3 63	
	3 38			
3 6	3 67		3 67	
3 84	3 82			16 7333
3 62	3 65		3 7	13 56791
	3 51			
3 29	3 48			
3 36				
3 91				
	3 68		3 72	17 93538

3 56	3 55		3 58	
	3 92		3 96	17 0701
3 3				
3 63	3 63		3 56	
3 8				
3 56				16 34447
3 5	3 58		3 63	
3 92	3 99		3 94	
4 06				
3 72	3 75			15 98302
3 65	3 65		3 66	
3 59	3 7		3 76	
3 57	3 61		3 64	
3 86	3 91			
3 43	3 51		3 55	
3 9	3 88		3 8	
3 49	3 48		3 49	
3 59	3 54		3 65	
3 64	3 65		3 6	
3 96	4 08		4 14	
			3 48	
3 43	3 43		3 47	
		3 56	3 56	
3 29	3 42		3 48	
3 56	3 62		3 75	
4 18	4 1		4 07	13 431
3 24	3 26		3 27	
3 77	3 77		3 76	11 09803
3 32	3 44		3 54	
3 85	3 81		3 8	
3 53	3 5		3 47	11 01588
3 73	3 73		3 87	
3 26				15 34228
	3 69			12 25356
3 92	3 99		3 97	16 37185
3 49	3 43			17 15224
3 9	3 92		3 95	15 89814
3 46	3 55		3 63	
3 54	3 62			
3 27	3 43		3 35	
3 48				10 55586
3 55	3 55		3 51	
				15 73932
3 46	3 47		3 43	
4 06	4 1			15 7448
3 8		3 9	3 95	15 55586
3 97	3 97			15 54765
3 37	3 39			15 58872

4				
3 57				
				15 95564
3 78	3 82		3 76	15 89814
3 56				15 87076
4 12	4 2		4 04	15 82694
				15 77492
3 27	3 26		3 26	
3 55		3 55	3 55	14 72618
3 41				14 57284
3 68	3 68		3 68	
	3 19			14 56462
3 48		3 61	3 51	15 21084
3 51				
3 81				15 16977
3 69	3 61		3 66	15 06024
3 55	3 56			15 03286
3 72	3 72		3 75	14 93428
3 67	3 68		3 69	
3 32	3 34		3 26	
	3 35			14 8713
3 55				
3 65	3 73		3 74	14 80285
3 62	3 68		3 64	13 546
3 88	4		3 95	
3 58	3 59		3 67	
3 28				
3 41		3 44	3 46	14 38664
3 9	3 86		3 91	
3 75	3 76		3 8	13 99507
3 56	3 6		3 6	13 99233
3 86	3 91		3 92	13 83899
3 72	4 02		4 01	12 73001
3 59	3 67		3 67	12 76561
	3 81			
2 95	3 25		2 93	12 73823
3 71	3 72			12 68346
4 09	4 08		4 07	12 68072
				13 53231
3 59	3 59		3 61	12 51369
3 71	3 78		3 79	13 49124
	3 88		4 01	
3 13		3 12	3 15	
	3 55			
3 45	3 43		3 45	12 87514

3 51	3 52		3 53	9 205914
3 8	3 74		3 87	11 74151
3 5	3 48		3 41	
3 73	3 68		3 71	
3 8	3 81		3 81	
		3 62	3 62	
4 18	4 2		4 22	12 53833
3 55	3 53			
3 55	3 53		3 55	12 30285
3 83				
3 57	3 49		3 59	
4 42	4 4			12 24808
3 66	3 64		3 68	
3 97	3 91			12 12212
3 42				12 22344
			3 33	
3 63	3 75		3 73	12 19058
3 18	3 2		3 22	12 00438
	3 56		3 65	
3 67	3 68		3 74	
			2 96	11 90854
4 15	4 15		4 18	11 89485
3 47	3 53			11 93319
3 55	3 59		3 6	
3 99	4 04		3 96	11 85652
3 85	3 83		3 82	11 83735
3 1	3 17		3 19	
3 9	3 9			10 69551
3 74	3 8			10 64348
3 18	3 18		3 26	
3 7				
3 64				
3 74	3 69		3 79	
3 76	3 8		3 8	11 33899
3 87	3 91		3 92	11 29244
3 97	3 96		3 9	
3 84	3 79		3 88	11 18291
3 74	3 82		3 86	11 35816
3 65		3 68	3 68	
3 98	4 02		3 98	
3 92	3 95		4	
3 73	3 72		3 75	
3 17	3 26		3 24	
		3 5	3 56	
	3 49			9 912376
3 98	4 02		4 03	
3 25	3 29		3 34	10 86254
3 62	3 68		3 7	

3 49	3 49			
3 59	3 62		3 65	10 79135
3 87	3 87		3 86	9 75356
3 65	3 68		3 8	9 696057
3 84	3 87		3 96	9 696057
3 3	3 36		3 36	
3 27	3 36		3 3	9 56736
3 07	3 35		3 34	9 58379
		3 69	3 76	9 561884
3 39				9 542716
3 75	3 86		3 94	
3 53	3 47		3 58	
	3 77		3 82	
4 03	4 14		4 14	
3 7				
2 83	3 07			10 46276
3 77	3 79		3 82	10 27382
3 79	3 79		3 92	10 37788
3 04				10 19441
3 17	3 47			
3 91	3 94		3 92	
3 59	3 62		3 65	10 11774
3 51	3 61		3 67	
3 77	3 72			10 19168
3 75	3 74		3 78	
3 38	3 42		3 44	10 09036
3 7	3 75			
3 11	3 26		3 12	
3 6				10 02464
3 22				9 887733
			3 54	
3 01	3 05			9 956188
3 9	3 89		3 94	
	3 31			9 824754
3 4				
3 67	3 69			
3 63	3 57		3 66	
3 27	3 31		3 4	9 824754
3 34			3 5	
3 29	3 36		3 44	
3 7			3 95	8 751369

3 37				
3 74	3 81		3 85	
3 81	3 85		3 93	
3 84	3 86		3 96	
3 52			3 7	
3 31				
3 36			3 35	9 479737
3 51	3 51		3 57	9 468784
3 21	3 43		3 56	9 466046
3 34	3 4		3 45	9 416758
3 79			3 99	
3 88	3 87			9 296276
3 66	3 79		3 81	9 288061
			3 62	
3 29				9 345564
3 53		3 55	3 51	
3 4	3 42		3 6	
3 67	3 79			9 134721
				9 066265
3 78				9 055312
3 76	3 76			
4 09	4 12		4 16	
2 99	3 12		3 21	
	3 95		3 89	
3 38	3 4			
3 34	3 54		3 69	
3 46	3 41		3 46	
	3 58		3 6	
3 29	3 41		3 55	
2 95				8 797919
3 46	3 57		3 48	
3 59				8 748631
3 26	3 23		3 19	
3 45	3 59		3 55	
	3 69			
3 82	3 89		3 9	
3 25	3 35			
3 54			3 59	
	3 08			
3 55				
3 38	3 6		3 57	
3 26	3 39		3 5	
3 7				

3 43			
3 46			3 57
3 31	3 48		3 55
3 4	3 52		3 5
3 28	3 35		3 44
3 39	3 37		3 42
	3 94		3 89
3 51	3 64		3 65
3 5		3 57	3 57
3 43			
3 54	3 66		3 67
3 59	3 65		3 71
3 8	3 91		3 92
3 76	3 86		3 92
			3 73
3 22	3 28		
3 39			3 5
3 34			
3 31	3 36		3 42
3 35	3 39		
2 77	3 11		3 17
3 43	3 48		3 52
3 58	3 8		3 86
3 85	3 9		4 01
3 64	3 76		3 72
3 81	3 83		3 89
3 21	3 21		
3 63	3 72		3 72
	3 72		
3 91	4 05		4 02
3 63	3 65		3 68
3 53	3 47		3 54
3 93	3 9		3 96
3 41	3 43		3 5
3 03	3 17		3 25
3 61	3 75		3 82
3 59	3 74		3 8
	3 18		
3 52	3 52		3 5
4 04	4 2		4 24
	4 02		4 04
3 37	3 4		3 47
3 44	3 53		3 62
3 13	3 33		3 38

3 37	3 44		3 58	
3 44		3 48	3 48	
3 54	3 56		3 6	
3 52		3 66	3 66	
			3 46	
3 54	3 52		3 58	
3 3	3 37		3 49	
3 53			3 59	
3 54	3 57		3 65	
3 58	3 56			
3 18	3 59		3 47	
3 31		3 41	3 41	
3 34	3 4		3 49	
3 56	3 6		3 69	6 878423
	3 68		3 7	
		3 64	3 64	
3 82	3 82		3 84	9 676889
4 03	4 12		4 11	
	3 52			16 97426
3 88	3 9		3 81	
3 48				
3 78	3 75		3 77	9 356517
3 33				
3 24	3 23		3 25	8 918401
3 46	3 52		3 39	
			3 64	
3 45				
3 57	3 61		3 61	
	3 44		3 5	
3 4	3 45			7 965498
3 43	3 6			8 986856
3 85	3 9		3 9	
3 79	3 82		3 91	13 40909
3 53	3 64		3 8	
	3 33		3 29	
3 87	3 92		3 89	
3 6				
3 68				
3 4	3 43		3 38	
3 26	3 52		3 54	
3 56	3 65		3 72	
3 62	3 62		3 62	
3 31	3 57		3 26	
3 55	3 56		3 63	
3 55		3 62	3 67	
3 35	3 33		3 24	
3 79			3 78	15 56955
3 59	3 51		3 55	

3 49	3 56	3 6	
3 49	3 43	3 58	
3 54	3 59		
3 66	3 73	3 74	
3 83	3 91	3 9	10 30668
3 67	3 75	3 89	
3 14	3 19	3 27	
3 68	3 65		
3 85	3 83	3 93	
3 48	3 71	3 67	
3 18	3 22	3 22	
3 64	3 69	3 74	
3 23	3 17	3 42	
4	4 17	4 12	
3 4	3 51	3 63	
3 77	3 87	3 77	
3 43	3 49	3 41	
3 12	3 28	3 39	
3 88			
3 87	3 86	4 01	
3 79	3 82	3 79	
3 46	3 53	3 59	
	3 38		
3 4	3 42	3 47	
2 94	2 88	3 01	
	3 42	3 55	
3 61	3 59	3 61	
2 98	2 97	3 26	
3 55	3 54	3 44	
3 63	3 28	3 59	
3 01		3 03	
3 33	3 42	3 41	
3 29	3 29		
3 23	3 32		
3 04		3 15	
		3 44	
		4 1	
		3 9	
		4 2	
		3 16	
		3 35	
		3 84	
		3 1	
		3 79	
		3 43	
		3 39	
		3 46	
		3	
		3 81	
		3 33	
		3 72	

3	71
3	49
3	74
3	78
3	22
3	78
3	48
2	48
3	91
